# *De novo* design of type II topoisomerase inhibitors as potential antimicrobial agents targeting a novel binding region†

**DOI:** 10.1039/d2md00049k

**Published:** 2022-06-16

**Authors:** Kyle M. Orritt, Lipeng Feng, Juliette F. Newell, Jack N. Sutton, Scott Grossman, Thomas Germe, Lauren R. Abbott, Holly L. Jackson, Benjamin K. L. Bury, Anthony Maxwell, Martin J. McPhillie, Colin W. G. Fishwick

**Affiliations:** School of Chemistry, University of Leeds Leeds LS2 9JT UK M.J.McPhillie@leeds.ac.uk C.W.G.Fishwick@leeds.ac.uk; Dept. Biochemistry & Metabolism, John Innes Centre Norwich Research Park Norwich NR4 7UH UK Tony.Maxwell@jic.ac.uk

## Abstract

By 2050, it is predicted that antimicrobial resistance will be responsible for 10 million global deaths annually, more deaths than cancer, costing the world economy $100 trillion. Clearly, strategies to address this problem are essential as bacterial evolution is rendering our current antibiotics ineffective. The discovery of an allosteric binding site on the established antibacterial target DNA gyrase offers a new medicinal chemistry strategy. As this site is distinct from the fluoroquinolone binding site, resistance is not yet documented. Using *in silico* molecular design methods, we have designed and synthesised a novel series of biphenyl-based inhibitors inspired by a published thiophene-based allosteric inhibitor. This series was evaluated *in vitro* against *Escherichia coli* DNA gyrase and *E. coli* topoisomerase IV with the most potent compounds exhibiting IC_50_ values towards the low micromolar range for DNA gyrase and only ∼2-fold less active against topoisomerase IV. The structure–activity relationships reported herein suggest insights to further exploit this allosteric site, offering a pathway to overcome developing fluoroquinolone resistance.

## Abstract 2

A computational design, make and test strategy was used to identify antibacterial inhibitors of bacterial DNA gyrase and topoisomerase IV, proposed to bind at a novel allosteric site.

## Graphical abstract

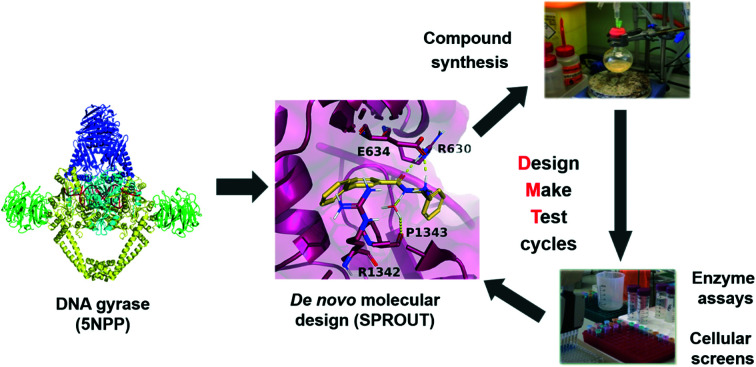

## Introduction

The evolution of antibiotic resistance poses an enormous threat to human health.^1^ The discovery of penicillin in 1928 heralded the beginning of the antibiotic era, revolutionising the treatment of bacterial infections. Antibiotics then became a staple in modern medical procedures such as surgery and organ transplantation. However, following the golden period of antimicrobial drug discovery between the 1940–80s, a decline in novel antibiotic FDA approval coupled with a rise in antimicrobial resistance (AMR), has led to an increase in the number of untreatable bacterial infections.^2^

DNA gyrase and topoisomerase IV are essential bacterial type II topoisomerases that control DNA topology during DNA replication, transcription and other DNA-associated processes.^3,4^ DNA gyrase introduces negative supercoils into bacterial DNA *via* an ATP-dependent mechanism, whereas topoisomerase IV primarily eliminates DNA entanglements that occur during DNA replication. Both enzymes are composed of two proteins, coded for by the *gyrA* and *gyrB* genes for DNA gyrase, and the *parC* and *parE* genes in the case of topoisomerase IV.^5,6^ These two protein assemblies are composed of four protein subunits, forming heterotetrameric complexes: A_2_B_2_ for DNA gyrase, and C_2_E_2_ for topoisomerase IV. They are well-documented targets for antimicrobial therapy, with the fluoroquinolones being renowned for possessing a “dual-targeting” mechanism with the possibility of inhibiting both enzymes simultaneously. Dual-targeting is an attractive prospect for antimicrobial drug discovery, as inhibition of two enzymes simultaneously presents bacteria with a significant challenge towards resistance evolution.^7,8^

DNA gyrase and topoisomerase IV possess a high degree of sequence and structural similarity, which favours the dual-targeting approach. They possess limited sequence similarity to that of human topoisomerase II which allows for the design of selective inhibitors for these bacterial topoisomerase enzymes over the human topoisomerase II.^9,10^

The more established sites on the topoisomerases for the interaction of inhibitors are associated with their DNA- and ATP-binding sites.^6,11^ In the case of the fluoroquinolones, these drugs bind to the enzyme-DNA complex and effectively “trap” the bound DNA within the enzyme by forming key interactions within the DNA-binding site *via* a water-metal ion bridge.^12^ However, despite the success of dual-targeting agents such as the fluoroquinolones, as well as the relatively slow rate at which bacterial resistance to these drugs has occurred, resistance within the clinic is growing.^13^ There are concerns that eventually, these antibacterial drugs may become ineffective.

Resistance to fluoroquinolones has developed largely due to point mutations which undermine the effectiveness of this antibiotic class. Point mutations within the *gyrA* (*e.g.*, S83 and D87; *Escherichia coli* numbering) and to a lesser degree *gyrB* (*e.g.*, D426 and K447) genes of DNA gyrase, as well as the *parC* (*e.g.*, S80 and E84) gene of topoisomerase IV contribute to fluoroquinolone resistance.^13^ The discovery of potent and novel antimicrobial agents that either bind to alternative regions within the topoisomerases or operate *via* different mechanisms is therefore paramount in combatting the rise of bacterial resistance.

**Fig. 1:**
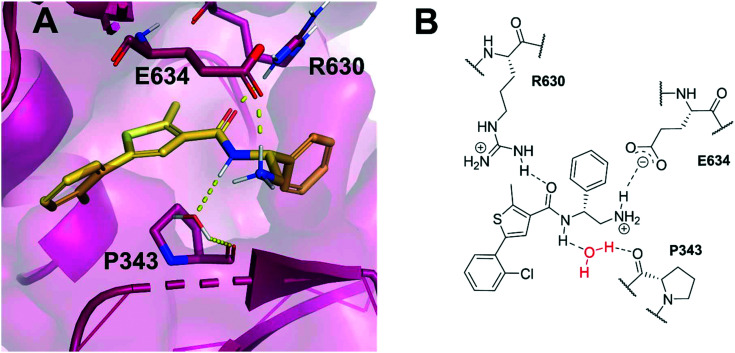
(A) Inhibitor 1 (gold) within the allosteric site of *S. aureus* DNA gyrase (5NPP).^14^ Key polar interactions are shown as yellow dashes to DNA gyrase residues (purple and labelled). Note, the water-mediated hydrogen bond between the amide NH and P343. (B) 2D schematic of panel A, highlighting the conserved water in red.

Allosteric binding sites offer promising, alternative mechanistic types of enzyme inhibition. Chan *et al.* reported one such example within a *Staphylococcus aureus* DNA gyrase structure containing a thiophene-carboxamide inhibitor (1).^14,15^ Inspection of the co-crystal structure revealed the inhibitor to be bound within a pocket between the *GyrA* and *GyrB* subunits, referred to as the hinge region, which is remote from the fluoroquinolone binding site. Inhibitor 1 (IC_50_: 0.3 μM, *E. coli* DNA gyrase) adopts a conformation within this pocket involving the formation of key polar interactions with residues R630 and E634 (*S. aureus* numbering), with the amide carbonyl of the inhibitor acting as a hydrogen bond acceptor for the neighbouring arginine residue, and the terminal amine of the ligand involved in a charge–charge interaction with the neighbouring glutamate residue. It is also observed that an interaction forms between the amide hydrogen bond donor and a water molecule trapped within the X-ray co-crystal structure, forming a water-mediated hydrogen bond to P343 (Fig. 1). The compound inhibits the supercoiling ability of DNA gyrase, as well as stabilising gyrase-dependent DNA cleavage *via* a mixture of double- and single-strand cleavage, but does not inhibit topoisomerase IV (IC_50_: >540 μM), questioning whether dual-targeting can be achieved within this allosteric site.^14^ Ultimately, the development of inhibitor 1 was terminated due to observed *in vivo* toxicity issues, although a later publication by the same group described further examples of fused heterocycles replacing or incorporating the thiophene.^15^

The principles of structure-based ligand design were introduced in the 1980s.^16^ Following significant developments in technological processing power, *in silico* software, and the availability of high-resolution crystal structures, alternative computational methods to high-throughput screening (HTS) became commonplace to investigate protein target sites and initiate drug discovery projects. SPROUT^17^ is a program used for *de novo* structure-based molecular design, using a fragment-based approach to design novel scaffolds that can then be ranked by predicted binding affinity. It has been used to design inhibitors of several enzymes derived from pathogens.^18–22^ Using the co-crystal structure of *S. aureus* DNA gyrase with thiophene inhibitor 1 (PDB ID: 5NPP),^14^ we aimed to design novel compounds that bind within the allosteric site and may address the threat of fluoroquinolone resistance by retaining gyrase activity against fluoroquinolone-resistant strains and simultaneously possessing activity against topoisomerase IV.

## Results and discussion

**Fig. 2:**
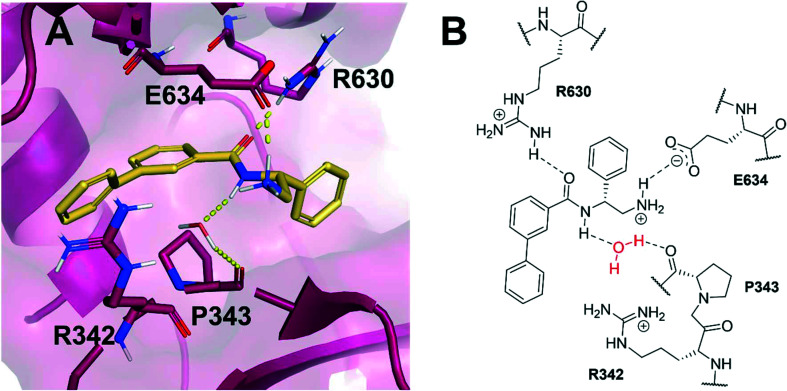
(A) Novel biphenyl inhibitor design 2 (gold) modelled within the allosteric site of *S. aureus* DNA gyrase (5NPP).^14^ Key polar interactions shown in yellow and key residues (purple) are labelled. (B) 2D schematic of panel A in the same orientation as Fig. 1B.

Based on the X-ray conformation of compound 1 from 5NPP, we decided to utilise the three key polar interactions in our molecular designs (R630, E634 and the water-mediated bond to P343). This allowed the ‘right-hand’ portion of inhibitor 1 (as illustrated in Fig. 1) to be kept consistent within the SPROUT software, and the hydrophobic left-hand portion to be varied by the steric constraints of the binding cavity. These constraints vary between gyrase and topo IV, but the sequence conservation of the allosteric site is high amongst pathogenic bacterial species and topoisomerase enzymes, validating 5NPP as a model system.^14^ Five- and six-membered aromatic rings and single carbon atoms were selected in SPROUT to produce an array of template skeletons which could be fused to the ‘right-hand’ portion of 1 (see ESI† for further details and Fig. S1). Amongst the initial results, a biphenyl molecular scaffold, as represented by biphenyl inhibitor 2, was a synthetically attractive candidate to test the potential of our *de novo* design approach to targeting this binding site. It was proposed that compound 2 would retain the same polar interactions as observed for thiophene inhibitor 1, with subsequent analogues targeting the guanidine side chain of R342 *via* appropriate ring substituents (Fig. 2). R342 is ∼3.5 Å from the terminal phenyl ring but the angle between the guanidine group and the aryl ring is not favourable for a cation–π interaction within the SPROUT pose of 2 nor the crystal structure of 1.

Biphenyl inhibitor 2 was synthesised and tested in an *in vitro E. coli* DNA gyrase supercoiling assay, revealing it to be a moderate inhibitor of *E. coli* gyrase (IC_50_: 60 μM, chemical synthesis and biochemical assays described below). With this encouraging result in mind, we endeavoured to explore the structure–activity relationship (SAR) by varying the substitution pattern on the biphenyl unit.

To help guide the design of these substituted analogues, the docking module Glide^23^ within the Schrödinger Maestro software package^24^ was used. Various functional groups were computationally added to the biphenyl ring system, and these compounds were then docked in standard precision (SP) mode within the allosteric site of the 5NPP co-crystal structure to explore the steric and electronic characteristics within the hydrophobic cavity. Most compounds were predicted to display enhanced binding to *S. aureus* gyrase through slightly improved docking scores compared with 2 (an empirical measure of predicted binding affinity, see Table S2† for full list of docking scores). Generally, substituents at the *meta*-position on the terminal phenyl ring had improved docking scores over the *ortho*-position. This was rationalised on two accounts: (i) favourable van der Waals interactions due to an induced dihedral twist of the biphenyl system due to the size and position of the substituent, and (ii) a potential interaction between the substituent at the *meta*-position and R342.

A corresponding ring twist (43°) was observed in thiophene 1 within the 5NPP structure, a likely determinant for the biologically-active conformation (Fig. 1). Most substituents were predicted to induce a similar twist in the biphenyl structure, by restricting free rotation of the carbon–carbon single bond. There would be a lower barrier to rotation in biphenyl 2, although a dihedral angle of 61° was measured in the docked conformation of 2 (Fig. 2).

To explore the docking predictions and establish the SAR, a series of substituted compounds containing electron-donating substituents (3, 4 and 8), electron-withdrawing substituents (5, 11, 12, 13 and 14), halogen atoms (9 and 10) and combinations of electronics (6 and 7) were prepared (Table 1). The smaller *des*-phenyl, bromo intermediate (15) was also tested to explore the hydrophobic binding requirements of the allosteric site.

**Table 1:** *In vitro* data for the novel biphenyl series of DNA gyrase inhibitors designed to investigate the SAR and hydrophobic and steric requirements of the *E. coli* DNA gyrase allosteric site

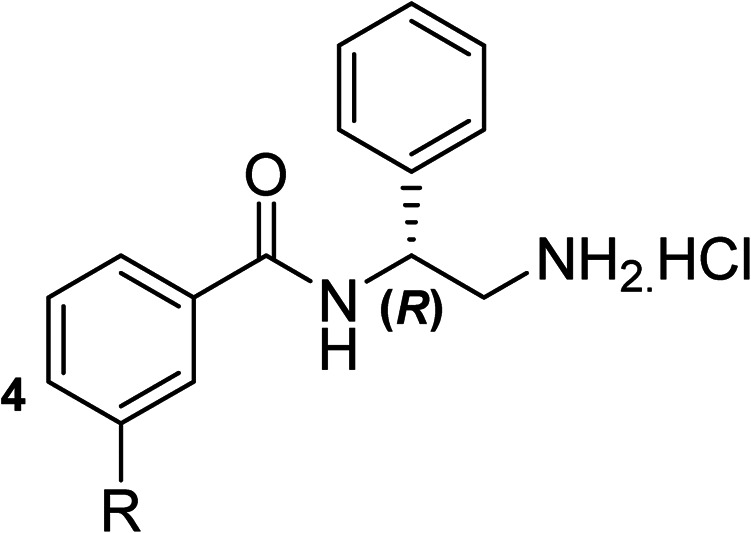					
Compound	*R*	IC_50_^a^ (μM)	Compound	*R*	IC_50_^a^ (μM)
2	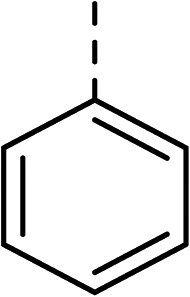	60	9	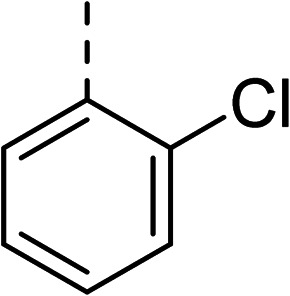	35
3	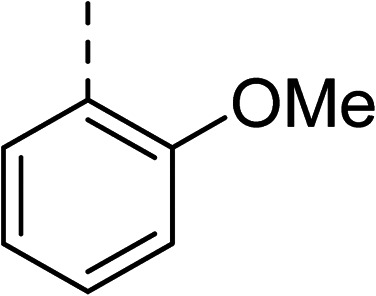	20	10	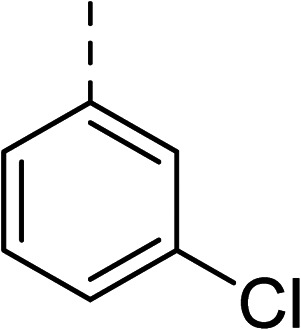	39
4	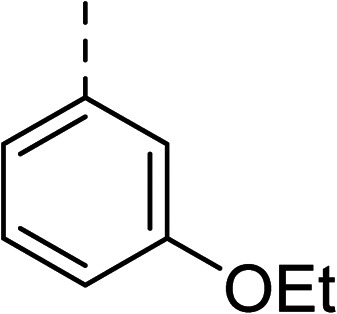	20	11	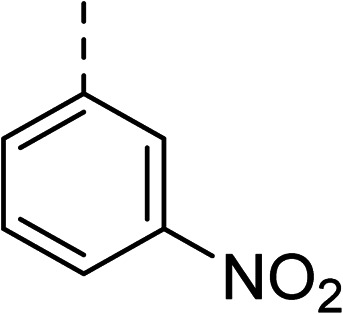	73
5	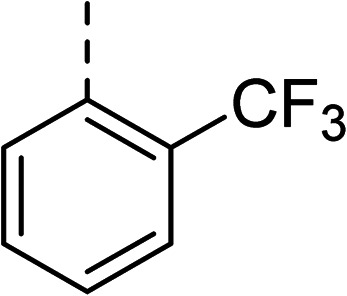	21	12	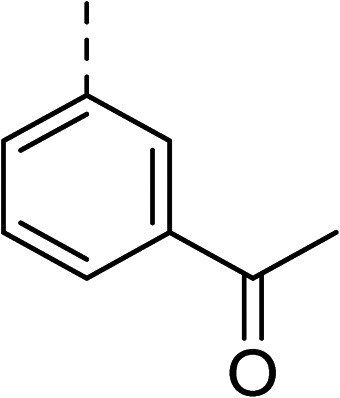	42
6	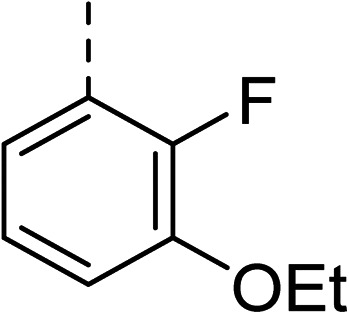	12	13	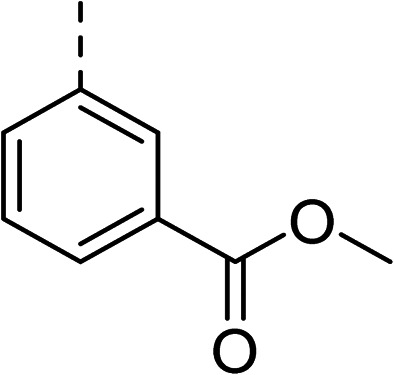	24
7	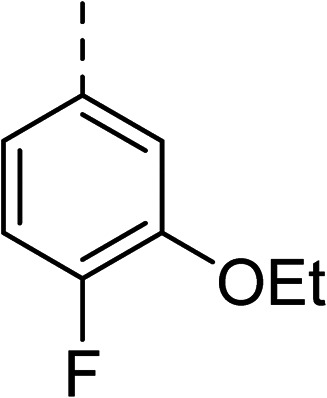	17	14	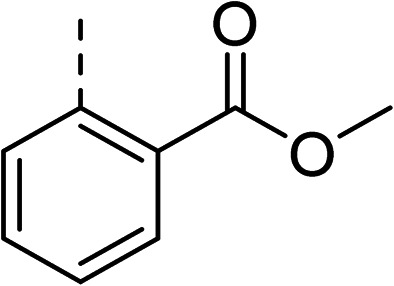	41
8	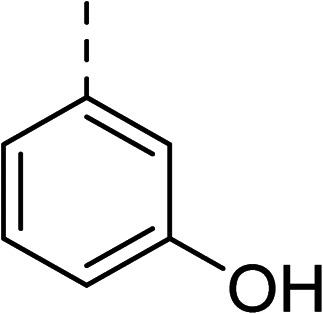	46	15	Br	>200

a IC_50_ values were the mean of 2 or 3 different experiments. Error limits are not given, as the gel-based assays used are at best semi-quantitative.

**Scheme 1:**
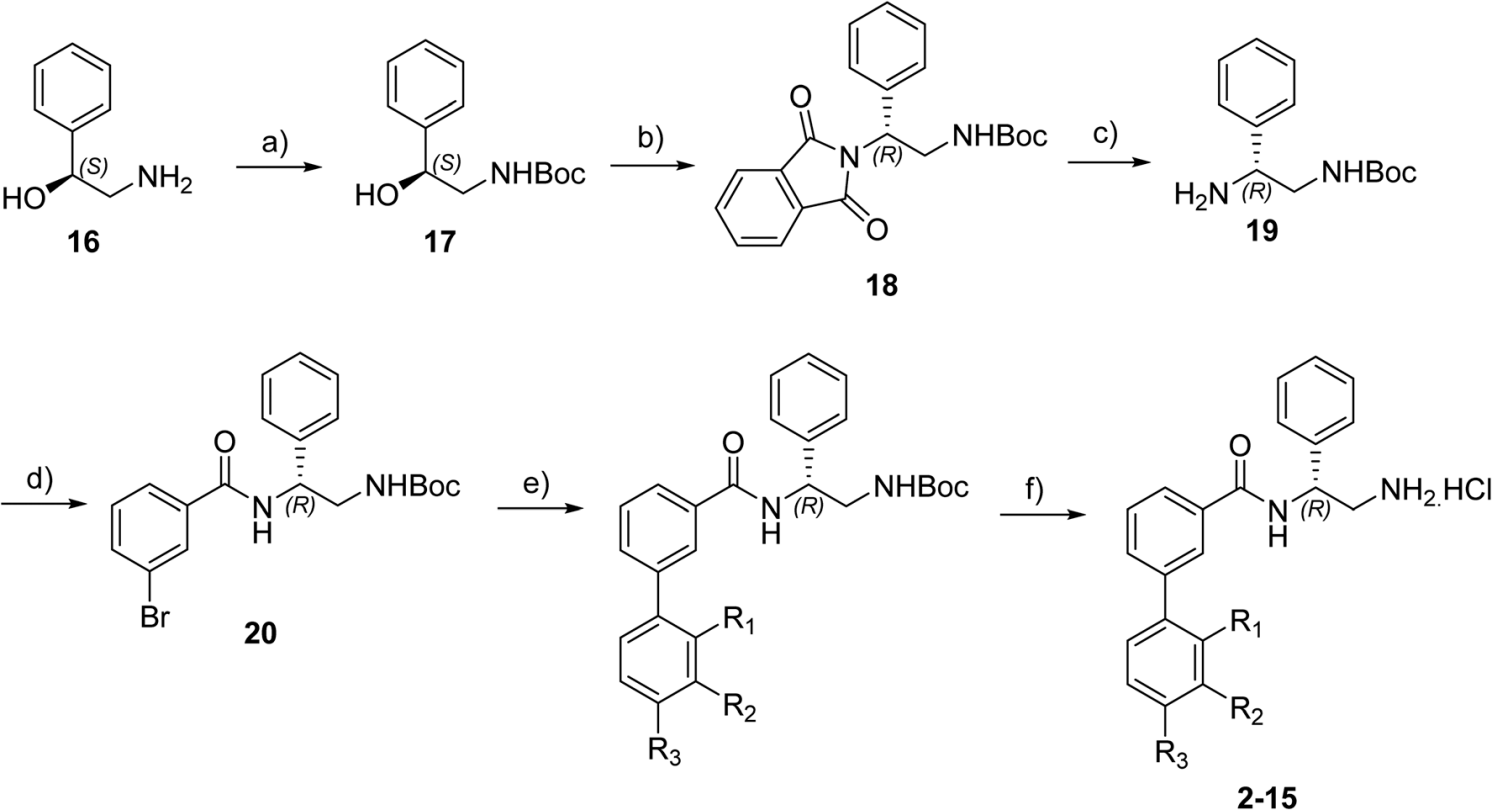
Reagents and conditions: a) Boc_2_O, THF, RT, 1 hour, 89%. b) Phthalimide, PPh_3_, DEAD, THF, N_2_, 0 °C – RT, 18 hours, 63%. c) N_2_H_4_·H_2_O, EtOH, 60 °C, 4 hours, 74%. d) 3-Bromobenzoyl chloride, NEt_3_, DCM, N_2_, RT, 22 hours, 77%. e) Substituted boronic acid, Pd(PPh_3_)_4_, propanol, 2 M Na_2_CO_3_ aqueous solution, N_2_, reflux, 20–35%. f) 4 N HCl in dioxane, RT, 65–93%.

The synthesis of these compounds was readily achieved using protocols adapted from Chan *et al.* (Scheme 1).^14^ The commercially available (*S*)-2-amino-1-phenylethanol starting material (16) underwent Boc-protection to result in 17 in excellent yield. Intermediate 17 was used in a Mitsunobu reaction to switch the alcohol for a phthalimide functionality, leading to inversion of stereochemistry, giving intermediate 18 in good yield. This was subsequently followed by cleavage of the phthalimide unit using hydrazine (Gabriel synthesis) to give the chiral primary amine 19 in good yield. Amine 19 was then coupled to 3-bromobenzoyl chloride to form the key amide linker, with the 3-bromo position of 20 primed for the ensuing Suzuki–Miyaura coupling of various substituted boronic acids. The Suzuki–Miyaura couplings proved facile but proceeded in relatively poor yields. The Boc-protecting group was then removed using strong acid (4 N HCl in dioxane) to give the final biphenyl inhibitors 2–15 in excellent yields.

The compounds were tested in a DNA supercoiling assay with *E. coli* DNA gyrase using gel electrophoresis. This is a semi-quantitative assay which is used to identify potent compounds relative to a positive control (usually ciprofloxacin, IC_50_: 0.6 μM, *E. coli* gyrase^14^) and establish the SARs within a compound series. Compounds were tested in duplicate (*n* = 2) or triplicate (*n* = 3).

Pleasingly, all compounds except 11 and 15 showed enhanced inhibitory potency *vs.* the unsubstituted system (2, IC_50_: 60 μM), with inhibitor 6 displaying a 5-fold increase in binding affinity against gyrase (IC_50_: 12 μM). Analysis of the biological data in Table 1 revealed that derivatisation of the biphenyl terminal ring is generally tolerated. There was no significant difference in activity between compounds with similar substituents at the *ortho*- or *meta*-positions (*e.g.*, matched pair 9 and 10, or 3 and 4), suggesting that the docking scores do not correlate with the observed biological activity. There was also no significant difference between compounds containing electron-donating substituents (3, 4, 6, 7, and 8) or electron-withdrawing substituents (5, 9, 10, 12–14), except compound 11 which contained a 3-nitro group and displayed lower activity (IC_50_: 73 μM) compared to unsubstituted biphenyl 2. Notably, when the terminal phenyl ring was replaced with a bromine atom (15) activity dropped at least 3-fold compared to 2. Compound 15 is predicted to retain the three key polar interactions observed within the biphenyl series and thiophene 1, but has significantly weaker activity (IC_50_: >200 μM). This alludes to the importance of optimising the aryl interactions in this area of the allosteric pocket.

With a broad SAR profile in hand, further analogues were synthesised that would probe the hydrophobic and steric requirements of the hydrophobic cavity in DNA gyrase, investigate the chirality at the asymmetric centre and explore the addition of substituents to the chiral phenyl ring. The methyl and chloro substituents on thiophene inhibitor 1 appeared to optimally fill small cavities within hydrophobic pocket.^14^ We explored a similar strategy on our biphenyl series by systematically introducing methyl groups on the benzamide ring (21–23, Table 2). Whilst these compounds lacked the terminal phenyl ring, we thought that by optimally filling the pocket we would still observe gyrase inhibition. Compounds 21–23 displayed weak activity (IC_50_: >100 μM) but inspection of the gels suggested that 23 was superior to 21 and 22 (Fig. S2†). Based on these results, compounds 24–26 were synthesised using the synthetic steps outlined in Scheme 1 which contained a methyl group at the 4-position on the benzamide ring and a substituted terminal phenyl respectively (Table 2). Unfortunately 24–26 were not superior to the most active compounds in Table 1 suggesting that the 4-methyl group on the benzamide ring does not fill a hydrophobic pocket and that other vectors should be pursued. The pyridine-containing 27 was synthesised (Scheme S1†) to explore whether more polar ring systems were tolerated in this hydrophobic pocket. Compound 27 displayed similar potency (IC_50_: 35 μM) to phenyl analogue 9, suggesting a further avenue to explore in future.

**Table 2:** *In vitro* biological results for the compounds exploring the hydrophobic pockets of the allosteric site. IC_50_ values determined against *E. coli* DNA gyrase

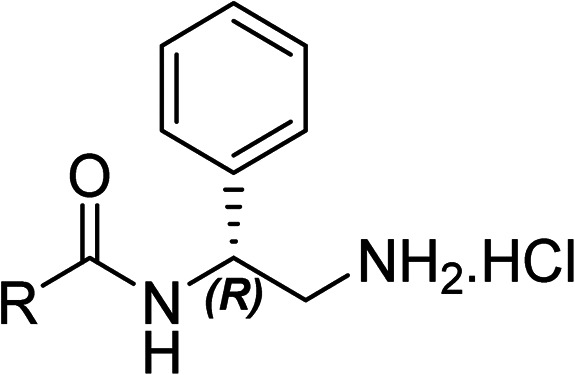					
Compound	*R*	IC_50_ (μM)	Compound	*R*	IC_50_ (μM)
21	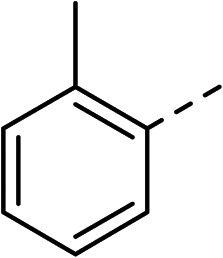	>100	24	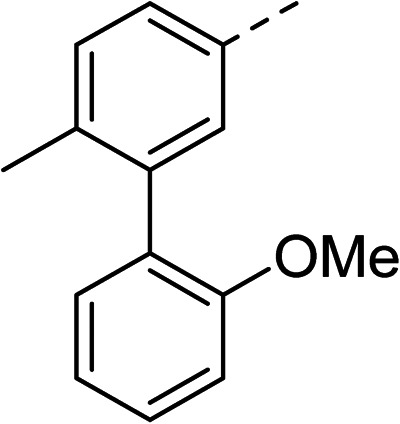	153
22	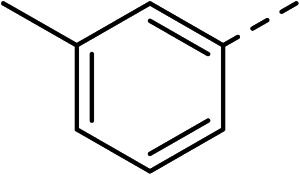	>100	25	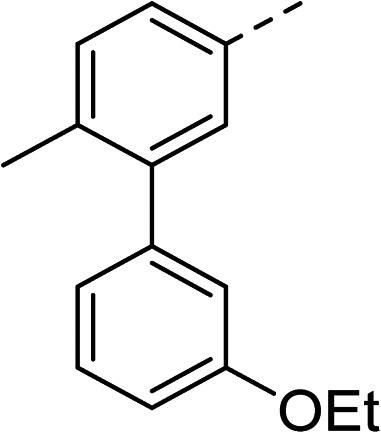	63
23	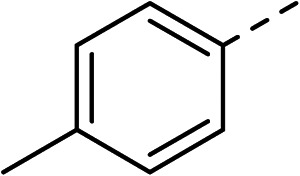	>100	26	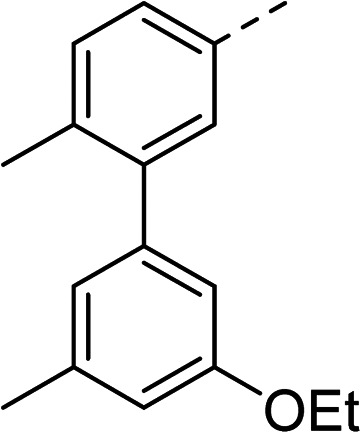	56
			27	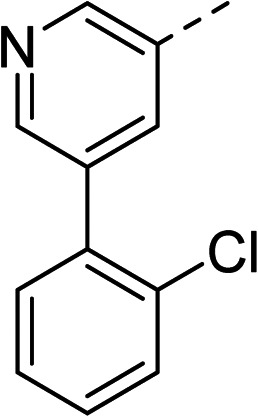	35

The (*R*)-stereochemistry present in thiophene 1 was required for binding to the allosteric site by positioning the chiral phenyl moiety in a favourable vector. The (*S*)-enantiomer of 1 was reported as being ∼30-fold less active.^14^ In order to ascertain whether the (*R*)-stereochemistry was crucial for our biphenyl inhibitor series, a number of (*S*)-enantiomer compounds (28–30) were prepared using an analogous sequence to that shown in Scheme 1 but beginning from (*R*)-2-amino-1-phenylethanol (Table 3). These (*S*)-enantiomers showed a general decrease in activity compared to their enantiomeric (*R*)-counterparts, although the trends in substitution at the *R*_1_ position were mirrored: 28 (IC_50_: 76 μM) *vs.*2 (IC_50_: 60 μM) and 29 (IC_50_: 36 μM) *vs.*3 (IC_50_: 20 μM). A more significant drop in activity (9-fold) for the (*S*)-enantiomer 30 (IC_50_: 180 μM) was observed *vs.* the (*R*)-henantiomer 4 (IC_50_: 20 μM) when *R*_1_ was 3-ethoxyphenyl. At present, we have no explanation for this observation as retrospective docking using Glide suggested that compounds 28–30 had similar docking scores (Table S3†) and docked conformations (Fig. S3†).

**Table 3:** Further SAR exploring the role of chirality and substitution on the phenyl ring. IC_50_ values were determined against *E. coli* DNA gyrase in a supercoiling assay

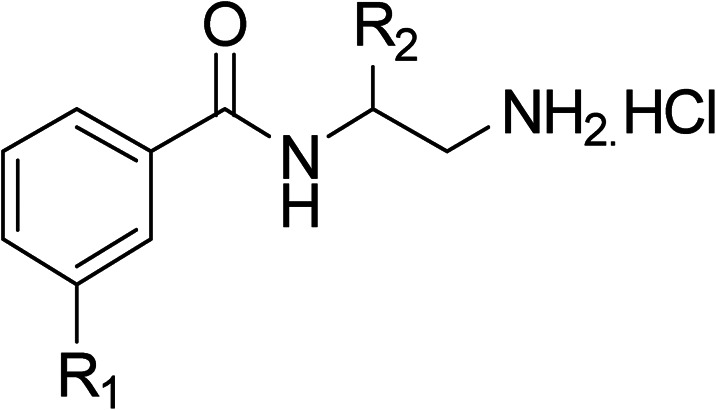			
Compound	*R*_1_	*R*_2_	IC_50_^a^ (μM)
28	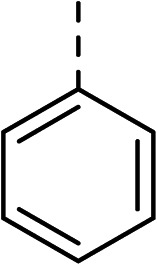	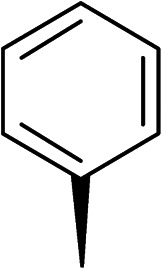	76
29	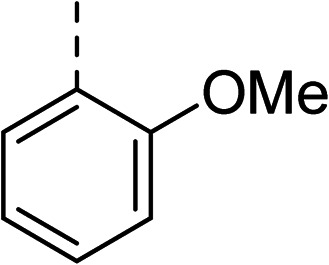	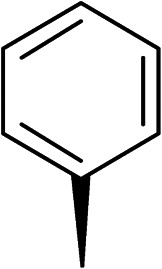	36
30	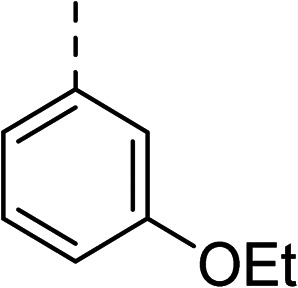	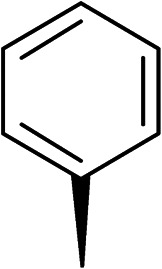	180
31	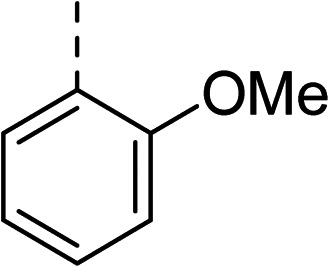	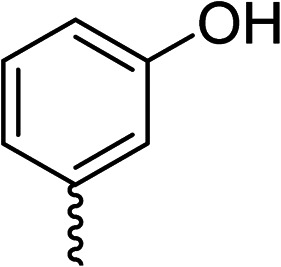	41
32		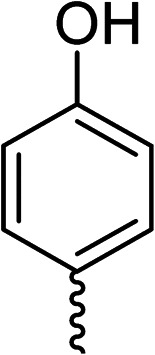	45
33	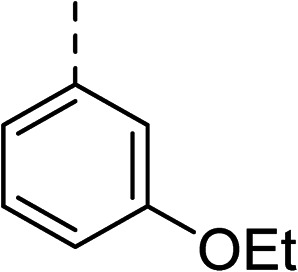	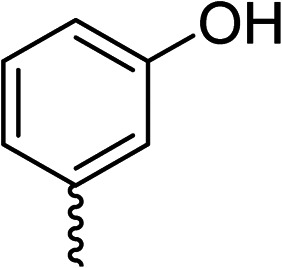	41
34	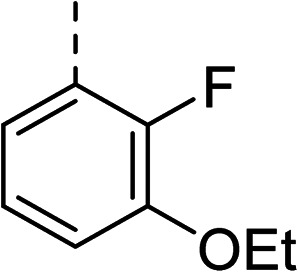	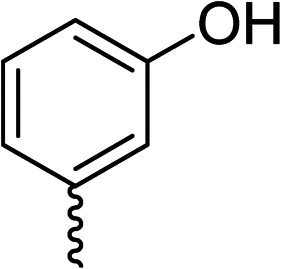	62
35		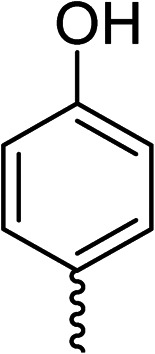	>200

a IC_50_ values were the mean of 2 or 3 different experiments. Error limits are not given, as the gel-based assays used are at best semi-quantitative.

Whilst exploring the chirality of the phenyl group, we also synthesised some phenol analogues. This ring system was selected due to favourable molecular modelling predictions which suggested that this solvent-exposed region of the pocket could potentially offer an additional bonding interaction to R630 *via* an intermolecular bridge with a labile water molecule. For these phenol analogues, an optically pure starting material was not commercially available, and so racemic starting materials were used during their synthesis (see Scheme S2† for adapted synthetic route). Five analogues (31–35) were synthesised and tested *in vitro*, with all analogues except 35 being moderate inhibitors of DNA gyrase (Table 3), suggesting no additional interaction had formed.

Finally, several biphenyl inhibitors underwent further evaluation *in vitro* against *E. coli* topoisomerase IV and *E. coli* K12 MG1655 and *S. aureus* NCIMB 50080 bacterial cells, for which IC_50_ and MIC values were determined respectively (Table 4). DNA relaxation assay with *E. coli* topo IV is a semi-quantitative assay used to identify potent compounds relative to a positive control (novobiocin, IC_50_: 11 μM, *E. coli* topo IV). Compounds were tested in duplicate (*n* = 2) or triplicate (*n* = 3). Additionally, compounds were evaluated against fluoroquinolone mutant strain *E. coli* K12 MG1655 S83L to confirm mode of action.

**Table 4:** Biphenyl inhibitors with further biological evaluation reported. IC_50_ values were the mean of 2 or 3 different experiments. Error limits are not given, as the gel-based assays used are at best semi-quantitative

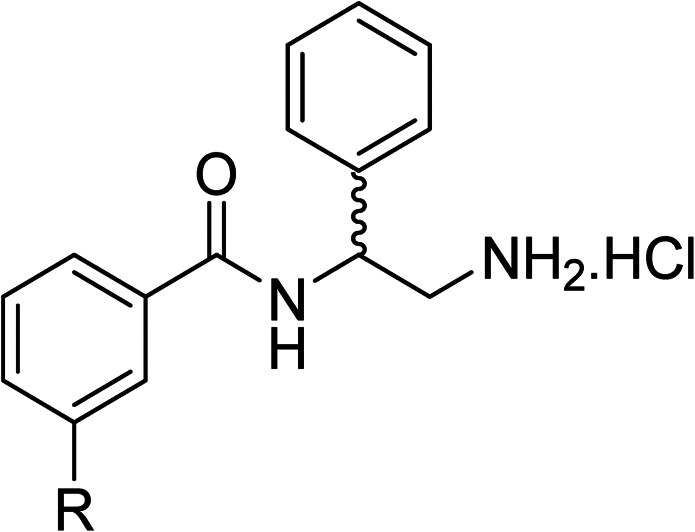							
Compound	*R*	Ring chirality	*E. coli* DNA gyrase IC_50_ (μM)	*E. coli* topo IV IC_50_ (μM)	*E. coli* K12 MG1655 MIC (μg mL^−1^)	*S. aureus* NCIMB 50080 MIC (μg mL^−1^)	*E. coli* K12 MG1655 S83L MIC (μg mL^−1^)
Ciprofloxacin^b^			0.6	5.7	0.016	0.5	0.5
1	Thiophene		0.3^a^	>540^a^	8^a^	8^a^	N.D.
5	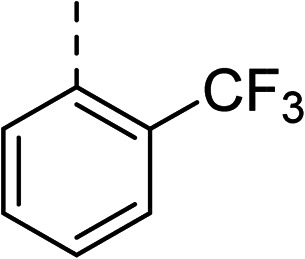	(*R*)	21	>100	32	16	32
6	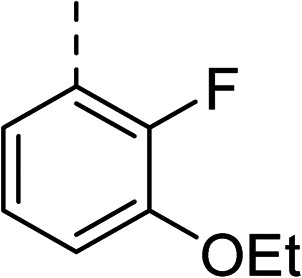	(*R*)	12	>100	64	32	64
7	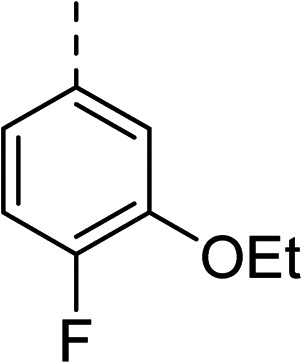	(*R*)	17	64	32	16	32
11	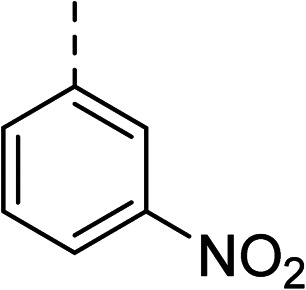	(*R*)	73	94	>128	>128	>128
31	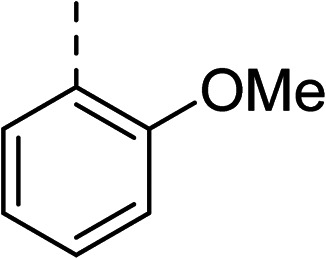	Racemic (3-OH)	41	>100	>128	128	>128

a Data from ref. 14 which used different *E. coli* and *S. aureus* bacterial strains.

b Ciprofloxacin used as a standard control.

Topoisomerase IV inhibition was observed for compounds 7 and 11 but not compounds 5 and 6. This was surprising given that compounds 6 and 7 are geometrical isomers and suggests that the *para*-position of the terminal phenyl ring should be further derivatised. Notably, 7 displays significantly more *E. coli* topo IV inhibition than that of thiophene inhibitor 1, although DNA gyrase inhibition remains lower (Table 4). We believe that compound 7 has the most promising topo IV inhibition of any gyrase allosteric inhibitor to date, outperforming the most active topo IV inhibitor (topo IV IC_50_: ∼90 μM) from the work of Thalji *et al.*^15^ While compound 7 is ∼4-fold more potent against gyrase than topo IV, it demonstrates micromolar activity against both enzymes and holds great potential for the dual-targeting ability of the allosteric site, as our allosteric inhibitors start to mirror the activity trend of the fluoroquinolone antibiotics (ciprofloxacin, gyrase IC_50_: 0.6 μM, topo IV IC_50_: 5.7 μM).^14^

Biphenyl inhibitors 5–7 were also found to possess some Gram-positive cellular activity *vs. S. aureus* (MIC: 16–32 μg mL^−1^) and weak Gram-negative activity *vs. E. coli* (MIC: 32–64 μg mL^−1^). Furthermore, there was no change in the MIC values for 5–7 against the fluoroquinolone *E. coli* mutant S83L, confirming that these compounds do not bind to the fluoroquinolone binding site. This compound series largely adheres to the eNTRy rules for accumulation into Gram-negative bacteria (*e.g.*, the presence of a primary, ionisable nitrogen and overall low globularity, but with some analogues there are >5 rotatable bonds which negatively affects flexibility in relation to the eNTRy rules), but further studies are required to determine if these compounds are subjected to efflux mechanisms.^25^

## Conclusions

In summary, the use of a *de novo* molecular design approach has successfully led to the identification of a new series of biphenyl-based inhibitors targeting a novel allosteric binding site within bacterial DNA gyrase. Numerous compounds were synthesised based on molecular modelling hypotheses and subsequently tested *in vitro* against DNA gyrase using a supercoiling assay. This led to the identification of biphenyl inhibitor 7 which displayed moderate potency against *E. coli* gyrase (IC_50_: 17 μM) and *E. coli* topo IV (IC_50_: 64 μM), and antibacterial activity against both Gram-positive and -negative bacteria.

Further work is underway to improve the gyrase, topo IV and antibacterial activity of the biphenyl series, as well as the development of alternative SPROUT scaffolds for this allosteric site. In particular, our focus is on the dual gyrase and topo IV target potential of the allosteric site as bacterial resistance has yet to evolve here. Our approach demonstrates that a combination of traditional medicinal chemistry and *in silico* molecular modelling can identify new scaffolds for this target and may lay the foundations to identify future novel gyrase and topo IV antibacterial agents.^26^

## Author contributions

MJM and KMO designed the compounds. KMO, JFN, JNS, SG, BKLB and HLJ synthesised the compounds. LF, TG and LRA evaluated the compounds. MJM, AM and CWGF designed the study. KMO, MJM, LF, AM and CWGF wrote the manuscript. All authors contributed to the editing of the manuscript.

## Conflicts of interest

There are no conflicts to declare.

## Supplementary Material

MD-013-D2MD00049K-s001

## References

[cit1] O'Neill J. , The Review On Antimicrobial Resistance, 2016

[cit2] Antimicrobial Resistance Collaborators (2022). Lancet.

[cit3] Bush N. G., Evans-Roberts K., Maxwell A. (2015). EcoSal Plus.

[cit4] McKie S. J., Neuman K. C., Maxwell A. (2021). BioEssays.

[cit5] Hooper D. C., Jacoby G. A. (2016). Cold Spring Harbor Perspect. Med..

[cit6] Bush N. G., Diez-Santos I., Abbott L. R., Maxwell A. (2020). Molecules.

[cit7] Tse-Dinh Y. C. (2016). Future Med. Chem..

[cit8] Pan X. S., Fisher L. M. (1998). Antimicrob. Agents Chemother..

[cit9] Tsai-Pflugelder M., Liu L. F., Liu A. A., Tewey K. M., Whang-Peng J., Knutsen T., Huebner K., Croce C. M., Wang J. C. (1988). Proc. Natl. Acad. Sci. U. S. A..

[cit10] Wyckoff E., Natalie D., Nolan J. M., Lee M., Hsieh T. (1989). J. Mol. Biol..

[cit11] Maxwell A. , Bush N. G., Germe T., McKie S. J., Fong I. W., Shlaes D. and Drlica K., Antimicrobial resistance and implications for the 21st century, Springer, Switzerland, 2018

[cit12] Aldred K. J., McPherson S. A., Turnbough Jr. C. L., Kerns R. J., Osheroff N. (2013). Nucleic Acids Res..

[cit13] Mitscher L. A. (2005). Chem. Rev..

[cit14] Chan P. F., Germe T., Bax B. D., Huang J., Thalji R. K., Bacqué E., Checchia A., Chen D., Cui H., Ding X., Ingraham K., McCloskey L., Raha K., Srikannathasan V., Maxwell A., Stavenger R. A. (2017). Proc. Natl. Acad. Sci. U. S. A..

[cit15] Thalji R. K., Raha K., Andreotti D., Checchia A., Cui H., Meneghelli G., Profeta R., Tonelli F., Tommasi S., Bakshi T., Donovan B. T., Howells A., Jain S., Nixon C., Quinque G., McCloskey L., Bax B. D., Neu M., Chan P. F., Stavenger R. A. (2019). Bioorg. Med. Chem. Lett..

[cit16] Mauser H., Guba W. (2008). Curr. Opin. Drug Discovery Dev..

[cit17] Gillet V., Johnson A. P., Mata P., Sike S., Williams P. (1993). J. Comput.-Aided Mol. Des..

[cit18] McPhillie M. J., Trowbridge R., Mariner K. R., O'Neill A. J., Johnson A. P., Chopra I., Fishwick C. W. G. (2011). ACS Med. Chem. Lett..

[cit19] Cowen D., Bedingfield P., McConkey G. A., Fishwick C. W. G., Johnson A. P. (2010). Bioorg. Med. Chem. Lett..

[cit20] Cain R., Brem J., Zollman D., McDonough M. A., Johnson R. M., Spencer J., Makena A., Abboud M. I., Cahill S., Lee S. Y., McHugh P. J., Schofield C. J., Fishwick C. W. G. (2018). J. Med. Chem..

[cit21] Narramore S., Stevenson C. E. M., Maxwell A., Lawson D. M., Fishwick C. W. G. (2019). Bioorg. Med. Chem..

[cit22] Yule I. A., Czaplewski L. G., Pommier S., Davies D. T., Narramore S. K., Fishwick C. W. G. (2014). Eur. J. Med. Chem..

[cit23] Friesner R. A., Banks J. L., Murphy R. B., Halgren T. A., Klicic J. J., Mainz D. T., Repasky M. P., Knoll E. H., Shaw D. E., Shelley M., Perry J. K., Francis P., Shenkin P. S. (2004). J. Med. Chem..

[cit24] Maestro, Schrödinger, LLC, New York, NY, 2021

[cit25] Muñoz K. A., Hergenrother P. J. (2021). Acc. Chem. Res..

[cit26] Orritt K. M., Maxwell A., Fishwick C. W. G., McPhillie M. J. (2021). Future Med. Chem..

