# Supplementary material for: De novo design of type II topoisomerase inhibitors as potential antimicrobial agents targeting a novel binding region
Source: RSC Med Chem. 2022 Jun 16;13(7):831–9. doi: 10.1039/d2md00049k (PMC9298182; doi:10.1039/d2md00049k)
Supplement: MD-013-D2MD00049K-s001 [file MD-013-D2MD00049K-s001.pdf]

Electronic Supplementary Material (ESI) for RSC Medicinal Chemistry.
This journal is © The Royal Society of Chemistry 2022

Supporting Information

De Novo Design of Type II Topoisomerase Inhibitors as Potential Antimicrobial Agents Targeting a Novel Binding Region

Kyle M. Orritt,[a] Lipeng Feng,[b] Juliette F. Newell,[a] Jack N. Sutton,[a] Scott Grossman,[a] Thomas Germe,[b,1] Lauren R. Abbott,[b,2] Holly L. Jackson,[a] Benjamin K. L. Bury,[a,3] Anthony Maxwell,[b]* Martin J. McPhillie,[a]* Colin W. G. Fishwick[a]*

[a] School of Chemistry, University of Leeds, Leeds, LS2 9JT, United Kingdom

[b] Dept. Biochemistry & Metabolism, John Innes Centre, Norwich Research Park, Norwich, NR4 7UH, United Kingdom

[1] Current address: Dept. of Biochemistry and Molecular Genetics, University of Colorado Anschutz, 12801 E 17th Ave, CO 80045, USA.

[2] Current address: Dept. of Molecular and Cell Biology, University of Leicester, Leicester, LE1 7RH, United Kingdom

[3] Current address: Charles River Early Discovery, 8-9 The Spire Green Centre, Harlow, CM19 5TR

*Corresponding authors: Tony.Maxwell@jic.ac.uk, M.J.McPhillie@leeds.ac.uk, C.W.G.Fishwick@leeds.ac.uk

Contents

General Information and Instrumentation 3

E. coli DNA Gyrase Supercoiling Inhibition Assay..4

E. coli Topoisomerase IV Relaxation Inhibition Assay 5

Determination of Minimum Inhibitory Concentration (MIC)..6

Docking Protocol with Schrödinger...7

Supplementary Data 8

SPROUT *de novo* design run 8

Docking Scores of biphenyl analogues 10

Methyl Variants 11

Comparison of biphenyl enantiomers 11

Synthesis of Pyridine-containing Compound **27** 13

Alternative Synthesis to Racemic Phenol Variants 14

Reported Mass Spectrometry Data...15

Compound Numbering for NMR Assignment 16

General Experimental Procedures 17

Method A: Suzuki Couplings...17

Method B: TIPS-Deprotection 17

Method C: Boc-Deprotection...17

Preparation of Compounds..18

References 66

General Information and Instrumentation

All solvents and reagents were obtained from commercial suppliers and used without further purification. Solvents used were HPLC or analytical grade. Thin layer chromatography was performed on aluminium backed silica gel supplied by Merck, visualised using an ultraviolet lamp. Flash column chromatography was performed using silica gel 60 (40-63 μm particles). Automated flash column chromatography was performed on a Biotage® Isolera™ One machine using Biotage® Sfär columns of varying sizes between 5 g and 100 g. Automated reverse phase flash column chromatography was performed using C18 silica columns. Microwave syntheses were performed using an Anton Parr Monowave 50 reactor. Hydrogen and carbon NMR data were collected on a Bruker Avance III 500. All shifts were recorded against an internal standard of tetramethyl silane. Solvents used for NMR (chloroform-*d*, methanol-d_4 and DMSO-d_6) were obtained from Sigma-Aldrich. ^{1}H NMR data is reported in the following format: ppm (splitting pattern, coupling constant (Hz), number of protons, proton assignment). Signal assignments were deduced with the aid of TopSpin, MestReNova, DEPT 135, COSY, HSQC and HMBC. LC-MS (liquid chromatography-mass spectrometry) data were recorded on a Donex Ultimate 3000 LC system with a MeCN/H_2O +0.1% formic acid gradient. HR-MS data were recorded using a Bruker MaXis impact spectrometer using electron spray ionisation. Infrared spectra were recorded on a Perkin-Elmer one FTIR spectrometer. Melting points were recorded on Griffin Education MELTP melting point apparatus.

E. coli DNA Gyrase Supercoiling Inhibition Assay

On ice, a master mix of H_2O, dilution buffer, 5X assay buffer and relaxed plasmid DNA (pBR322) was prepared and divided into aliquots (27.5 µL)*. The reaction mixtures were then combined with the relevant compound in DMSO (0.5 µL). Gyrase (2 µL) was then pipetted onto the side of each sample tube and briefly centrifuged to allow for simultaneous mixing, before incubation at 37°C for 30 mins. The reaction was stopped by addition of STEB (20 µL) and chloroform:isoamyl alcohol (24:1; 30 µL) with gentle vortexing. To isolate the DNA product, the samples were then centrifuged for 2 mins at 13,000 rpm. The aqueous (blue) layer of each sample was extracted and loaded onto a 1% (w/v) agarose gel and 80 V applied over 2 hrs (or 15 V overnight).

*To achieve equal sample volumes, the quantity of compound added was supplemented with H_2O to total 17.5 µL and the volume of gyrase enzyme was balanced with dilution buffer to total 6 µL. Volumes of 5X assay buffer and pBR322 remained constant; 6 µL and 0.5 µL, respectively.

Gels were then stained in 1 µg/mL of EtBr in H_2O for 20 mins and de-stained in H_2O for 5 mins, before imaging with a UV gel documentation system (Syngene). The supercoiling extent was quantified using ImageJ software and IC_{50} values obtained from a derived form of the Hill equation.

An appropriate enzyme concentration was determined by observing supercoiling extent with a range of gyrase concentrations against a constant volume of substrate (linear pBR322). The gyrase level that produced a midpoint quantity of supercoiling was chosen.

Storage of reagents: neat gyrase subunits should be stored at -80°C and dilutions made fresh. Buffers, DNA and compound dilutions should be stored at -20°C or below.

E. coli gyrase assay buffer (1X recipe): Tris·HCl pH 7.5 (35 mM), KCl (24 mM), $MgCl_2$ (4 mM), DTT (2 mM), spermidine (1.8 mM), ATP (1 mM), 6.5 % (w/v) glycerol and albumin (0.1 mg/mL).

E. coli gyrase dilution buffer: Tris·HCl pH 7.5 (50 mM), KCl (100 mM), DTT (2 mM), EDTA (1 mM) and 50% (w/v) glycerol.

STEB: Sucrose 40% (w/v), Tris-HCl pH 8.0 (100 mM), EDTA (100 mM), Bromophenol Blue (0.5 mg/mL).

E. coli Topoisomerase IV Relaxation Inhibition Assay

On ice, a master mix of H_2O, dilution buffer, 5X assay buffer and supercoiled plasmid DNA (pBR322) was prepared and divided into aliquots (26.5 µL)*. The reaction mixtures were then combined with the relevant compound in DMSO (0.5 µL). Topoisomerase IV (3 µL) was then pipetted onto the side of each sample tube and briefly centrifuged to allow for simultaneous mixing, before incubation at 37°C for 30 mins. The reaction was stopped by addition of STEB (20 µL) and chloroform:isoamyl alcohol (24:1; 30 µL) with gentle vortexing. To isolate the DNA product, the samples were then centrifuged for 2 mins at 13,000 rpm. The aqueous (blue) layer of each sample was extracted and loaded onto a 1% (w/v) agarose gel and 16 V applied overnight.

*To achieve equal sample volumes, the quantity of compound added was supplemented with H_2O to total 17.8 µL and the volume of topo IV enzyme was balanced with dilution buffer to total 6 µL. Volumes of 5X assay buffer and pBR322 remained constant; 6 µL and 0.3 µL, respectively.

Gels were then stained in 1 µg/mL of EtBr in H_2O for 20 mins and de-stained in H_2O for 5 mins, before imaging with a UV gel documentation system (Syngene). The supercoiling extent was quantified using ImageJ software and IC_{50} values obtained from a derived form of the Hill equation.

Storage of reagents: topoisomerase IV should be stored at -80 °C and dilutions made fresh. Buffers, DNA and compound dilutions should be stored at -20 °C or below.

E. coli topo IV dilution buffer: HEPES.KOH pH 7.6 (40 mM), potassium glutamate (100 mM), DTT (1 mM), EDTA (1 mM) and 40% (v/v) glycerol.

E. coli topo IV assay buffer (1X recipe): HEPES.KOH pH 7.6 (40 mM), potassium glutamate (100 mM), magnesium acetate (10 mM), DTT (10 mM), ATP (1 mM) and albumin (50 µg/ml).

STEB: Sucrose 40% (w/v), Tris-HCl pH 8.0 (100 mM), EDTA (100 mM), Bromophenol Blue (0.5 mg/mL).

Determination of Minimum Inhibitory Concentration (MIC)

The MIC measurements of *E. coli* K12 MG1655, *E. coli* K12 MG1655 S83L or *S. aureus* NCIMB 50080 were determined using the standard broth microdilution assay[1] with minor modifications. Briefly, *E. coli* and *S. aureus* were grown in Mueller Hinton Broth (MHB (catalogue no. 70192; Sigma-Aldrich)) at 37˚C overnight, and then the cultures were diluted to OD_{600} = ~ 0.003 (~ 5 x 10^5 cells/mL).[2] 75 µL of the dilution was dispensed into the wells of a 96-well plate containing 75 µL of 2-fold dilutions of compounds. The first and last well of each row were used as only-MHB controls (blank). Each well was performed in triplicate. The plate was incubated for 18-20 hours in a shaker at 225 r.p.m. at 37˚C and the endpoint of OD_{600} were measured using a CLARIOstar® plate reader (BMG LABTECH). The blank-corrected mean value of each replicate was used to calculate the MIC value. The MIC value was designated as the lowest concentration that dramatically inhibits bacterial growth. Ciprofloxacin was used as a positive control in all cases.

Docking Protocol with Schrödinger

The protein structure (5NPP) was imported into Maestro[3] and prepared for molecular modelling using the default protein preparation wizard settings. No side chains were fixed/altered as any errors in the structure were distant to the allosteric site. Water molecules >5 Å from a heteroatom were removed and the protein minimised using the default settings. Following protein preparation, grid generation was undertaken using the 5NPP thiophene structure as the centre of the grid.

Putative inhibitors were built using the 2D sketcher or modified from existing SDF.file structures. All small molecule structures were prepared for docking using the LigPrep tool with default settings to generate appropriate 3D conformations.

The inhibitors were docked within the prepared grid using SP mode and default setting, only changing the output to sdf file. Docking results were visualised in Maestro using the in-built protein-ligand interactions toggle. Triaging of molecules for chemical synthesis was based upon visual inspection of the protein-ligand interactions, clashes with the protein and the docking score.

Supplementary Data

SPROUT *de novo* design run

SPROUT is a *de novo* design programme which allows the user to design small molecules from fragments using first principles. It is licenced *via* the University of Leeds.[4]

To create new ideas for the hydrophobic area of the allosteric pocket on gyrase, the following protocol was used. 5NPP was split into a receptor file (protein) and cavity file (thiophene **1**). Two target sites were created: a hydrophobic site 1 which overlays with the 2-chlorophenyl region of **1** (pink, below) and a spheric hydrophobic site 2 which overlays with the 2-methyl group on the thiophene ring (Figure S1). To site 1 was assigned 5- and 6-membered aromatic fragments, to site 2 a 5-membered aromatic fragment and a single sp^3 carbon atom. Methylene and benzene were selected as spacer templates, linking fragments between sites 1 and 2 that would generate whole molecular 'skeletons'. A total of 327 solutions were produced, which were scored *via* the SPROUT scoring function. This process consists of scoring the skeletons for predicted binding affinity to the selected target sites and steric constraints of the allosteric site. The skeletons were then re-ranked for molecular complexity and triaged by the authors (MJM & KMO).

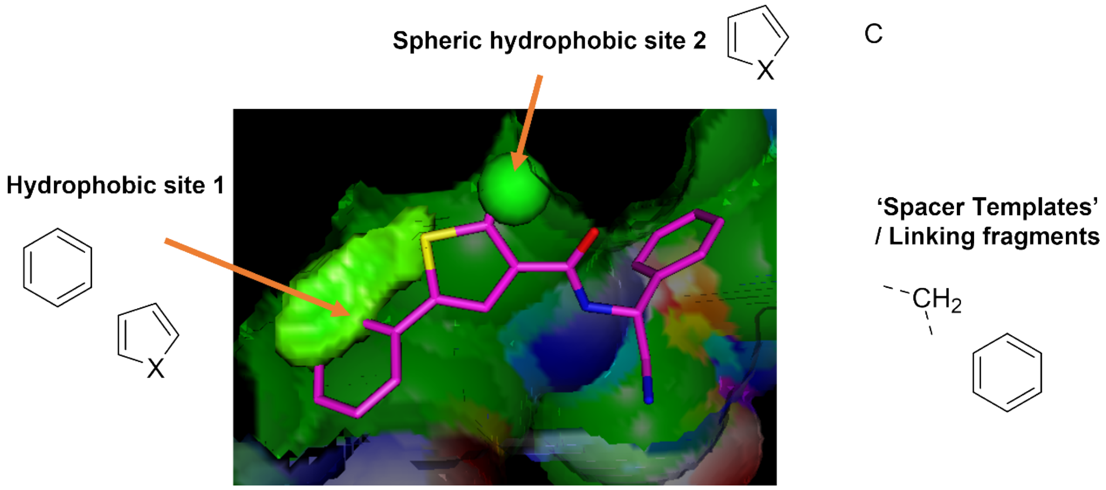

Figure S1 *De novo* design process in SPROUT showing the two targets sites 1 and 2, the selected fragment templates for each and the spacer templates used to generate whole skeletons.

Skeletons were selected if they satisfied the following criteria:

1) Covered both target sites 1 and 2

2) Contained a simple disconnection between the groups at sites 1 and 2

3) Contained a vector suitable for connecting to the chiral amine right hand portion of thiophene **1**.

Examples from the SPROUT design process can be seen below (Table 1S) where the SPROUT skeleton is shown in orange within the allosteric site overlaid with thiophene **1** (pink). All three skeletons satisfy criteria 1 and 2 but the bottom skeleton does not contain a suitable growth vector to satisfy criteria 3.

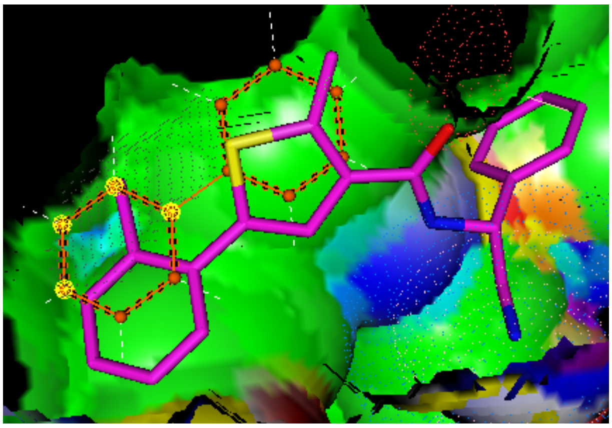	Skeleton 1 (biphenyl series – this work)
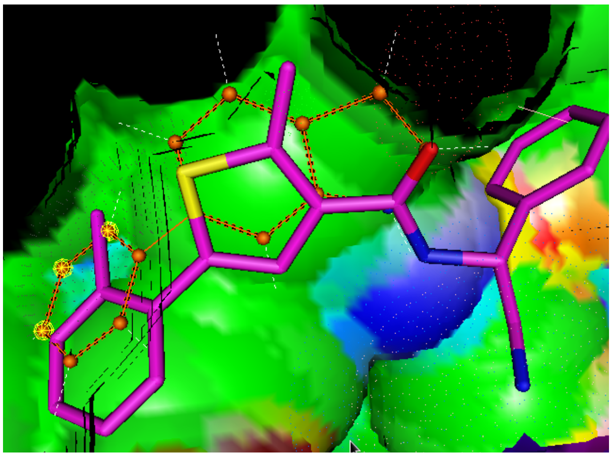	Skeleton 2 (indazole series – unpublished)
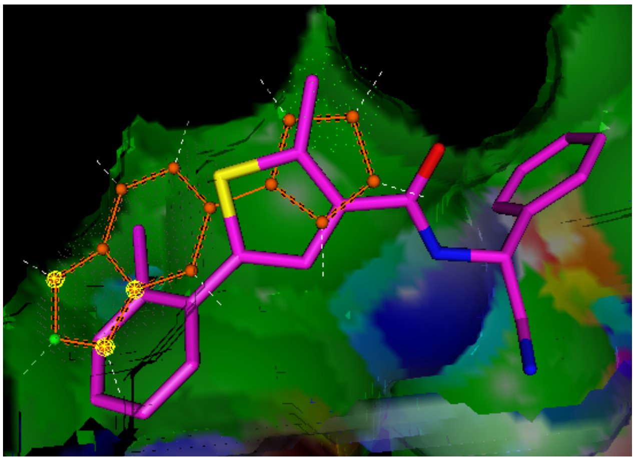	Skeleton 3 (not pursued)

Table S1 SPROUT results selected from the 327 solutions.

Docking Scores of biphenyl analogues

The Glide SP docking scores (DS) for compounds **1**-**33** are included in the Table 2S. The more negative the value, the better the predicted binding affinity. For reference, thiophene **1** was re-docked to the 5NPP allosteric site using the protocol above.

Compound	DS	Compound	DS	Compound	DS
**1**	-10.9	**10**	-10.7	**27**	N.D.
**2**	-10.4	**11**	-10.8	**28**	-9.6
**3**	-10.6	**12**	-10.9	**29**	-9.9
**4**	-10.7	**13**	-10.4	**30**	-9.8
**5**	-10.4	**14**	-10.7	**31**	-9.5
**6**	-10.7	**15**	N.D.	**32**	-9.3
**7**	-10.2	**24**	-10.7	**33**	-8.9
**8**	-11.1	**25**	-10.6		
**9**	-10.3	**26**	-10.3		

Table S2 Glide SP docking scores for the biphenyl series. N.D. = not determined.

Methyl Variants

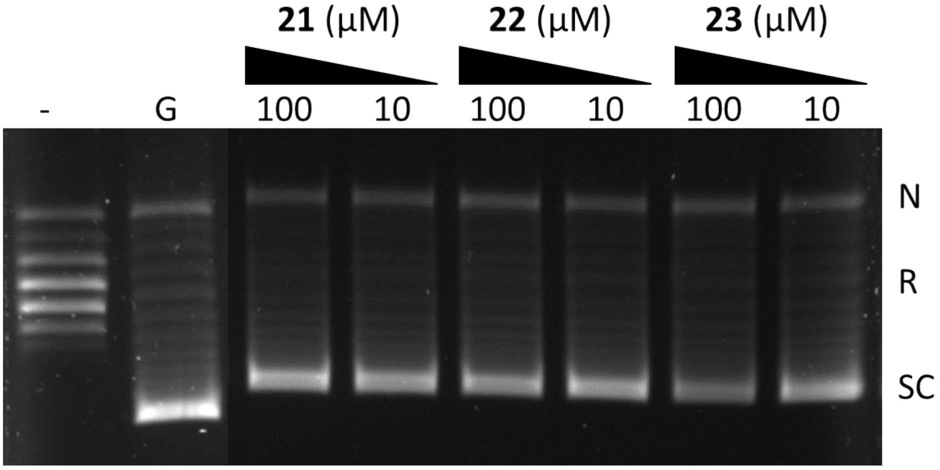

Figure S2 Gel electrophoresis data for compounds **21-23**. While IC_{50} values could not be determined for these compounds, visually compound **23** showed some inhibition at 100 μM which led to the synthesis of compounds **24-26**.

Comparison of biphenyl enantiomers

Compound	R	Gyrase IC_{50} (μM)	Docking Score
**2**	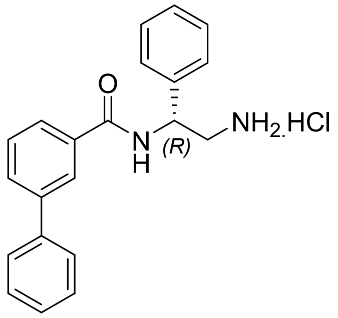	60	-10.4
**28**	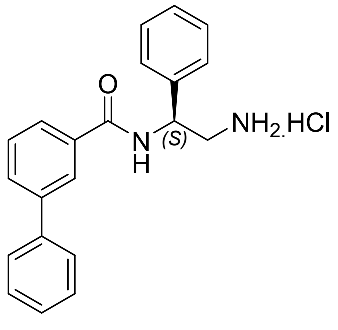	76	-9.6

Table S3. Comparison of biphenyl enantiomers (gyrase IC_{50} values and docking scores).

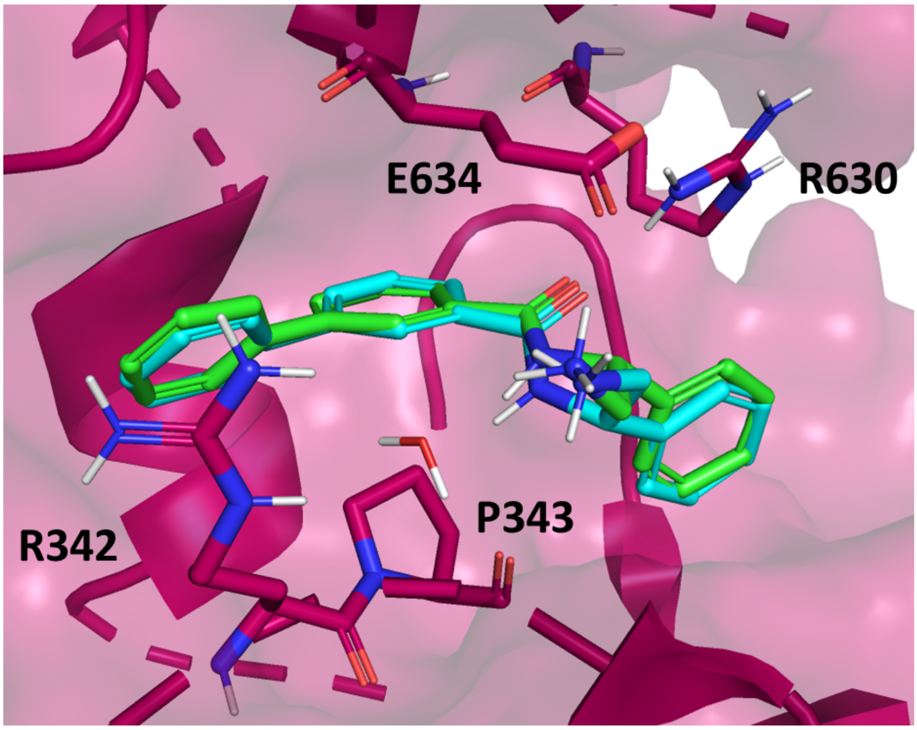

Figure S3 Comparison of Glide SP docked conformations of compounds **2** (green) and **28** (blue) with gyrase (5NPP) showing similar poses.

Synthesis of Pyridine-containing Compound **27**

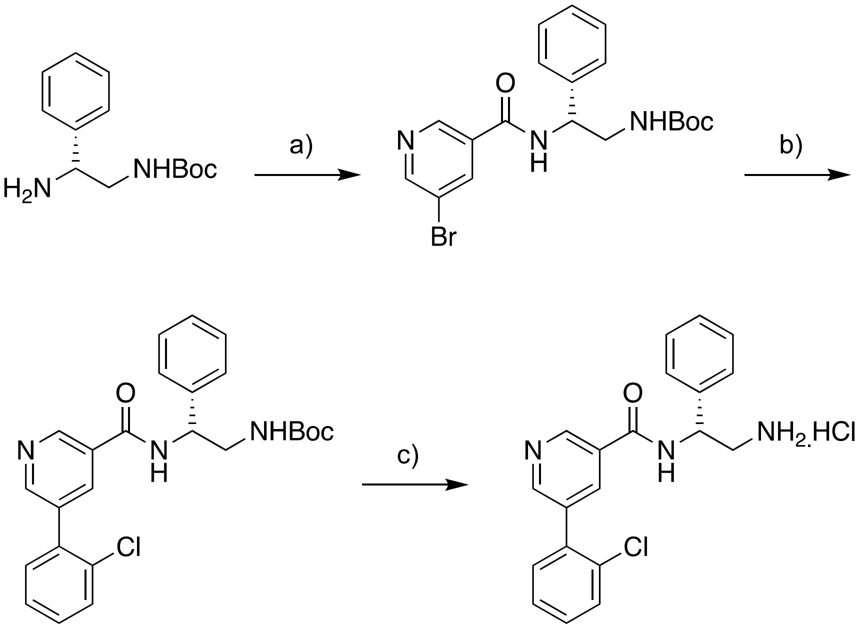

27

Scheme S1. Synthetic scheme for the synthesis of pyridine-containing compound **27**. Reagents and conditions: a) 5-bromopyridine-3-carboxylic acid, T3P, NEt_3, ethyl acetate, RT, 16 hours, 55%. b) 2-chlorophenylboronic acid, $Pd(PPh_3)_4$, K_2CO_3, dioxane:water, μW 140°C, 20 minutes, 70%. c) 4 N HCl in dioxane, RT, 1 hour, 78%.

Alternative Synthesis to Racemic Phenol Variants

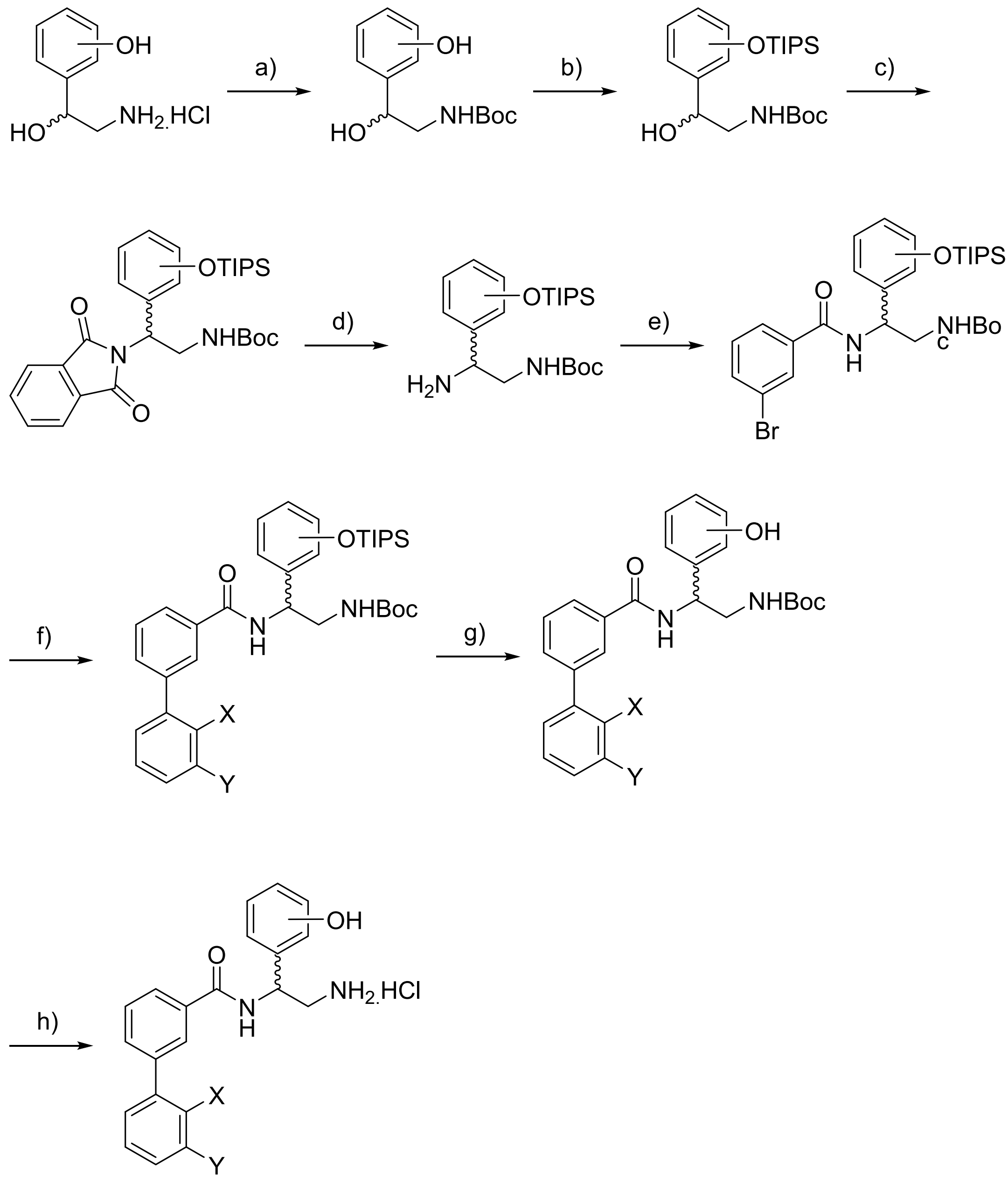

31-35

Scheme S2. Synthetic scheme for a library of phenolic bicyclic inhibitors. Reagents and conditions: a) Boc_2O, K_2CO_3, THF:H_2O, RT, 1 hour, 64-68%. b) Triisopropylsilyl chloride, imidazole, DMF, N_2, RT, 2 hours, 69-80%. c) Phthalimide, PPh_3, DEAD, THF, N_2, 0°C – RT, 18 hours, 33-44%. d) $N_2H_4 \cdot H_2O$, EtOH, 60°C, 2 hours, 84-88%. e) 3-bromobenzoyl chloride, NEt_3, DCM, N_2, RT, 20 hours, 41-64%. f) Substituted boronic acid, $Pd(PPh_3)_4$, 2M Na_2CO_3 aq., propanol, 90°C, 20 hours, 15-59%. g) 1 M TBAF in THF, THF, RT, 2 hours, 25-67%. h) 4 N HCl in dioxane, RT, 1 hour, 61-86%.

Reported Mass Spectrometry Data

The HR-MS data reported constitutes the largest peak that was observed in the spectrum irrespective of the counterion. This is reported to four decimal places. The mass observed is rationalised for each compound and includes the counterion in the mass calculation. In general, the accompanying counterion is H^+, Na^+ or K^+ and these are reported.

LC-MS reports data to two decimal places due to the significantly lower resolution capacity of this instrumentation. In some cases, there are counterions. In other cases, molecular units that are renowned for possessing weak stability, such as a Boc group, may have been stripped from the compound by ESI. This is a common phenomenon in mass spectrometry. For LC-MS data, neither counterions nor removed molecular groups are denoted, but an example below illustrates how the numbers are calculated.

For compound **18**, the following is reported:

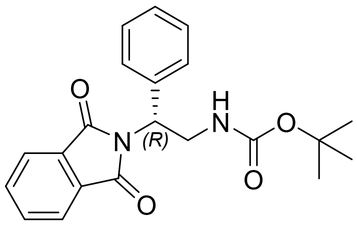

HR-MS, *m/z* (ES) found M+Na$^+$ 389.1475; $C_{21}H_{22}N_2O_4$ requires M+Na$^+$ 389.1471. **LC-MS**; RT = 0.65-0.70 min, *m/z* (ES) found 266.76.

For the HR-MS data, the mass (M) of **18** is 366.1580 to four decimal places. The mass plus a sodium cation (M+Na$^+$) is 389.1477. The value reported by the HR-MS is 389.1471. These two values differ by a reasonable degree of error, indicating the presence of **18**. For the LC-MS data, as the mass of **18** is 366.1580 but a mass of 266.76 has been reported, it is likely that the Boc group (M of cation is 101.06) was removed from **18** during ESI and the remaining amino anion was subsequently protonated. This species ($C_{16}H_{14}N_2O_2$) was then detected by the spectrometer. $C_{16}H_{14}N_2O_2$ would be expected to record a mass of 266.11 to two decimal places.

Compound Numbering for NMR Assignment

This section contains information regarding the procedure and methods adopted for numbering and assigning the NMR spectra of novel compounds synthesised throughout this project. Numbering of atoms follows a logical ascension primarily based as similarly as possible to the IUPAC name provided. Second to this, it is based on the addition of supplementary atomic chains, commonly in the form of aromatic rings (S3).

IUPAC Names:

Tert-butyl N-[(2S)-2-hydroxy-2-phenylethyl]carbamate (**17**)

Tert-butyl N-[(2R)-2-[(3-bromophenyl)formamido]-2-phenylethyl]carbamate (**20**)

N-[(1R)-2-amino-1-phenylethyl]-3'-ethoxy-2'-fluoro-[1,1'-biphenyl]-3-carboxamide hydrochloride (**6**)

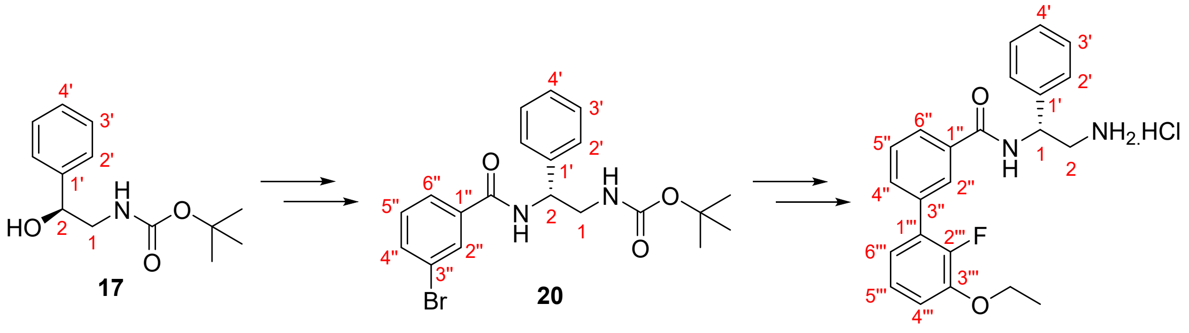

Scheme S3. Numbering of atomic chains within novel compounds in this manuscript

Following the first example **17**, C1 and C2 form the initial chain based on the IUPAC name specifying the order of said carbon atoms, *i.e.* with the hydroxy and phenylethyl motifs being at the 2-position. The phenyl ring constitutes a separate atomic chain, and therefore restarts numbering at C1' to allow for distinction. Following syntheses, the introduction of another atomic chain (in the case of a phenyl ring) across the carbonyl moiety of the amide restarts numbering for a second time at C1'', again following the IUPAC name of **20**. Following further syntheses, the introduction of a final atomic chain restarts numbering for a third time at C1''' for this chain in **6**. In this example, it is important to note the switch in nomenclature priority of C1 and C2 following the removal of a Boc-protecting group.

General Experimental Procedures

Method A: Suzuki Couplings

A flask charged with the chosen 3-bromo benzamide (1.0 eq.), the desired boronic acid (1.1 eq.) and $Pd(PPh_3)_4$ (0.05 eq.) was placed under a nitrogen atmosphere. Degassed propanol (2-5 mL) and 2 M aqueous Na_2CO_3 (3 eq.) were added. The resulting solution was refluxed for 4-20 hours before being cooled to RT. The mixture was filtered through a Celite pad and washed thoroughly with methanol before the solvent was stripped *in vacuo*. The resulting precipitate was then dissolved in ethyl acetate (20-50 mL) and washed with water (3 x 20-50 mL). The organics were dried ($MgSO_4$) before the solvent was again removed *in vacuo*. The crude product was purified using manual or automated column chromatography on silica gel to yield the title compounds. See individual compounds for detailed purification methods.

Method B: TIPS-Deprotection

To a solution of the chosen triisopropylsilane (1 eq.) in THF (10 mL), 1 M TBAF in THF (2.5 eq.) was added. The mixture was stirred at RT for 1-4 hours. The solvent was then stripped *in vacuo* and the crude residue purified using manual or automated flash column chromatography on silica gel to yield the title compounds. See individual compounds for detailed purification methods.

Method C: Boc-Deprotection

To a flask charged with the chosen Boc-protected amine (1 eq.), 4 N HCl in dioxane (1-5 mL) was added and the solution stirred at RT for 1-4 hours. The solvent was then stripped *in vacuo*. Hexane (20 mL) was added to the crude residue and the mixture sonicated for 5 minutes. A precipitate was released, filtered, washed with hexane, and the title compound obtained.

Preparation of Compounds

Tert-butyl N-[(2S)-2-hydroxy-2-phenylethyl]carbamate (**17**)

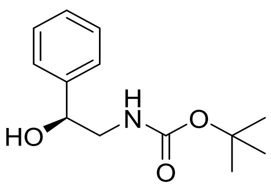

This procedure is modified from a literature source.[5] To a solution of (*S*)-2-amino-1-phenylethanol (2.02 g, 14.6 mmol) in THF (10 mL) at 0˚C, di-*tert*-butyl dicarbonate (3.60 mL, 15.5 mmol) was added. The solution was stirred at RT for 1 hour and then the solvent was removed *in vacuo*. Hexane (40 mL) was added and the suspension sonicated for 10 minutes. The solution was filtered to yield the title compound as a colourless solid (3.10 g, 13.0 mmol, 89%).

M.p. 62.5-64 ˚C. $\boldsymbol{R_f}$ 0.59 (50% ethyl acetate in petrol ether 40-60 ˚C). **^{1}H NMR (500 MHz, DMSO-*d*$_6$)**; δ 7.29-7.35 (m, 4H, C2'-H, C3'-H), 7.21-7.26 (m, 1H, C4'-H), 6.71 (app. t, *J* 5.4 Hz, 1H, N-H), 5.33 (s, 1H, O-H), 4.58 (t, *J* 6.0 Hz, 1H, C2-H), 3.09-3.15 (m, 1H, CH_2 syn or anti), 2.97-3.04, (m, 1H, CH_2 syn or anti), 1.36 (s, 9H, ^{t}Bu CH_3). **^{13}C NMR (125 MHz, DMSO-*d*$_6$)**; δ 156.0 (C=O), 144.1 (C1'), 128.4 (C3'), 127.4 (C4'), 126.5 (C2'), 78.0 ($C(CH_3)_3$), 71.9 (C2), 48.6 (C1), 28.7 (^{t}Bu CH_3). $\bar{\nu}_{max}$ **/cm^{-1} (solid)**; 3364 (N-H), 3291 (O-H), 2983 (C-H), 1666 (C=O). **HR-MS**, *m/z* (ES) found M+Na$^+$ 260.1261; $C_{13}H_{19}NO_3$ requires M+Na$^+$ 260.1257. **LC-MS**; RT = 0.50-0.55 min, *m/z* (ES) found 259.89.

Tert-butyl N-[(2R)-2-(1,3-dioxo-2,3-dihydro-1H-isoindol-2-yl)-2-phenylethyl]carbamate (**18**)

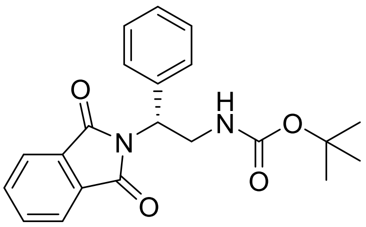

This procedure is modified from a literature source.[5] **17** (5.60 g, 23.5 mmol), phthalimide (3.48 g, 23.7 mmol) and triphenyl phosphine (7.40 g, 28.2 mmol) in anhydrous THF (40 mL) was placed under an atmosphere of N_2 and cooled to 0˚C. DEAD (4.44 mL, 28.2 mmol) was added dropwise over 10 minutes. The reaction mixture was then stirred for a further 15 minutes at 0 ˚C and then at RT for 18 hours. The solvent was then removed *in vacuo* and the yellow solid recrystallised from methanol to yield the title compound as a colourless solid (5.43 g, 14.8 mmol, 63%).

M.p. 103-107 ˚C. $\boldsymbol{R_f}$ 0.79 (50% ethyl acetate in petrol ether 40-60 ˚C). **^{1}H NMR (500 MHz, DMSO-*d*$_6$)**; δ 7.82-7.90 (m, 4H, C3''-H, C4''-H), 7.37-7.41 (m, 2H, C2'-H), 7.35 (app. t, *J* 7.3 Hz, 2H, C3'-H), 7.29 (app. t, *J* 7.3 Hz, 1H, C4'-H), 7.23 (app. t, *J* 5.9 Hz,

1H, N-H), 5.38 (dd, *J* 10.0, 4.6 Hz, 1H, C2-H), 3.91-3.99 (m, 1H, CH_2 syn or anti), 3.72-3.79 (m, 1H, CH_2 syn or anti), 1.27 (s, 9H, ^{t}Bu CH_3). **^{13}C NMR (125 MHz, DMSO-*d*$_6$)**; δ 168.3 (phthalimide C=O), 156.1 (carbamate C=O), 137.9 (C1'), 134.9 (C4''), 132.0 (C2''a), 128.9 (C3'), 128.1 (C2'), 127.8 (C4'), 123.5 (C3''), 78.3 ($C(CH_3)_3$), 54.1 (C2), 40.8 (C1), 28.5 (^{t}Bu CH_3). **$\bar{\nu}_{max}$ /cm^{-1} (solid)**; 3343 (N-H), 2968 (C-H), 1692 (C=O). **HR-MS**, *m/z* (ES) found M+Na$^+$ 389.1475; $C_{21}H_{22}N_2O_4$ requires M+Na$^+$ 389.1471. **LC-MS**; RT = 0.65-0.70 min, *m/z* (ES) found 266.76.

Tert-butyl [(2R)-2-amino-2-phenylethyl] carbamate (**19**)

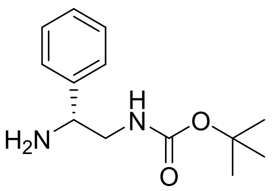

This procedure is modified from a literature source.[5] To a solution of **18** (5.10 g, 13.9 mmol) in ethanol (400 mL), hydrazine hydrate (3.7 mL, 118.9 mmol) was added. The mixture was stirred at 60˚C for 4 hours. An off-white precipitate had formed over this time. The reaction vessel was cooled to RT, filtered, and the solvent removed *in vacuo*. The filtrate was separated between ethyl acetate (100 mL) and water (100 mL). The aqueous layer was washed with ethyl acetate (3 x 30 mL), the organic washes combined and washed with brine (20 mL). The organic washes were then dried ($MgSO_4$), filtered, and the solvent stripped *in vacuo*. The yellow oil was dissolved in ethyl acetate (20 mL) and the solution triturated with petrol. ether 40-60˚C (100 mL) to release an off-white precipitate. The solution was filtered, and the filtrate concentrated *in vacuo* to yield the title compound as a colourless oil (2.38 g, 10.1 mmol, 74%).

***R*$_f$** 0.45 (10% MeOH in DCM). **^{1}H NMR (500 MHz, DMSO-*d*$_6$)**; δ 7.27-7.35 (m, 4H, C2'-H, C3'-H), 7.19-7.23 (m, 1H, C4'-H), 6.80 (app. t, *J* 5.1 Hz, 1H, carbamate N-H), 3.87 (dd, *J* 8.0, 5.1 Hz, 1H, C2-H), 3.07-3.14 (m, 1H, CH_2 syn or anti), 2.90-2.96 (m, 1H, CH_2 syn or anti) 1.36 (s, 9H, ^{t}Bu CH_3). **^{13}C NMR (125 MHz, DMSO-*d*$_6$)**; δ 156.1 (C=O), 145.3 (C1'), 128.4 (C3'), 127.1 (C4'), 127.0 (C2'), 78.0 ($C(CH_3)_3$), 55.6 (C2), 49.1 (C1), 28.5 (^{t}Bu CH_3). **$\bar{\nu}_{max}$ /cm^{-1} (oil)**; 3358 (N-H), 2975 (C-H), 1690 (C=O). **HR-MS**, *m/z* (ES) found M+Na$^+$ 259.1416; $C_{13}H_{20}N_2O_2$ requires M+Na$^+$ 259.1420. **LC-MS**; RT = 0.35-0.40 min, *m/z* (ES) found 180.67.

Tert-butyl N-[(2R)-2-[(3-bromophenyl)formamido]-2-phenylethyl]carbamate (**20**)

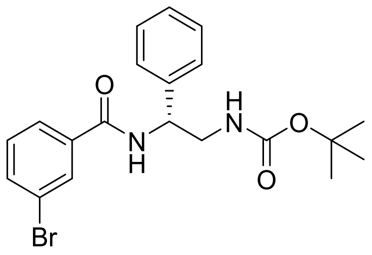

19 (215 mg, 0.91 mmol) in anhydrous DCM (10 mL) was placed under an atmosphere of N_2. Triethylamine (0.25 mL, 1.82 mmol) was added, followed by 3-bromobenzoyl chloride (0.12 mL, 0.91 mmol). The solution was stirred at RT for 22 hours. The resulting mixture was concentrated *in vacuo* and separated between ethyl acetate (20 mL) and water (20 mL). The organic layer was washed with water (20 mL) and brine (20 mL) before being dried ($MgSO_4$). The organic solvent was stripped *in vacuo* and the crude product recrystallised from 1:1 ethyl acetate:petrol ether 40-60˚C to yield the title compound as a colourless solid (164 mg, 0.39 mmol, 43%).

M.p. 160-161.5 °C. $\boldsymbol{R_f}$ 0.31 (25% ethyl acetate in petrol ether 40-60 ˚C). **^{1}H NMR (500 MHz, DMSO-d_6)**; 8.83 (app. d, *J* 8.2 Hz, 1H, amide N-H), 8.09 (app. s, 1H, C2''-H), 7.86 (app. d, *J* 8.2 Hz, 1H, C6''-H), 7.75 (app. d, *J* 8.2 Hz, 1H, C4''-H), 7.45 (app. t, *J* 8.2 Hz, 1H, C5''-H), 7.39 (app. d, *J* 7.3 Hz, 2H, C2'-H), 7.33 (app. t, *J* 7.3 Hz, 2H, C3'-H), 7.25 (app. t, *J* 7.3 Hz, 1H, C4'-H), 7.08 (app. t, *J* 6.0 Hz, 1H, carbamate N-H), 5.08-5.14 (m, 1H, C2-H), 3.34-3.41 (m, 1H, CH_2 syn or anti), 1.34 (s, 9H, ^{t}Bu CH_3). Second CH_2 syn or anti not observed (masked by H_2O). **^{13}C NMR (125 MHz, DMSO-d_6)**; δ 165.1 (amide C=O), 156.4 (carbamate C=O), 141.6 (C1'), 137.1 (C1''), 134.4 (C4''), 131.0 (C5''), 130.5 (C2''), 128.7 (C3'), 127.5 (C4'), 127.3 (C2'), 127.2 (C6''), 122.0 (C3''), 78.4 ($C(CH_3)_3$), 54.6 (C2), 45.5 (C1), 28.7 (^{t}Bu CH_3). $\bar{\nu}_{max}$ **/cm^{-1} (solid)**; 3371 (N-H), 2978 (C-H), 1687 (carbamate C=O), 1638 (amide C=O). **HR-MS**, *m/z* (ES) found M+Na$^+$ 443.0765; $C_{20}H_{23}BrN_2O_3$ requires M+Na$^+$, 443.0789. **LC-MS**; RT = 0.60-0.65 min, *m/z* (ES) found 318.89.

Tert-butyl (R)-(2-(5-bromonicotinamido)-2-phenylethyl)carbamate

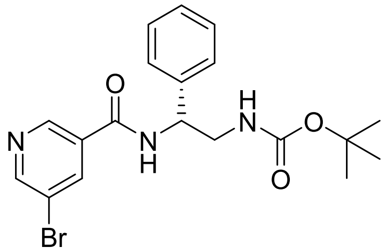

5-Bromopyridine-3-carboxylic acid (85 mg, 0.42 mmol) in ethyl acetate (4 mL) was treated with propanephosphonic acid anhydride (300 µL, 0.51 mmol, 50% in ethyl acetate) and triethylamine (90 µL, 0.63 mmol), and stirred at RT for 20 minutes. **19** (100 mg, 0.42 mmol) was added and stirred at RT for 16 hours. The reaction mixture was diluted with ethyl acetate (5 mL), washed with water (3 x 10 mL), the organic layer collected and

dried ($MgSO_4$). The filtrate was collected and the solvent stripped *in vacuo*. The crude was purified through flash column chromatography (1:1 ethyl acetate:hexane) to yield the title compound as a white solid (96 mg, 0.23 mmol, 55%).

***R*f** 0.55 (50% ethyl acetate in hexane). **^{1}H NMR (500 MHz, $CDCl_3$)**; δ 9.02 (d, *J* 1.40 Hz, 1H, C6''-H), 8.76 (d, *J* 2.24 Hz, 1H, C4''-H), 8.53 (d, *J* 5.33 Hz, 1H, C2''-H), 8.32 (s, 1H, amide N-H), 7.35 – 7. 25 (m, 5H, C1'-5'-H), 5.10 (m, 2H, C2-H, carbamate N-H), 3.63-3.57 (m, 1H, CH_2, syn or anti), 3.44 (ddd, *J* = 15.1 6.5 3.0 Hz, 1H, CH_2, syn or anti), 1.44 (s, 9H, ^{t}Bu CH_3). **^{13}C NMR (125 MHz, $CDCl_3$)**; δ 163.7 (amide C=O), 158.6 (carbamate C=O), 153.2 (C4''), 146.6 (C6''), 139.3 (C1'), 137.7 (C2''), 130.9 (C1''), 128.8 (C3'), 127.8 (C4'), 126.4 (C2'), 120.8 (C2''), 80.9 ($C(CH_3)_3$), 57.5 (C2), 45.8 (C1), 28.4 (^{t}Bu CH_3). **LC-MS**; RT = 0.6-0.7 min, *m/z* (ES) found 419.88.

Tert-butyl (R)-(2-(5-(2-chlorophenyl)nicotinamido)-2-phenylethyl)carbamate

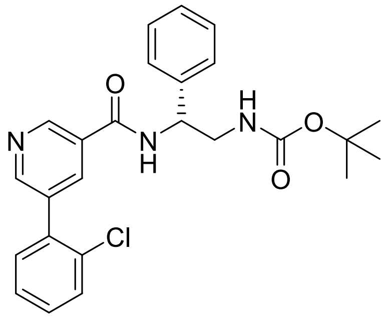

A flask charged with *tert*-butyl (*R*)-(2-(5-bromonicotinamido)-2-phenylethyl)carbamate (20 mg, 0.05 mmol), 2-chlorophenylboronic acid (7 mg, 0.05 mmol), $Pd(PPh_3)_4$ (6 mg, 0.005 mmol) and K_2CO_3 (16 mg, 0.12 mmol) was placed under a nitrogen atmosphere. A degassed mixture of 1,4-dioxane/water (3:1, 3 mL) was added. The resulting solution was heated using microwave irradiation at 140°C for 20 minutes before being cooled to RT. The mixture was filtered through a Celite pad and washed thoroughly with methanol before the solvent was stripped *in vacuo*. The resulting crude was then purified through flash column chromatography (1:1 ethyl acetate:hexane) to yield the title compound as a white solid (15 mg, 0.033 mmol, 70%).

^{1}H NMR (500 MHz, $CDCl_3$); δ 9.12 (s, 1H, C4''-H), 8.80 (d, *J* 1.4 Hz, 1H, C6''-H), 8.48 (d, *J* 5.2 Hz, 1H, C2''-H), 8.26 (s, 1H, amide N-H), 7.51 – 7.50 (m, 1H, C6'''-H), 7.37 – 7.34 (m, 7H, C1'-3',2'''-5'''-H), 7.29 – 7.26 (m, 1H, C4''-H), 5.16 (app. t, *J* 5.8 Hz, 1H), 5.03 (app. t, *J* 5.8 Hz, 1H), 3.62 (app. p, *J* 7.4 Hz, 1H, CH_2 syn or anti), 3.47 (ddd, *J* 14.7 5.7 2.3 Hz, 1H), 1.38 (s, 9H, ^{t}Bu CH_3). **^{13}C NMR (125 MHz, $CDCl_3$)**; δ 165.1 (amide C=O), 158.7 (carbamate C=O), 152.6 (C4''), 147.6 (C6''), 139.7 (C1'), 136.4 (C1'''), 135.9 (C6'''), 135.1 (C3''), 132.9 (C2'''), 131.5 (C3'''), 130.3 (C4'''), 129.9 (C5'''), 129.2 (C1''), 129.0 (C3'), 127.9 (C4'), 127.4 (C2''), 126.6 (C2'), 81.0 ($C(CH_3)_3$), 57.6 (C2), 46.0 (C1), 28.5 ((^{t}Bu CH_3).

Tert-butyl N-[2-hydroxy-2-(3-hydroxyphenyl)ethyl]carbamate

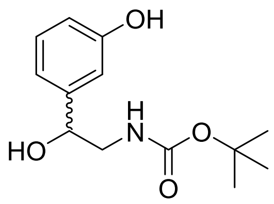

To a solution of 3-(2-amino-1-hydroxyethyl) phenol hydrochloride (2.01 g, 10.5 mmol) and K_2CO_3 (2.90 g, 21.0 mmol) in a 1:1 mixture of THF:H_2O (40 mL), di-*tert*-butyl dicarbonate (2.80 mL, 12.2 mmol) was added at 0°C. The solution was stirred at RT for 1 hour then the solvent stripped *in vacuo*. The crude solid was separated between ethyl acetate (40 mL) and water (40 mL), dried ($MgSO_4$) and the solvent removed *in vacuo*. Hexane (40 mL) was added and the mixture stirred at RT for 1 hour. The colourless solid was then filtered to yield the title compound (1.94 g, 6.7 mmol, 64%).

M.p. 106-108 °C. ***R*f** 0.49 (50% ethyl acetate in petrol ether 40-60 ˚C). **^{1}H NMR (500 MHz, DMSO-*d*₆)**; δ 9.24 (s, 1H, C3'-OH), 7.09 (app. t, *J* 7.6 Hz, 1H, C5'-H), 6.74 (app. s, 1H, C2'-H), 6.71 (app. d, *J* 7.6 Hz, 1H, C6'-H), 6.64-6.66 (m, 1H, N-H), 6.62 (app. ddd, *J* 8.0, 2.4, 0.9 Hz, 1H, C4'-H), 5.24 (d, *J* 4.6 Hz, 1H, O-H), 4.49 (dd, *J* 8.5, 4.6 Hz, 1H, C2-H), 3.09 (ddd, *J* 13.1, 6.1, 4.6 Hz, 1H, CH_2 syn or anti). 2.93-2.99 (m, 1H, CH_2 syn or anti), 1.37 (s, 9H, ^{t}Bu CH_3). **^{13}C NMR (125 MHz, DMSO-d_6)**; δ 157.6 (C3'), 156.1 (C=O), 145.7 (C1'), 129.3 (C5'), 117.1 (C6'), 114.3 (C4'), 113.4 (C2'), 78.0 ($C(CH_3)_3$), 71.9 (C2), 48.7 (CH_2), 28.7 (^{t}Bu CH_3). $\bar{\nu}_{max}$ **/cm^{-1}**; 3396 (N-H), 3267 (O-H), 2972 (C-H), 1645 (C=O). **HR-MS**, *m/z* (ES) found M+Na$^+$ 276.1201; $C_{13}H_{19}NO_4$ requires M+Na$^+$ 276.1211. **LC-MS**; RT = 0.50-0.55 min, *m/z* (ES) found 276.08.

Tert-butyl N-[2-hydroxy-2-(4-hydroxyphenyl)ethyl]carbamate

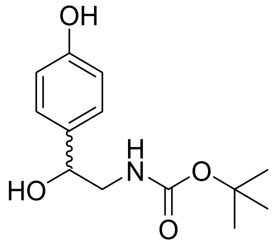

To a solution of octopamine hydrochloride (2.01 g, 10.5 mmol) and K_2CO_3 (2.90 g, 21.0 mmol) in a 1:1 mixture of THF:H_2O (40 mL), di-*tert*-butyl dicarbonate (2.80 mL, 12.2 mmol) was added at 0 °C. The solution was stirred at RT for 1 hour then the solvent stripped *in vacuo*. The crude solid was separated between ethyl acetate (40 mL) and water (40 mL), dried ($MgSO_4$) and the solvent removed *in vacuo*. Hexane (40 mL) was added and the mixture stirred at RT for 1 hour. The tan solid was then filtered to yield the title compound (2.07 g, 7.2 mmol, 68%).

M.p. 140-142 °C. ***R*f** 0.49 (50% ethyl acetate in petrol ether 40-60 ˚C). **^{1}H NMR (500 MHz, DMSO-d_6)**; δ 9.21 (s, 1H, C4'-OH), 7.10 (app. d, *J* 8.5 Hz, 2H, C2'-H), 6.70 (app. d, *J* 8.5 Hz, 2H, C3'-H), 6.59 (app. t, *J* 5.5 Hz, 1H, N-H), 5.12 (d, *J* 4.5 Hz, 1H, O-H), 4.47 (dd, *J* 8.4, 4.5 Hz, 1H, C2-H), 3.04-3.10 (m, 1H, CH_2 syn or anti), 2.94-3.00 (m,

1H, C_H_$_2$ syn or anti), 1.36 (s, 9H, ^{t}Bu C_H_$_3$). **^{13}C NMR (125 MHz, DMSO-*d*$_6$)**; δ 156.8 (C4'), 156.1 (C=O), 134.4 (C1'), 127.6 (C2'), 115.2 (C3'), 78.0 (_C_(CH$_3$)$_3$), 71.6 (C2), 48.7 (_C_H$_2$), 28.7 (^{t}Bu _C_H$_3$). $\bar{\nu}_{max}$ **/cm^{-1}**; 3466 (N-H), 3148 (O-H), 2977 (C-H), 1659 (C=O). **HR-MS**, *m/z* (ES) found M+Na$^+$ 276.1203; $C_{13}H_{19}NO_4$ requires M+Na$^+$ 276.1211. **LC-MS**; RT = 0.50-0.55 min, *m/z* (ES) found 276.08.

Tert-butyl N-[2-hydroxy-2-(3-{[tris(propan-2-yl)silyl]oxy}phenyl)ethyl]carbamate

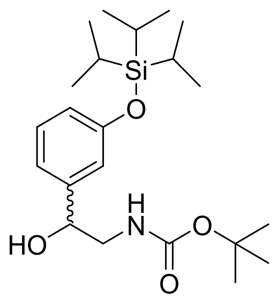

A flask charged with *tert*-butyl *N*-[2-hydroxy-2-(3-hydroxyphenyl)ethyl]carbamate (1.94 g, 6.7 mmol) and imidazole (949 mg, 14.0 mmol) was placed under an atmosphere of N_2. Anhydrous DMF (5 mL) was added, followed by triisopropylsilyl chloride (1.45 mL, 6.7 mmol). The reaction was stirred at RT for 2 hours. Ethyl acetate (20 mL) was then added to the mixture, and the colourless precipitate filtered off. The organics were washed with 10% LiCl (w/v) aqueous solution (2 x 20 mL). The aqueous layer was washed with ethyl acetate (20 mL), the organics combined, dried ($MgSO_4$) and the solvent stripped *in vacuo*. The colourless oil was purified using automated flash column chromatography (0:100 – 30:70 ethyl acetate:petrol ether 40-60°C) to yield the title compound as a colourless oil (1.89 g, 4.6 mmol, 69%).

***R*$_f$** 0.80 (50% ethyl acetate in petrol ether 40-60 °C). **^{1}H NMR (500 MHz, DMSO-*d*$_6$)**; δ 7.18 (app. t, *J* 7.8 Hz, 1H, C5'-_H_), 6.88 (app. d, *J* 7.8 Hz, 1H, C6'-_H_), 6.83 (app. s, 1H, C2'-_H_), 6.72 (app. dd, *J* 7.8, 2.3 Hz, 1H, C4'-_H_), 6.62 (app. t, *J* 5.3 Hz, 1H, N-_H_), 5.31 (d, *J* 4.5 Hz, 1H, O-_H_), 4.50-4.55 (m, 1H, C2-_H_), 3.05-3.11 (m, 1H, C_H_$_2$ syn or anti), 2.94-3.01 (m, 1H, C_H_$_2$ syn or anti), 1.35 (s, 9H, ^{t}Bu C_H_$_3$), 1.24 (septet, *J* 7.5 Hz, 3H, Si^{i}Pr C-_H_), 1.07 (d, *J* 7.5 Hz, 18H, Si^{i}Pr C_H_$_3$). **^{13}C NMR (125 MHz, DMSO-*d*$_6$)**; δ 156.1 (C=O), 155.6 (C3'), 146.0 (C1'), 129.5 (C5'), 119.3 (C6'), 118.4 (C4'), 117.7 (C2'), 78.0 (_C_(CH$_3$)$_3$), 71.6 (C2), 48.7 (_C_H$_2$), 28.7 (^{t}Bu _C_H$_3$), 18.2 (Si^{i}Pr _C_H$_3$), 12.5 (Si^{i}Pr _C_-H). $\bar{\nu}_{max}$ **/cm^{-1}**; 3408 (N-H), 2943 (C-H), 1691 (C=O). **HR-MS**, *m/z* (ES) found M+Na$^+$ 432.2543; $C_{22}H_{39}NO_4Si$ requires M+Na$^+$ 432.2546. **LC-MS**; RT = 0.85-0.90 min, *m/z* (ES) found 292.21.

Tert-butyl N-[2-hydroxy-2-(4-{[tris(propan-2-yl)silyl]oxy}phenyl)ethyl]carbamate

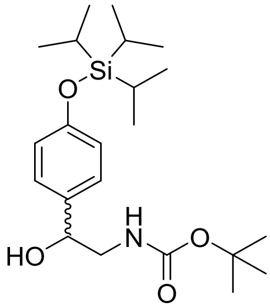

A flask charged with *tert*-butyl *N*-[2-hydroxy-2-(4-hydroxyphenyl)ethyl]carbamate (1.30 g, 4.5 mmol) and imidazole (628 mg, 9.0 mmol) was placed under an atmosphere of N_2. Anhydrous DMF (5 mL) was added, followed by triisopropylsilyl chloride (1.0 mL, 4.5 mmol). The reaction was stirred at RT for 2 hours. Ethyl acetate (20 mL) was then added to the mixture, and the colourless precipitate filtered off. The organics were washed with 10% LiCl (w/v) aqueous solution (2 x 20 mL). The aqueous layer was washed with ethyl acetate (20 mL), the organics combined, dried ($MgSO_4$) and the solvent stripped *in vacuo*. The crude product was purified using automated flash column chromatography (0:100 – 30:70 ethyl acetate:petrol ether 40-60°C) to yield the title compound as a colourless solid (1.46 g, 3.6 mmol, 80%).

M.p. 114-116.5 °C. $\boldsymbol{R_f}$ 0.80 (50% ethyl acetate in petrol ether 40-60 ˚C). **^{1}H NMR (500 MHz, DMSO-*d*$_6$)**; δ 7.19 (app. d, *J* 8.5 Hz, 2H, C2'-H), 6.81 (app. d, *J* 8.5 Hz, 2H, C3'-H), 6.62 (app. t, *J* 5.5 Hz, 1H, N-H), 5.22 (d, *J* 4.4 Hz, 1H, O-H), 4.49-4.53 (m, 1H, C2-H), 3.07-3.13 (m, 1H, CH_2 syn or anti), 2.96-3.02 (m, 1H, CH_2 syn or anti), 1.35 (s, 9H, ^{t}Bu CH_3), 1.25 (septet, *J* 7.5 Hz, 3H, Si^{i}Pr C-H), 1.07 (d, *J* 7.5 Hz, 18H, Si^{i}Pr CH_3). **^{13}C NMR (125 MHz, DMSO-*d*$_6$)**; δ 156.0 (C=O), 154.8 (C4'), 136.8 (C1'), 127.7 (C2'), 119.4 (C3'), 78.0 ($C(CH_3)_3$), 71.6 (C2), 48.5 (CH_2), 28.7 (^{t}Bu CH_3), 18.2 (Si^{i}Pr CH_3), 12.5 (Si^{i}Pr C-H).). $\bar{\nu}_{max}$ **/cm^{-1}**; 3326 (N-H), 2942 (C-H), 1684 (C=O). **HR-MS**, *m/z* (ES) found M+Na$^+$ 432.2543; $C_{22}H_{39}NO_4Si$ requires M+Na$^+$ 432.2546. **LC-MS**; RT = 0.85-0.90 min, *m/z* (ES) found 336.32.

Tert-butyl N-[2-(1,3-dioxo-2,3-dihydro-1H-isoindol-2-yl)-2-(3-{[tris(propan-2-yl)silyl]oxy}phenyl)ethyl]carbamate

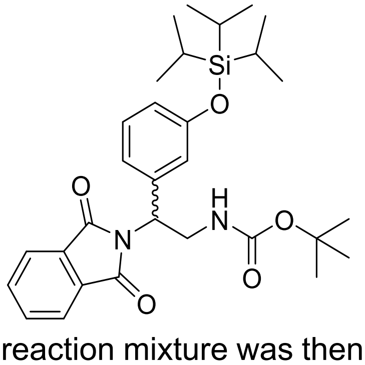

Tert-butyl *N*-[2-hydroxy-2-(3-{[tris(propan-2-yl)silyl]oxy}phenyl)ethyl]carbamate (1.89 g, 4.7 mmol), phthalimide (685 mg, 4.7 mmol) and triphenyl phosphine (1.45 g, 5.6 mmol) in anhydrous THF (40 mL) was placed under an atmosphere of N_2 and cooled to 0°C. DEAD (0.88 mL, 5.6 mmol) was added dropwise over 10 minutes. The reaction mixture was then stirred for a further 15 minutes at 0 ˚C and then at RT for 18

hours. The solvent was removed *in vacuo* and the crude mixture triturated with diethyl ether. The colourless solid was removed and the organic solvent removed *in vacuo*. The crude product was purified using automated flash column chromatography (0:100 – 20:80 ethyl acetate:petrol ether 40-60°C) to yield the title compound as a colourless solid (1.10 g, 2.0 mmol, 44%).

M.p. 70-72 °C. R_f 0.57 (25% ethyl acetate in petrol ether 40-60 °C). **^{1}H NMR (500 MHz, DMSO-d_6)**; δ 7.85-7.90 (m, 4H, phthalimide C3''-H, C4''-H), 7.24 (app. t, *J* 7.8 Hz, 1H, C5'-H), 7.20 (app. t, *J* 5.0 Hz, 1H, N-H), 7.00 (app. dt, *J* 7.7, 0.7 Hz, 1H, C6'-H), 6.84 (app. s, 1H, C2'-H), 6.80 (app. dd, *J* 8.0, 2.0 Hz, 1H, C4'-H), 5.34 (dd, *J* 9.5, 4.6 Hz, 1H, C2-H), 3.88-3.95 (m, 1H, CH_2 syn or anti), 3.74-3.80 (m, 1H, CH_2 syn or anti), 1.29 (s, 9H, , ^{t}Bu CH_3), 1.11-1.20 (m, 3H, Si^{i}Pr C-H), 0.99 (d, *J* 7.5 Hz, 18H, Si^{i}Pr CH_3). **^{13}C NMR (125 MHz, DMSO-d_6)**; δ 168.3 (phthalimide C=O), 156.2 (carbamate C=O), 155.9 (C3'), 139.6 (C1'), 135.0 (C4''), 131.9 (C2''a), 130.2 (C5'), 123.5 (C3''), 120.6 (C6'), 119.4 (C4'), 118.7 (C2'), 78.4 ($C(CH_3)_3$), 54.0 (C2), 40.8 (CH_2), 28.5 (^{t}Bu CH_3), 18.1 (Si^{i}Pr CH_3), 12.4 (Si^{i}Pr C-H). $\bar{\nu}_{max}$ **/cm^{-1}**; 3286 (N-H), 2943 (C-H), 1712 (phthalimide C=O), 1695 (carbamate C=O). **HR-MS**, *m/z* (ES) found M+Na$^+$ 561.2760; $C_{30}H_{42}N_2O_5Si$ requires M+Na$^+$ 561.2760. **LC-MS**; RT = 0.90-0.95 min, *m/z* (ES) found 439.53.

Tert-butyl N-[2-(1,3-dioxo-2,3-dihydro-1H-isoindol-2-yl)-2-(4-{[tris(propan-2-yl)silyl]oxy}phenyl)ethyl]carbamate

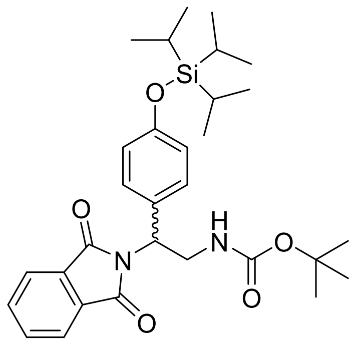

Tert-butyl *N*-[2-hydroxy-2-(4-{[tris(propan-2-yl)silyl]oxy}phenyl)ethyl]carbamate (1.46 g, 3.6 mmol), phthalimide (0.53 g, 3.6 mmol) and triphenyl phosphine (1.13 g, 4.3 mmol) in anhydrous THF (40 mL) was placed under an atmosphere of N_2 and cooled to 0°C. DEAD (0.68 mL, 4.3 mmol) was added dropwise over 10 minutes. The reaction mixture was then stirred for a further 15 minutes at 0 °C and then at RT for 18 hours. The solvent was removed *in vacuo* and the crude mixture triturated with diethyl ether. The colourless solid was removed and the organic solvent removed *in vacuo*. The crude product was purified using automated flash column chromatography (0:100 – 20:80 ethyl acetate:petrol ether 40-60°C) to yield the title compound as a colourless solid (625 mg, 1.2 mmol, 33%).

M.p. 164-167 °C. $\boldsymbol{R_f}$ 0.57 (25% ethyl acetate in petrol ether 40-60 ˚C). **^{1}H NMR (500 MHz, DMSO-d_6)**; δ 7.82-7.87 (m, 4H, phthalimide C3''-H, C4''-H), 7.28 (app. d, *J* 8.5 Hz, 2H, C2'-H), 7.17 (app. t, *J* 5.5 Hz, 1H, N-H), 6.82 (app. d, *J* 8.5 Hz, 2H, C3'-H), 5.30 (dd, *J* 10.0, 4.7 Hz, 1H, C2-H), 3.87-3.94 (m, 1H, CH_2 syn or anti), 3.66-3.73 (m, 1H, CH_2 syn or anti), 1.26 (s, 9H, ^{t}Bu CH_3), 1.19-1.25 (m, 3H, Si^{i}Pr C-H), 1.04 (d, *J* 7.5 Hz, 18H, Si^{i}Pr CH_3). **^{13}C NMR (125 MHz, DMSO-d_6)**; δ 168.3 (phthalimide C=O), 156.2 (carbamate C=O), 155.3 (C4'), 134.9 (C4''), 133.1 (C1'), 132.0 (C2''a), 129.3 (C2'), 123.5 (C3''), 119.8 (C3'), 78.3 ($\underline{C}(CH_3)_3$), 53.7 (C2), 40.9 ($\underline{C}H_2$), 28.5 (^{t}Bu $\underline{C}H_3$), 18.2 (Si^{i}Pr $\underline{C}H_3$), 12.5 (Si^{i}Pr C-H). $\bar{\nu}_{max}$ **/cm^{-1}**; 3304 (N-H), 2944 (C-H), 1713 (phthalimide C=O), 1692 (carbamate C=O). **HR-MS**, *m/z* (ES) found M+Na$^+$ 561.2760; $C_{30}H_{42}N_2O_5Si$ requires M+Na$^+$ 561.2760. **LC-MS**; RT = 0.90-0.95 min, *m/z* (ES) found 439.53.

Tert-butyl N-[2-amino-2-(3-{[tris(propan-2-yl)silyl]oxy}phenyl)ethyl]carbamate

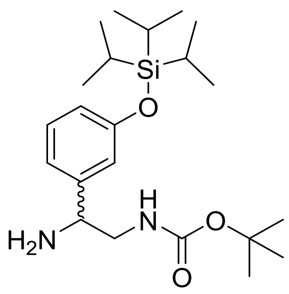

To a solution of *tert*-butyl *N*-[2-(1,3-dioxo-2,3-dihydro-1H-isoindol-2-yl)-2-(3-{[tris(propan-2-yl)silyl]oxy}phenyl)ethyl]carbamate (1.08 g, 2.0 mmol) in ethanol (20 mL), hydrazine hydrate (0.5 mL, 16.0 mmol) was added. The mixture was stirred at 60°C for 4 hours. An off-white precipitate had formed over this time. The reaction vessel was cooled to RT, filtered, and the solvent removed *in vacuo* to yield the title compound as a pale yellow oil (715 mg, 1.8 mmol, 88%).

$\boldsymbol{R_f}$ 0.54 (10% MeOH in DCM). **^{1}H NMR (500 MHz, DMSO-d_6)**; δ 7.16 (app. t, *J* 7.8 Hz, 1H, C5'-H), 6.90 (app. d, *J* 7.8 Hz, 1H, C6'-H), 6.86 (app. s, 1H, C2'-H), 6.69 (app. dd, *J* 7.8, 1.9 Hz, 1H, C4'-H), 3.82 (dd, *J* 7.8, 5.1 Hz, 1H, C2-H), 3.03-3.09 (m, 1H, CH_2 syn or anti), 2.86-2.93 (m, 1H, CH_2 syn or anti), 1.35 (s, 9H, ^{t}Bu CH_3), 1.24 (septet, *J* 7.5 Hz, 3H, Si^{i}Pr C-H), 1.06 (d, *J* 7.5 Hz, 18H, Si^{i}Pr CH_3). **^{13}C NMR (125 MHz, DMSO-d_6)**; δ 156.1 (C=O), 155.7 (C3'), 147.2 (C1'), 129.5 (C5'), 120.0 (C6'), 118.4 (C4'), 118.1 (C2'), 78.0 ($\underline{C}(CH_3)_3$), 55.3 (C2), 49.2 ($\underline{C}H_2$), 28.7 (^{t}Bu $\underline{C}H_3$), 18.3 (Si^{i}Pr $\underline{C}H_3$), 12.6 (Si^{i}Pr C-H). $\bar{\nu}_{max}$ **/cm^{-1}**; 3346 (N-H), 2943 (C-H), 1706 (C=O). **HR-MS**, *m/z* (ES) found M+H$^+$ 409.2889; $C_{22}H_{40}N_2O_3Si$ requires M+H$^+$ 409.2886. **LC-MS**; RT = 0.65-0.70 min, *m/z* (ES) found 409.49.

Tert-butyl N-[2-amino-2-(4-{[tris(propan-2-yl)silyl]oxy}phenyl)ethyl]carbamate

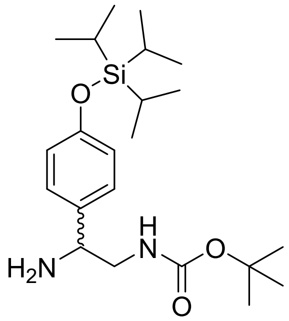

To a solution of *tert*-butyl *N*-[2-(1,3-dioxo-2,3-dihydro-1H-isoindol-2-yl)-2-(4-{[tris(propan-2-yl)silyl]oxy}phenyl)ethyl]carbamate (610 mg, 1.1 mmol) in ethanol (20 mL), hydrazine hydrate (0.3 mL, 9.0 mmol) was added. The mixture was stirred at 60°C for 4 hours. An off-white precipitate had formed over this time. The reaction vessel was cooled to RT, filtered, and the solvent removed *in vacuo* to yield the title compound as a pale yellow oil (375 mg, 0.9 mmol, 84%).

***R*$_f$** 0.54 (10% MeOH in DCM). **^{1}H NMR (500 MHz, DMSO-*d*$_6$)**; δ 7.21 (app. d, *J* 8.5 Hz, 2H, C2'-H), 6.80 (app. d, *J* 8.5 Hz, 2H, C3'-H), 6.75 (app. t, *J* 5.5 Hz, 1H, N-H), 3.84-3.88 (m, 1H, C2-H), 3.07-3.13 (m, 1H, CH_2 syn or anti), 2.92-2.99 (m, 1H, CH_2 syn or anti), 1.35 (s, 9H, ^{t}Bu CH_3), 1.23 (septet, *J* 7.5 Hz, 3H, Si^{i}Pr C-H), 1.05 (d, *J* 7.5 Hz, 18H, Si^{i}Pr CH_3). **^{13}C NMR (125 MHz, DMSO-*d*$_6$)**; δ 156.1 (C=O), 154.7 (C4'), 136.6 (C1'), 128.4 (C2'), 119.5 (C3'), 78.0 ($C(CH_3)_3$), 54.9 (C2), 49.1 (CH_2), 28.7 (^{t}Bu CH_3), 18.2 (Si^{i}Pr CH_3), 12.5 (Si^{i}Pr C-H). $\bar{\nu}_{max}$ **/cm^{-1}**; 3305 (N-H), 2943 (C-H), 1697 (C=O). **HR-MS**, *m/z* (ES) found M+H$^+$ 409.2880; $C_{22}H_{40}N_2O_3Si$ requires M+H$^+$ 409.2886. **LC-MS**; RT = 0.65-0.70 min, *m/z* (ES) found 409.49.

Tert-butyl N-{2-[(3-bromophenyl)formamido]-2-(3-{[tris(propan-2-yl)silyl]oxy}phenyl)ethyl}carbamate

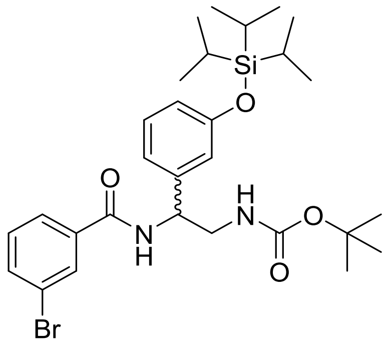

A solution of 3-bromobenzoyl chloride (130 µL, 0.98 mmol), *tert*-butyl *N*-[2-amino-2-(3-{[tris(propan-2-yl)silyl]oxy}phenyl)ethyl]carbamate (390 mg, 0.96 mmol) and triethylamine (280 µL, 1.96 mmol) in anhydrous DCM (20 mL) was stirred at RT for 20 hours. The solvent was stripped *in vacuo* and the residue separated between ethyl acetate (20 mL) and water (20 mL). The organics were washed with brine (20 mL), dried ($MgSO_4$) and the solvent stripped *in vacuo*. The crude product was purified using automated flash column chromatography (0:100 – 20:80 ethyl acetate:petrol ether 40-60°C) to yield the title compound as a colourless solid (370 mg, 0.63 mmol, 64%).

M.p. 84-86 °C. ***R*f** 0.38 (25% ethyl acetate in petrol ether 40-60 °C). **^{1}H NMR (500 MHz, MeOD-*d*$_4$)**; δ 8.03 (app. t, *J* 1.8 Hz, 1H, C2”-H), 7.83 (app. d, *J* 7.8 Hz, 1H, C6”-H), 7.71 (app. ddd, *J* 7.8, 2.0, 1.0 Hz, 1H, C4”-H), 7.40 (app. t, *J* 7.8 Hz, 1H, C5”-H), 7.22 (app. t, *J* 7.8 Hz, 1H, C5’-H), 6.97 (app. d, *J* 7.8 Hz, 1H, C6’-H), 6.90 (app. s, 1H, C2’-H), 6.78 (app. dd, *J* 7.8, 2.0 Hz, 1H, C4’-H), 5.11 (dd, *J* 7.8, 5.5 Hz, 1H, C2-H), 3.41-3.50 (m, 2H, C1-H), 1.42 (s, 9H, ^{t}Bu CH_3), 1.18-1.27 (m, 3H, Si^{i}Pr C-H), 1.05 (dd, *J* 7.5, 3.4 Hz, 18H, Si^{i}Pr CH_3). **^{13}C NMR (125 MHz, MeOD-*d*$_4$)**; δ 166.7 (amide C=O), 158.1 (carbamate C=O), 156.2 (C3’), 141.6 (C1’), 136.3 (C1”), 134.3 (C4”), 130.0 (C2”, C5”), 129.3 (C5’), 125.9 (C6”), 122.1 (C3”), 119.3 (C6’), 118.6 (C4’), 117.6 (C2’), 79.2 ($C(CH_3)_3$), 55.4 (C2), 44.9 (C1), 27.4 (^{t}Bu CH_3), 17.0 (Si^{i}Pr CH_3), 12.5 (Si^{i}Pr C-H). $\bar{\nu}_{max}$ **/cm^{-1}**; 3288 (N-H), 2942 (C-H), 1685 (carbamate C=O), 1637 (amide C=O). **HR-MS**, *m/z* (ES) found M+Na$^+$ 613.2074; $C_{29}H_{43}BrN_2O_4Si$ requires M+Na$^+$ 613.2073. **LC-MS**; RT = 0.95-1.00 min, *m/z* (ES) found 591.48.

Tert-butyl N-{2-[(3-bromophenyl)formamido]-2-(4-{[tris(propan-2-yl)silyl]oxy}phenyl)ethyl}carbamate

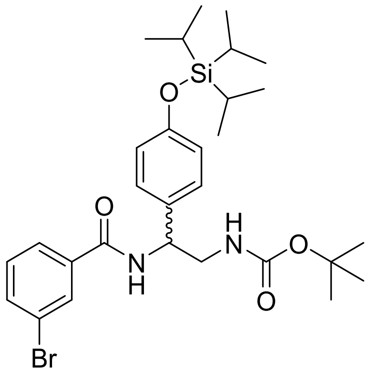

A solution of 3-bromobenzoyl chloride (120 µL, 0.92 mmol), *tert*-butyl *N*-[2-amino-2-(4-{[tris(propan-2-yl)silyl]oxy}phenyl)ethyl]carbamate (350 mg, 0.86 mmol) and triethylamine (250 µL, 1.81 mmol) in anhydrous DCM (20 mL) was stirred at RT for 20 hours. The solvent was stripped *in vacuo* and the residue separated between ethyl acetate (20 mL) and water (20 mL). The organics were washed with brine (20 mL), dried ($MgSO_4$) and the solvent stripped *in vacuo*. Ethyl acetate (5 mL) was added and the solution triturated with petrol ether 40-60°C to yield the title compound as a colourless solid (211 mg, 0.36 mmol, 41%).

M.p. 146-148.5 °C. ***R*f** 0.38 (25% ethyl acetate in petrol ether 40-60 °C). **^{1}H NMR (500 MHz, MeOD-*d*$_4$)**; δ 8.03 (app. t, *J* 1.8 Hz, 1H, C2”-H), 7.83 (app. d, *J* 7.8 Hz, 1H, C6”-H), 7.70 (app. ddd, *J* 7.8, 2.0, 1.0 Hz, 1H, C4”-H), 7.39 (app. t, *J* 7.8 Hz, 1H, C5”-H), 7.27 (app. d, *J* 8.5 Hz, 2H, C2’-H), 6.86 (app. d, *J* 8.5 Hz, 2H, C3’-H), 5.14 (dd, *J* 9.5, 4.8 Hz, 1H, C2-H), 3.49 (dd, *J* 14.3, 9.5 Hz, 1H, CH_2 syn or anti), 3.38 (dd, *J* 14.3, 4.8 Hz, 1H, CH_2 syn or anti), 1.39 (s, 9H, ^{t}Bu CH_3), 1.27 (septet, *J* 7.6 Hz, 3H, Si^{i}Pr C-H), 1.05 (d, *J* 7.6 Hz, 18H, Si^{i}Pr CH_3). **^{13}C NMR (125 MHz, MeOD-*d*$_4$)**; δ 166.8 (amide C=O), 157.8 (carbamate C=O), 155.4 (C4’), 136.4 (C1”), 134.2, (C4”), 132.5 (C1’),

130.1 (C2''), 130.0 (C5''), 127.6 (C2'), 125.9 (C6''), 122.1 (C3''), 119.5 (C3'), 79.0 (<u>C</u>(CH_3)$_3$), 54.7 (C2), 44.9 (C1), 27.3 (^{t}Bu <u>C</u>H_3), 17.0 (Si^{i}Pr <u>C</u>H_3), 12.5 (Si^{i}Pr <u>C</u>-H). $\bar{\nu}_{max}$ **/cm^{-1}**; 3357 (N-H), 2943 (C-H), 1681 (carbamate C=O), 1625 (amide C=O). **HR-MS**, *m/z* (ES) found M+Na$^+$ 613.2068; $C_{29}H_{43}BrN_2O_4Si$ requires M+Na$^+$ 613.2073. **LC-MS**; RT = 0.95-1.00 min, *m/z* (ES) found 1182.63.

Tert-butyl N-(2-{[3-(2-methoxyphenyl)phenyl]formamido}-2-(3-{[tris(propan-2-yl)silyl]oxy}phenyl)ethyl)carbamate

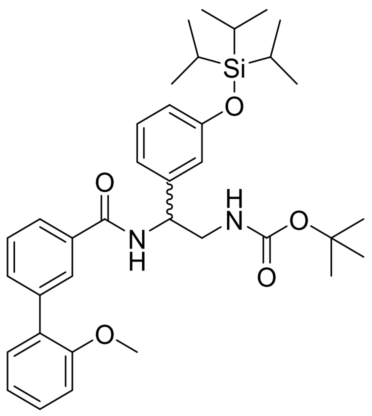

Synthesised *via* Method A. The crude product was purified using automated flash column chromatography (0:100 – 15:85 ethyl acetate:petrol ether 40-60°C) to yield the <u>title compound</u> as a colourless oil (91 mg, 0.15 mmol, 59%).

***R*$_f$** 0.13 (25% ethyl acetate in petrol ether 40-60 °C). **^{1}H NMR (500 MHz, MeOD-*d*$_4$)**; δ 7.97 (app. s, 1H, C2''-<u>H</u>), 7.80 (app. d, *J* 7.8 Hz, 1H, C6''-<u>H</u>), 7.69 (app. dt, *J* 7.8, 1.2 Hz, 1H, C4''-<u>H</u>), 7.48 (app. t, *J* 7.8 Hz, 1H, C5''-<u>H</u>), 7.32-7.38 (m, 2H, C4'''-<u>H</u>, C6'''-<u>H</u>), 7.22 (app. t, *J* 7.8 Hz, 1H, C5'-<u>H</u>), 7.09 (app. d, *J* 8.0 Hz, 1H, C3'''-<u>H</u>), 7.03 (app. td, *J* 7.4, 1.0 Hz, 1H, C5'''-<u>H</u>), 6.98 (app. d, *J* 7.8 Hz, 1H, C6'-<u>H</u>), 6.92 (app. s, 1H, C2'-<u>H</u>), 6.77 (app. dd, *J* 7.8, 1.8 Hz, 1H, C4'-<u>H</u>), 5.13 (dd, *J* 7.6, 5.7 Hz, 1H, C2-<u>H</u>), 3.81 (s, 3H, methoxy-C<u>H</u>$_3$), 3.44-3.47 (m, 2H, C1-<u>H</u>), 1.37 (s, 9H, ^{t}Bu C<u>H</u>$_3$), 1.19-1.25 (m, 3H, Si^{i}Pr C-<u>H</u>), 1.05 (dd, *J* 7.6, 1.3 Hz, 18H, Si^{i}Pr C<u>H</u>$_3$).**^{13}C NMR (125 MHz, MeOD-*d*$_4$)**; δ 168.5 (amide C=O), 158.2 (carbamate C=O), 156.6 (C2'''), 156.2 (C3'), 141.9 (C1'), 139.3 (C1''), 133.8 (C3''), 132.7 (C4''), 130.3 (C4'''), 129.7 (C1'''), 129.2 (C5'), 128.9 (C6'''), 127.9 (C2''), 127.6 (C5''), 125.4 (C6''), 120.5 (C5'''), 119.3 (C6'), 118.5 (C4'), 117.5 (C2'), 111.2 (C3'''), 81.5 (<u>C</u>(CH_3)$_3$), 55.5 (C2), 54.7 (methoxy-<u>C</u>H_3), 44.9 (C1), 27.3 (^{t}Bu <u>C</u>H_3), 17.0 (Si^{i}Pr <u>C</u>H_3), 12.5 (Si^{i}Pr <u>C</u>-H). $\bar{\nu}_{max}$ **/cm^{-1}**; 3345 (N-H), 2942 (C-H), 1684 (carbamate C=O), 1624 (amide C=O). **HR-MS**, *m/z* (ES) found M+Na$^+$ 641.3385; $C_{36}H_{50}N_2O_5Si$ requires M+Na$^+$, 641.3386. **LC-MS**; RT = 1.00-1.05 min, *m/z* (ES) found 519.54.

Tert-butyl N-(2-{[3-(2-methoxyphenyl)phenyl]formamido}-2-(4-{[tris(propan-2-yl)silyl]oxy}phenyl)ethyl)carbamate

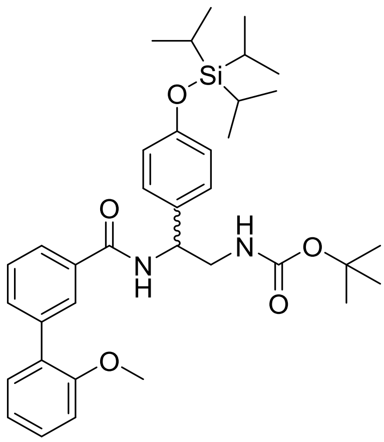

Synthesised *via* Method A. The crude product was purified using automated flash column chromatography (0:100 – 15:85 ethyl acetate:petrol ether 40-60 ˚C) to yield the title compound as a colourless solid (67 mg, 0.11 mmol, 43%).

M.p. 90-92.5 °C. $\boldsymbol{R_f}$ 0.13 (25% ethyl acetate in petrol ether 40-60 ˚C). **^{1}H NMR (500 MHz, MeOD-d_4)**; δ 7.97 (app. s, 1H, C2''-H), 7.79 (app. d, *J* 7.8 Hz, 1H, C6''-H), 7.67 (app. dt, *J* 7.8, 1.3 Hz, 1H, C4''-H), 7.47 (app. t, *J* 7.8 Hz, 1H, C5''-H), 7.32-7.37 (m, 2H, C4'''-H, C6'''-H), 7.28 (app. d, *J* 8.5 Hz, 2H, C2'-H), 7.08 (app. d, *J* 7.8 Hz, 1H, C3'''-H), 7.03 (app. td, *J* 7.5, 1.0 Hz, 1H, C5'''-H), 6.87 (app. d, *J* 8.5 Hz, 2H, C3'-H), 5.15 (dd, *J* 9.2, 4.8 Hz, 1H, C2-H), 3.80 (s, 3H, methoxy-CH_3), 3.49 (dd, *J* 14.3, 9.4 Hz, 1H, CH_2 syn or anti), 3.39 (dd, *J* 14.3, 4.8 Hz, 1H, CH_2 syn or anti), 1.36 (s, 9H, ^{t}Bu CH_3), 1.27 (septet, *J* 7.6 Hz, 3H, Si^{i}Pr C-H), 1.11 (dd, *J* 7.6, 1.3 Hz, 18H, Si^{i}Pr CH_3). **^{13}C NMR (125 MHz, MeOD-d_4)**; δ 168.6 (amide C=O), 157.9 (carbamate C=O), 156.5 (C2'''), 155.3 (C4'), 139.2 (C1'), 133.9 (C1''), 132.8 (C3''), 132.6 (C4''), 130.3 (C4'''), 129.7 (C1'''), 128.9 (C6'''), 128.0 (C2''), 127.6 (C5''), 127.5 (C2'), 125.4 (C6''), 120.6 (C5'''), 119.4 (C3'), 111.2 (C3'''), 79.0 ($C(CH_3)_3$), 54.8 (C2), 54.6 (methoxy-CH_3), 45.0 (C1), 27.3 (^{t}Bu CH_3), 17.0 (Si^{i}Pr CH_3), 12.5 (Si^{i}Pr C-H). $\bar{\nu}_{max}$ **/cm^{-1}**; 3311 (N-H), 2943 (C-H), 1689 (carbamate C=O), 1642 (amide C=O). **HR-MS**, *m/z* (ES) found M+H$^+$ 619.3570; $C_{36}H_{50}N_2O_5Si$ requires M+H$^+$, 619.3567. **LC-MS**; RT = 1.00-1.05 min, *m/z* (ES) found 519.51.

Tert-butyl N-(2-{[3-(3-ethoxy-2-fluorophenyl)phenyl]formamido}-2-(3-{[tris(propan-2-yl)silyl]oxy}phenyl)ethyl)carbamate

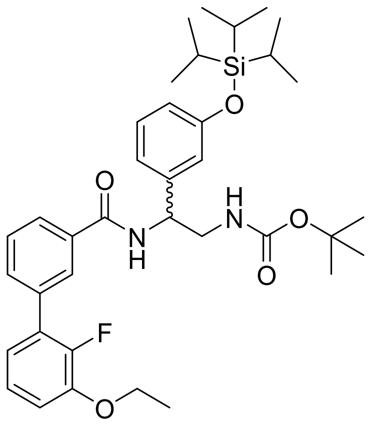

Synthesised *via* Method A. The crude product was purified using automated flash column chromatography (0:100 – 15:85 ethyl acetate:petrol ether 40-60 ˚C) to yield the title compound as a colourless oil (65 mg, 0.10 mmol, 40%).

R_f 0.19 (25% ethyl acetate in petrol ether 40-60 ˚C). **^{1}H NMR (500 MHz, MeOD-*d_4*)**; δ 8.03 (app. s, 1H, C2''-H), 7.87 (app. d, *J* 7.8 Hz, 1H, C6''-H), 7.70-7.74 (m, 1H, C4''-H), 7.56 (app. t, *J* 7.8 Hz, 1H, C5''-H), 7.22 (app. t, *J* 7.8 Hz, 1H, C5'-H), 7.17 (app. td, *J* 8.0, 1.3 Hz, 1H, C5'''-H), 7.11 (app. td, *J* 8.0, 1.7 Hz, 1H, C4'''-H), 7.04-7.08 (m, 1H, C6'''-H), 6.99 (app. d, *J* 7.8 Hz, 1H, C6'-H), 6.92 (app. s, 1H, C2'-H), 6.78 (app. dd, *J* 7.8, 1.9 Hz, 1H, C4'-H), 5.14 (t, *J* 6.5 Hz, 1H, C2-H), 4.16 (q, *J* 7.0 Hz, 2H, ethoxy-CH_2), 3.46 (d, *J* 6.5 Hz, 2H, C1-H), 1.44 (t, *J* 7.0 Hz, 3H, ethoxy-CH_3), 1.39 (s, 9H, ^{t}Bu CH_3), 1.19-1.25 (m, 3H, Si^{i}Pr C-H), 1.05 (dd, *J* 7.6, 1.3 Hz, 18H, Si^{i}Pr CH_3).**^{13}C NMR (125 MHz, MeOD-*d_4*)**; δ 168.1 (amide C=O), 158.1 (C3'), 156.2 (carbamate C=O), 149.6 (d, ^1J_F 246 Hz, C2'''), 147.6 (d, ^2J_F 11.2 Hz, C3'''), 141.8 (C1'), 136.3 (C1''), 134.4 (C3''), 132.0 (C4''), 129.2 (C5'), 128.9 (C1'''), 128.3 (C5''), 127.5 (C2''), 126.3 (C6''), 124.0 (d, ^4J_F 5.0 Hz, C5'''), 121.6 (d, ^3J_F 1.8 Hz, C6'''), 119.3 (C6'), 118.6 (C4'), 117.5 (C2'), 114.0 (C4'''), 79.2 (C(CH$_3$)$_3$), 77.8 (ethoxy-CH$_2$) 64.7 (C2), 45.0 (C1), 27.3 (^{t}Bu CH$_3$), 17.0 (Si^{i}Pr CH$_3$), 13.7 (ethoxy-CH$_3$), 12.5 (Si^{i}Pr C-H). **$\bar{\nu}_{max}$ /cm^{-1} (solid)**; 3296 (N-H), 2941 (C-H), 1684 (carbamate C=O), 1631 (amide C=O). **HR-MS**, *m/z* (ES) found M+H$^+$ 651.3631; $C_{37}H_{51}FN_2O_5Si$ requires M+H$^+$, 651.3629. **LC-MS**; RT = 1.00-1.05 min, *m/z* (ES) found 551.59.

Tert-butyl N-(2-{[3-(3-ethoxy-2-fluorophenyl)phenyl]formamido}-2-(4-{[tris(propan-2-yl)silyl]oxy}phenyl)ethyl)carbamate

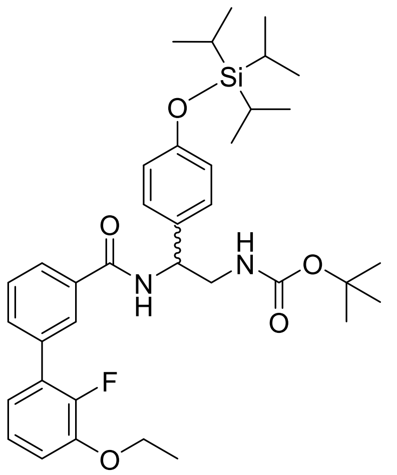

Synthesised *via* Method A. The crude product was purified using automated flash column chromatography (0:100 – 15:85 ethyl acetate:petrol ether 40-60 ˚C) to yield the title compound as a colourless oil (24 mg, 0.04 mmol, 15%).

***R*f** 0.13 (25% ethyl acetate in petrol ether 40-60 ˚C). **^{1}H NMR (500 MHz, MeOD-*d*$_4$)**; δ 8.02 (app. s, 1H, C2”-H), 7.87 (app. d, *J* 7.8 Hz, 1H, C6”-H), 7.70 (app. d, *J* 7.8 Hz, 1H, C4”-H), 7.54 (app. t, *J* 7.8 Hz, 1H, C5”-H), 7.28 (app. d, *J* 8.2 Hz, 2H, C2’-H), 7.16 (app. td, *J* 8.2, 1.4 Hz, 1H, C5’”-H), 7.04-7.12 (m, 2H, C4’”-H, C6’”-H), 6.76 (app. d, *J* 8.2 Hz, 2H, C3’-H), 5.16 (dd, *J* 9.2, 4.6 Hz, 1H, C2-H), 4.15 (q, *J* 7.0 Hz, 2H, ethoxy-CH_2), 3.49 (dd, *J* 14.3, 9.2 Hz, 1H, CH_2 syn or anti), 3.39 (dd, *J* 14.3, 4.6 Hz, 1H, CH_2 syn or anti), 1.44 (t, *J* 7.0 Hz, 3H, ethoxy-CH_3), 1.36 (s, 9H, ^{t}Bu CH_3), 1.22-1.29 (m, 3H, Si^{i}Pr C-H), 1.11 (dd, *J* 7.6, 1.3 Hz, 18H, Si^{i}Pr CH_3). **^{13}C NMR (125 MHz, MeOD-*d*$_4$)**; δ 168.2 (amide C=O), 157.9 (carbamate C=O), 155.3 (C4’), 149.6 (d, $^{1}J_F$ 246 Hz, C2’”), 147.5 (d, $^{2}J_F$ 11.2 Hz, C3’”), 136.2 (C1”), 134.5 (C3”), 132.7 (C1’), 132.0 (C4”), 128.9 (d, $^{2}J_F$ 11.2 Hz, C1’”), 128.3 (C5”), 127.6 (C2”, C2’), 126.3 (C6”), 124.0 (d, $^{4}J_F$ 5.0 Hz, C5’”), 121.6 (d, $^{3}J_F$ 1.8 Hz, C6’”), 119.5 (C3’), 114.0 (d, $^{3}J_F$ 1.8 Hz, C4’”), 79.0 ($C(CH_3)_3$), 77.8 (ethoxy-CH_2) 64.7 (C2), 45.0 (C1), 27.3 (^{t}Bu CH_3), 17.0 (Si^{i}Pr CH_3), 13.7 (ethoxy-CH_3), 12.5 (Si^{i}Pr C-H). **$\bar{\nu}_{max}$ /cm^{-1} (solid)**; ~3300 (N-H), 2942 (C-H), 1687 (carbamate C=O), 1634 (amide C=O). **HR-MS**, *m/z* (ES) found M+H$^+$ 651.3629; $C_{37}H_{51}FN_2O_5Si$ requires M+H$^+$, 651.3629. **LC-MS**; RT = 1.00-1.05 min, *m/z* (ES) found 551.56.

Tert-butyl N-[(2R)-2-phenyl-2-{[2'-(trifluoromethyl)-[1,1'-biphenyl]-3-yl]formamido}ethyl]carbamate

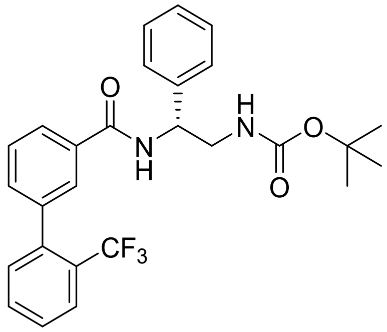

Synthesised *via* Method A. The crude product was purified using automated flash column chromatography (0:100 – 25:75 ethyl acetate:petrol ether 40-60 ˚C) to yield the title compound as a colourless solid (12 mg, 0.02 mmol, 20%).

M.p. 67-69 °C. ***R*** R_f 0.69 (50% ethyl acetate: in petrol ether 40-60 °C). **^{1}H NMR (500 MHz, MeOD-*d*$_4$)**; δ 7.92 (app. dt, *J* 7.4, 1.8 Hz, 1H, C4''-H), 7.83 (s, 1H, C2''-H), 7.80 (app. d, *J* 7.4 Hz, 1H, C6'''-H), 7.67 (app. t, *J* 7.4 Hz, 1H, C4'''-H), 7.58 (app. t, *J* 7.8 Hz, 1H, C5'''-H), 7.48-7.55 (m, 2H, C5''-H, C6''-H), 7.37-7.42 (m, 3H, C3'''-H, C2'-H), 7.34 (app. t, *J* 7.4 Hz, 2H, C3'-H), 7.26 (app. t, *J* 7.4 Hz, 1H, C4'-H), 5.18 (dd, *J* 9.3, 4.7 Hz, 1H, C2-H), 3.50 (dd, *J* 14.3, 9.3 Hz, 1H, CH_2 syn or anti), 3.40 (dd, *J* 14.3, 4.7 Hz, 1H, CH_2 syn or anti), 1.34 (s, 9H, ^{t}Bu CH_3). **^{13}C NMR (125 MHz, MeOD-*d*$_4$)**; δ 168.0 (amide C=O), 158.0 (carbamate C=O), 140.4 (m, C2'''), 140.2 (C3'', C1'''), 140.1 (C1'), 133.8 (C1''), 131.9 (C3''', C4''), 131.6 (C4'''), 128.2 (C3'), 127.8 (C5'''), 127.7 (C5''), 127.6 (C2''), 127.1 (C4'), 126.4 (C6''), 126.3 (C2'), 125.7 (q, ^4J_F 5.3 Hz, C6'''), 124.2 (q ^1J_F 272 Hz, CF_3), 79.1 ($C(CH_3)_3$), 55.7 (C2), 45.0 (C1), 27.3 (^{t}Bu CH_3). **$\bar{\nu}_{max}$ /cm^{-1} (solid)**; 3308 (N-H), 2977 (C-H), 1683 (carbamate C=O), 1643 (amide C=O). **HR-MS**, *m/z* (ES) found M+Na$^+$ 507.1871; $C_{27}H_{27}F_3N_2O_3$ requires M+Na$^+$, 507.1871. **LC-MS**; RT = 0.75-0.80 min, *m/z* (ES) found 385.07.

Tert-butyl N-[(2R)-2-({3'-ethoxy-2'-fluoro-[1,1'-biphenyl]-3-yl}formamido)-2-phenylethyl]carbamate

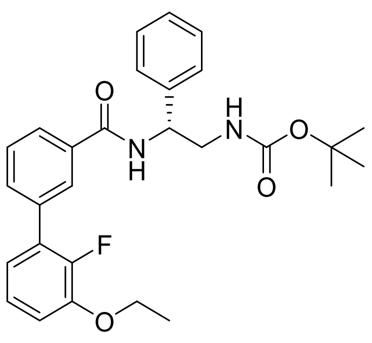

Synthesised *via* Method A. The crude product was purified using automated flash column chromatography (0:100 – 25:75 ethyl acetate:petrol ether 40-60 ˚C) to yield the title compound as a colourless solid (15 mg, 0.03 mmol, 26%).

M.p. 132-133.5 °C. **R_f** 0.64 (50% ethyl acetate in petrol ether 40-60 °C). **^{1}H NMR (500 MHz, MeOD-*d*$_4$)**; δ 8.03 (s, 1H, C2''-H), 7.85-7.89 (m, 1H, C4''-H), 7.71 (app. dd, *J* 7.8, 1.2 Hz, 1H, C6''-H), 7.55 (app. t, *J* 7.8 Hz, 1H, C5''-H), 7.40 (app. d, *J* 7.5 Hz, 2H, C2'-H), 7.35 (app. t, *J* 7.5 Hz, 2H, C3'-H), 7.26 (app. t, *J* 7.5 Hz, 1H, C4'-H), 7.16 (app. td, *J* 8.2, 1.2 Hz, 1H, C5'''-H),

7.09 (app. td, J 7.8, 1.6 Hz, 1H, C4'''-H), 7.04-7.08 (m, 1H, C6'''-H), 5.20 (dd, J 9.0, 4.7 Hz, 1H, C2-H), 4.15 (q, J 7.0 Hz, 2H, ethoxy-CH_2), 3.51 (dd, J 14.2, 9.0 Hz, 1H, CH_2 syn or anti), 3.42 (dd, J 14.2, 4.7 Hz, 1H, CH_2 syn or anti), 1.44 (t, J 7.2 Hz, 3H, ethoxy-CH_3), 1.37 (s, 9H, ^{t}Bu CH_3). **^{13}C NMR (125 MHz, MeOD-*d*$_4$)**; δ 168.2 (amide C=O), 158.0 (carbamate C=O), 149.6 (d, ^1J_F 246 Hz, C2'''), 147.5 (d, ^2J_F 11.2 Hz, C3'''), 140.2 (C1'), 136.3 (C1''), 134.4 (C3''), 132.0 (d, ^4J_F 3.5 Hz, C6''), 128.9 (d, ^2J_F 11.4 Hz, C1'''), 128.3 (C5''), 128.2 (C3'), 127.5 (d, ^4J_F 2.1 Hz, C2''), 127.1 (C4'), 126.4 (C4''), 126.3 (C2'), 124.0 (d, ^4J_F 4.9 Hz, C5'''), 121.6 (d, ^3J_F 1.8 Hz, C6'''), 114.0 (d, ^3J_F 1.6 Hz, C4'''), 79.1 ($C(CH_3)_3$), 64.7 (ethoxy-CH_2), 55.6 (C2), 45.0 (C1), 27.3 (^{t}Bu CH_3), 13.7 (ethoxy-CH_3). **$\bar{\nu}_{max}$ /cm^{-1} (solid)**; 3330 (N-H), 2977 (C-H), 1679 (carbamate C=O), 1632 (amide C=O). **HR-MS**, *m/z* (ES) found M+Na$^+$ 501.2164; $C_{28}H_{31}FN_2O_4$ requires M+Na$^+$, 501.2165. **LC-MS**; RT = 0.75-0.80 min, *m/z* (ES) found 423.06.

Tert-butyl N-[(2R)-2-({3'-ethoxy-4'-fluoro-[1,1'-biphenyl]-3-yl}formamido)-2-phenylethyl]carbamate

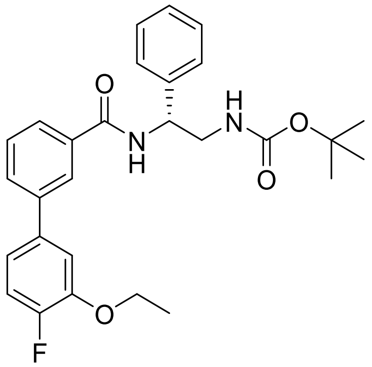

Synthesised *via* Method A. The crude product was purified using automated flash column chromatography (0:100 – 25:75 ethyl acetate:petrol ether 40-60 °C) to yield the title compound as a colourless solid (20 mg, 0.04 mmol, 35%).

M.p. 98.5-100.5 °C. ***R*$_f$** 0.67 (50% ethyl acetate in petrol ether 40-60 °C). **^{1}H NMR (500 MHz, MeOD-*d*$_4$)**; δ 8.08 (s, 1H, C2''-H), 7.83 (app. d, J 7.7 Hz, 1H, C4''-H), 7.75 (app. d, J 7.7 Hz, 1H, C6''-H), 7.52 (app. t, J 7.7 Hz, 1H, C5''-H), 7.41 (app. d, J 7.7 Hz, 2H, C2'-H), 7.32-7.36 (m, 3H, C2'''-H, C3'-H), 7.26 (app. t, J 7.4 Hz, 1H, C4'-H), 7.18-7.22 (m, 1H, C6'''-H), 7.15 (app. dd, J 11.0, 8.5 Hz, 1H, C5'''-H), 5.21 (dd, J 8.9, 4.5 Hz, 1H, C2-H), 4.19 (q, J 7.0 Hz, 2H, ethoxy-CH_2), 3.54 (dd, J 14.2, 8.9 Hz, 1H, CH_2 syn or anti), 3.42 (dd, J 14.2, 5.3 Hz, 1H, CH_2 syn or anti), 1.43 (t, J 7.0 Hz, 3H, ethoxy-CH_3), 1.36 (s, 9H, ^{t}Bu CH_3). **^{13}C NMR (125 MHz, MeOD-*d*$_4$)**; δ 168.3 (amide C=O), 157.9 (carbamate C=O), 152.5 (d, ^1J_F 246 Hz, C4'''), 147.2 (d, ^2J_F 10.9 Hz, C3'''), 140.7 (C1''), 140.2 (C1'), 136.9 (d, ^4J_F 3.6 Hz, C1'''), 134.6 (C3''), 129.8 (C6''), 128.7 (C5''), 128.2 (C3'), 127.1 (C4'), 126.4 (C2'), 125.9 (C4''), 125.6 (C2''), 119.3 (d, ^3J_F 7.1 Hz, C6'''), 118.5 (d, ^2J_F 19.0 Hz, C5''') 113.6 (C2'''), 79.1 ($C(CH_3)_3$), 64.7 (ethoxy-CH_2), 55.7 (C2), 45.0 (C1), 27.3 (^{t}Bu CH_3), 13.8 (ethoxy-CH_3). **$\bar{\nu}_{max}$ /cm^{-1} (solid)**; 3309 (N-H), 2977 (C-H), 1683 (carbamate C=O), 1637 (amide C=O). **HR-MS**, *m/z* (ES) found

M+Na$^+$ 501.2166; $C_{28}H_{31}FN_2O_4$ requires M+Na$^+$, 501.2165. **LC-MS**; RT = 0.75-0.80 min, *m/z* (ES) found 479.14.

Tert-butyl N-[(2R)-2-({3'-hydroxy-[1,1'-biphenyl]-3-yl}formamido)-2-phenylethyl]carbamate

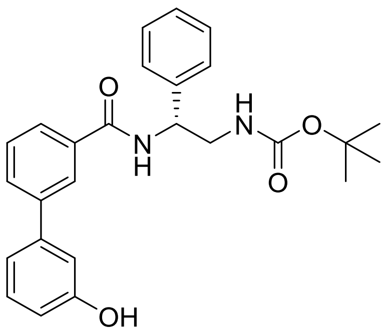

Synthesised *via* Method A. The crude product was purified using automated flash column chromatography (0:100-50:50 ethyl acetate:petroleum ether 40-60 ˚C) to yield as a colourless solid (69 mg, 0.12 mmol, 49%).

M.p. 194.0-196.0 ˚C. ***R*$_f$** 0.47 (50% ethyl acetate in petroleum ether). **^{1}H NMR (MeOD-*d$_4$*);** δ 8.10 (app. s, 1H, C2''-H), 7.83 (app. d, *J* 7.6 Hz, 1H, C6''-H), 7.75-7.77 (m, 1H, C4''-H), 7.53 (app. t, *J* 7.6 Hz, 1H, C5''-H), 7.41 (app. d, *J* 7.6 Hz, 2H, C2'-H,), 7.35 (app. t, *J* 7.6 Hz, 2H, C3'-H,), 7.25-7.28 (m, 2H, C4'-H, C5'''-H), 7.15 (app. d, *J* 7.6 Hz, 1H, C6'''-H), 7.10 (app. s, 1H, C2'''-H), 6.81-6.96 (m, 1H, C4'''-H), 5.20 (dd, *J* 9.1, 4.8 Hz, 1H, C2-H), 3.53 (dd, *J* 14.5, 9.1 Hz, 1H, CH_2 syn or anti), 3.43 (dd, *J* 14.5, 4.8, 1H, CH_2 syn or anti), 1.54 (s, 9H, ^{t}Bu-C'H_3), 1.37 (s, 9H, ^{t}Bu CH_3). **^{13}C NMR (125 MHz, MeOD-*d$_4$*);** δ 168.4 (amide C=O), 157.9 (carbamate C=O), 157.7 (C3'''), 141.6 (C1'), 141.5 (C3''), 140.2 (C1''), 134.5 (C1'''), 129.8 (C4''), 129.6 (C5'''), 128.6 (C5''), 128.2 (C3'), 127.1 (C4'), 126.4 (C2'), 125.8 (C6''), 125.5 (C2''), 118.0 (C6'''), 114.3 (C4'''), 113.6 (C2'''), 79.1 (C-^{t}Bu), 55.5 (C2), 45.0 (C1), 27.3 (^{t}Bu-CH_3). **$\bar{\nu}_{max}$ /cm^{-1} (solid)**; 3398 (N-H), 3274 (O-H), 2990 (C-H), 1673 (carbamate C=O), 1646 (amide C=O). **HR-MS;** *m/z* (ES) found M+Na$^+$ 455.1950; $C_{26}H_{28}N_2O_4$ requires M+Na$^+$ 455.1947 **LC-MS;** RT = 0.70-0.80 min, *m/z* (ES) found 432.46.

Tert-butyl N-[(2R)-2-{[3-(2-chlorophenyl)phenyl]formamido}-2-phenylethyl]carbamate

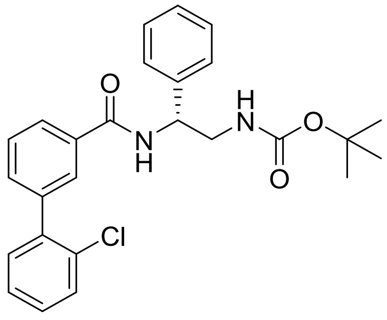

Synthesised *via* Method A. The crude product was purified using automated flash column chromatography (0:100 – 30:70 ethyl acetate:petrol ether 40-60 ˚C) to yield the title compound as a colourless solid (25 mg, 0.06 mmol, 23%).

M.p. 132-134.5 °C. ***R*$_f$** 0.81 (50% ethyl acetate in petrol ether 40-60 ˚C).**^{1}H NMR (500 MHz, MeOD-*d$_4$*)**; δ 7.91 (s, 1H, C2''-H), 7.89 (app. dt, *J*

7.8, 1.2 Hz, 1H, C6''-H), 7.61 (app. dt, *J* 7.8 ,1.3 Hz, 1H, C4''-H), 7.54 (app. t, *J* 7.8 Hz, 1H, C5''-H), 7.50-7.52 (m, 1H, C4'''-H), 7.36-7.43 (m, 5H, C2'-H, C3'''-H, C5'''-H, C6'''-H), 7.34 (app. t, *J* 7.8 Hz, 2H, C3'-H), 7.26 (app. t, *J* 7.2 Hz, 1H, C4'-H), 5.19 (dd, *J* 9.4, 4.6 Hz, 1H, C2-H), 3.50 (dd, *J* 14.2, 9.4 Hz, 1H, CH_2 syn or anti), 3.41 (dd, *J* 14.2, 4.6 Hz, 1H, CH_2 syn or anti), 1.35 (s, 9H, ^{t}Bu CH_3). **^{13}C NMR (125 MHz, MeOD-*d₄*)**; δ 168.2 (amide C=O), 158.0 (carbamate C=O), 140.2 (C1'), 139.8 (C3''), 139.7 (C1'''), 134.1 (C1''), 132.4 (C4''), 132.1 (C2'''), 131.2 (C3'''), 129.6 (C4'''), 128.9 (C6'''), 128.2 (C3'), 128.0 (C2'', C5''), 127.1 (C4'), 126.9 (C5'''), 126.4 (C2'), 126.3 (C6''), 79.1 ($C(CH_3)_3$), 55.6 (C2), 45.0 (C1), 27.3 (^{t}Bu CH_3). **$\bar{\nu}_{max}$ /cm^{-1} (solid)**; 3377 (N-H), 3013 (C-H), 1678 (carbamate C=O), 1632 (amide C=O). **HR-MS**, *m/z* (ES) found M+Na$^+$ 473.1608; $C_{26}H_{27}N_2O_3Cl$ requires M+Na$^+$, 473.1607. **LC-MS**; RT = 0.70-0.75 min, *m/z* (ES) found 351.61.

Tert-butyl N-[(2R)-2-({3'-chloro-[1,1'-biphenyl]-3-yl}formamido)propyl]carbamate

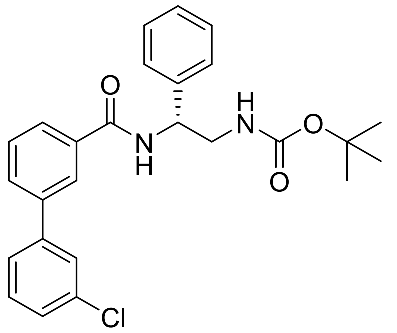

Synthesised *via* Method A. The crude product was purified using automated flash column chromatography (0:100-25:75 ethyl acetate:petroleum ether 40-60 ˚C) to afford a colourless solid (40 mg, 0.09 mmol, 18 %).

M.p. 162.5-163.5 ˚C. ***R*$_f$** 0.60 (25% ethyl acetate in petroleum ether). **^{1}H NMR (MeOD-*d₄*);** δ 8.15 (app. s, 1H, C2''-H), 7.88 (app. d, *J* 7.8 Hz, 1H, C6''-H), 7.78-7.81 (m, 1H, C4''-H), 7.73 (app. s, 1H, C2'''-H), 7.63 (app. d, *J* 7.6 Hz, 1H, C4'''-H), 7.57 (app. t, *J* 7.8 Hz, 1H, C5''-H), 7.46 (app. t, *J* 7.8 Hz, 1H, C5'''-H), 7.41 (app. d, *J* 7.6 Hz, 2H, C2'-H), 7.37-7.39 (m, 1H, C6'''-H), 7.35 (app. t, *J* 7.6 Hz, 2H, C3'-H), 7.26 (app. t, *J* 7.6 Hz, 1H, C4'-H), 5.20 (dd, *J* 9.3, 4.5 Hz, 1H, C2-H), 3.53 (dd, *J* 14.3, 9.3 Hz, 1H, CH_2 syn or anti), 3.43 (dd, *J* 14.3, 4.5 Hz, 1H, CH_2 syn or anti), 1.37 (s, 9H, ^{t}Bu-CH_3). **^{13}C NMR (125 MHz, MeOD-*d₄*);** δ 168.1 (amide C=O), 157.9 (carbamate C=O), 142.2 (C1'), 140.1 (C1'''), 139.9 (C3'''), 134.7 (C1''), 134.5 (C3''), 130.1 (C5''), 129.9 (C4''), 128.9 (C5'''), 128.2 (C3'), 127.3 (C6'''), 127.1 (C4'), 126.8 (C6''), 126.7 (C2'''), 126.4 (C2'), 125.5 (C2''), 125.2 (C4'''), 79.1 (C-^{t}Bu), 55.5 (C2), 45.5 (C1), 27.3 (^{t}Bu-CH_3). **$\bar{\nu}_{max}$ /cm^{-1} (solid)**; 3300 (N-H), 2969 (C-H), 1683 (carbamate C=O), 1627 (amide C=O). **HR-MS;** *m/z* (ES) found M+Na$^+$ 473.1606; $C_{26}H_{27}ClN_2O_3$ requires M+Na$^+$ 431.1607. **LC-MS;** RT = 0.70-0.80 min, *m/z* (ES) found 901.49.

Tert-butyl N-[(2R)-2-({3'-nitro-[1,1'-biphenyl]-3-yl}formamido)-2-phenylethyl]carbamate

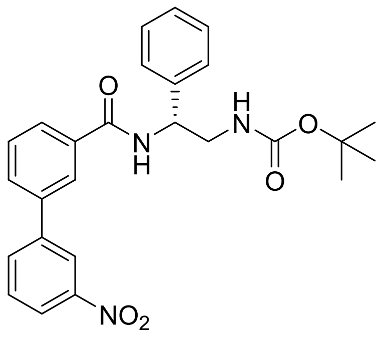

Synthesised *via* Method A. The crude residue was purified using automated flash column chromatography (0:100-30:70 ethyl acetate:petroleum ether 40-60 ˚C) to yield a colourless solid (40 mg, 0.08 mmol, 36%).

M.p. 174.5-176.0 ˚C. ***R*f** 0.48 (50% ethyl acetate in petroleum ether). **^{1}H NMR (MeOD-*d$_4$*);** δ 8.57 (app. s, 1H, C2'''-H), 8.25-8.27 (m, 1H, C4'''-H), 8.23 (app. s, 1H, C2''-H), 8.13 (app. d, 1H, C6'''-H), 7.94 (app. d, *J* 7.7 Hz, 1H, C4''-H), 7.89-7.92 (m, 1H, C6''-H), 7.73 (app. t, *J* 7.7 Hz, 1H, C5'''-H), 7.62 (app. t, *J* 7.7 Hz, 1H, C5''-H), 7.42 (app. d, *J* 7.7 Hz, C2'-H), 7.36 (app. t, *J* 7.7 Hz, 2H, C3'-H), 7.26 (app. t, *J* 7.7 Hz, 1H, C4'-H), 5.22 (dd, *J* 9.2, 4.5 Hz, 1H, C2-H), 3.55 (dd, *J* 14.2, 9.2 Hz, 1H, CH_2 syn or anti), 3.45 (dd, *J* 14.2, 4.5, 1H, CH_2 syn or anti), 1.54 (s, 9H, ^{t}Bu-C'H_3). **^{13}C NMR (125 MHz, MeOD-*d$_4$*);** δ 167.9 (amide C=O), 157.9 (carbamate C=O), 148.9 (C3'''), 141.9 (C1'), 140.1 (C3''), 139.0 (C1'''), 135.0 (C1''), 132.9 (C6'''), 130.0 (C6''), 129.9 (C5'''), 129.2 (C5''), 128.2 (C3'), 127.2 (C4''), 127.1 (C4'), 126.4 (C2'), 125.8 (C2''), 122.1 (C4'''), 121.4 (C2'''), 79.0 (C-^{t}Bu), 55.6 (C2), 45.0 (C1), 27.2 (^{t}Bu-CH_3). $\bar{\nu}_{max}$ **/cm^{-1} (solid)**; 3365 (N-H), 2934 (C-H), 1634 (amide C=O), 1583 (nitro NO_2). **HR-MS;** *m/z* (ES) found M+Na$^+$ 484.1848; $C_{26}H_{27}N_3O_5$ requires M+Na$^+$ 474.1056. **LC-MS;** RT = 0.70-0.80 min, *m/z* (ES) found 374.34.

Tert-butyl N-[(2R)-2-({3'-acetyl-[1,1'-biphenyl]-3-yl}formamido)-2-phenylethyl]carbamate

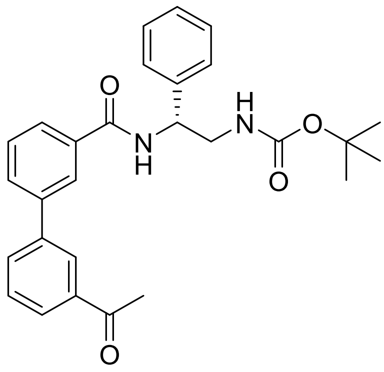

Synthesised *via* Method A. The crude product was purified using automated flash column chromatography (0:100-30:70 ethyl acetate:petroleum ether 40-60 ˚C) to yield a colourless solid (63 mg, 0.08 mmol, 37%).

M.p. 158.5-160.0 ˚C. ***R*f** 0.46 (25% ethyl acetate in petroleum ether). **^{1}H NMR (MeOD-*d$_4$*);** δ 8.30 (app. s, 1H, C2'''-H), 8.19 (app. s, 1H, C2''-H), 8.01-8.04 (m, 1H, C6'''-H), 7.95 (app. d, *J* 7.8 Hz, 1H, C4'''-H), 7.90 (app. d, *J* 7.8 Hz, 1H, C5'''-H), 7.85-7.88 (m, 1H, C5''-H), 7.57-7.63 (m, 2H, C4''-H, C6''-H), 7.42 (app. d, *J* 7.5 Hz, 2H, C2'-

H), 7.36 (app. t, *J* 7.5 Hz, 2H, C3'-H), 7.27 (app. t, *J* 7.5 Hz, 1H, C4'-H), 5.21 (dd, *J* 9.4, 4.6 Hz, 1H, C2-H), 3.54 (dd, *J* 14.3, 9.4 Hz, 1H, CH_2 syn or anti), 3.43 (dd, *J* 14.3, 4.6 Hz, 1H, CH_2 syn or anti), 2.68 (s, 3H, CO-CH_3), 1.36 (s, 9H, ^{t}Bu-CH_3). **^{13}C NMR (125 MHz, MeOD-*d*$_4$);** δ 198.9 (ketone C=O), 168.2 (amide C=O), 157.9 (carbamate C=O), 140.7 (C1'), 140.4 (C3''), 140.2 (C1'''), 137.7 (C1''), 134.8 (C3'''), 131.6 (C4'''), 129.9 (C5''), 129.0 (C6''), 128.9 (C4''), 128.2 (C3'), 127.4 (C6'''), 127.1 (C4'), 126.6 (C2'''), 126.5 (C5'''), 126.4 (C2'), 125.7 (C2''), 79.1 (C-^{t}Bu), 55.5 (C2), 45.0 (C1), 27.3 (^{t}Bu-CH$_3$), 25.5 (ketone-CH$_3$). $\bar{\nu}_{max}$ **/cm^{-1} (solid)**; 3365 (N-H), 2969 (C-H), 1680 (amide C=O), 1659 (carbamate C=O), 1599 (ketone C=O). **HR-MS;** *m/z* (ES) found M+Na$^+$ 481.2101; $C_{28}H_{30}N_2O_4$ requires M+Na$^+$ 481.5397. **LC-MS;** RT = 0.60-0.70 min, *m/z* (ES) found 917.42.

Methyl (R)-3'-{[2-{(tert-butoxycarbonyl)amino}-1-phenylethyl]carbamoyl}-[1,1'-biphenyl]-3-carboxylate

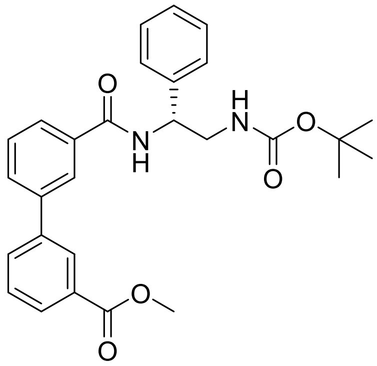

Synthesised *via* Method A. The crude product was purified using automated flash column chromatography on silica gel (0:1 – 1:1 MeOH:DCM) to yield the title compound as a colourless solid (48 mg, 0.10 mmol, 53%).

M.p. 140-142 °C. ***R*$_f$** 0.50 (20% EtOAc in hexane). **^{1}H NMR (500 MHz, MeOD-*d*$_4$)**; δ 8.34 (t, *J* 1.8 Hz, 1H, C2'''-H), 8.18 (t, *J* 1.8 Hz, 1H, C2''-H), 8.04 (dt, *J* 7.8, 1.3 Hz, 1H, C4'''-H), 7.96 (dt, *J* 8.0, 1.4 Hz, 1H, C6''-H), 7.90 (dt, *J* 7.8, 1.4 Hz, 1H, C6'''-H), 7.85 (ddd, *J* 7.8, 1.9, 1.1 Hz, 1H, C4''-H), 7.60 (td, *J* 7.8, 4.0 Hz, 2H, C5''-H, C5'''-H), 7.40-7.45 (m, 2H, C2'-H), 7.36 (t, *J* 7.7 Hz, 2H, C3'-H), 7.23-7.30 (m, 1H, C4'-H), 5.22 (dd, *J* 9.2, 4.6 Hz, 1H, C1-H), 3.95 (s, 3H, ester CH_3), 3.55 (dd, *J* 14.2, 9.2 Hz, 1H, C2-H syn or anti), 3.44 (dd, *J* 14.2, 4.7 Hz, 1H, C2-H syn or anti), 1.37 (s, 9H, ^{t}Bu CH_3). **^{13}C NMR (125 MHz, MeOD-*d*$_4$)**; δ 168.2 (amide C=O), 166.9 (ester C=O), 157.9 (carbamate C=O), 140.7 (C3''/C1'''), 140.4 (C3''/C1'''), 140.2 (C1'), 134.9 (C1''), 131.5 (C6''), 130.8 (C3'''), 129.9 (C4''), 129.0 (C5'', C5'''), 128.4 (C4'''), 128.2 (C3'), 127.7 (C2'''), 127.1 (C4'), 126.5 (C6'''), 126.4 (C2'), 125.7 (C2''), 79.1 (C(CH$_3$)$_3$), 55.6 (C1), 51.4 (ester CH$_3$), 45.0 (C2), 27.3 (^{t}Bu CH$_3$). $\bar{\nu}_{max}$ **/cm^{-1} (solid)**; 3306 (N-H), 2977 (C-H), 1680-1642 (ester C=O, amide C=O, carbamate C=O). **HR-MS**, *m/z* (ES) found M+H$^+$ 475.2227,

$C_{28}H_{30}N_2O_5$ requires M+H$^+$ 475.2227. **LC-MS**; RT = 0.70-0.75 min, *m/z* (ES) found 497.16.

Methyl (R)-3'-{[2-{(tert-butoxycarbonyl)amino}-1-phenylethyl]carbamoyl}-[1,1'-biphenyl]-2-carboxylate

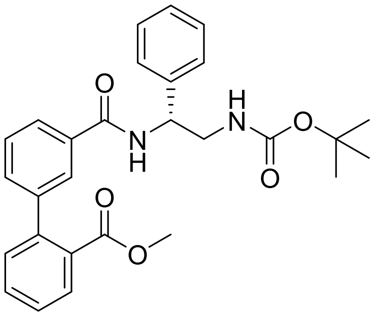

Synthesised *via* Method A. The crude product was purified using automated flash column chromatography on silica gel (0:1 – 1:1 MeOH:DCM) to yield the title compound as a colourless solid (12 mg, 0.03 mmol, 13%).

M.p. 77-79.5 °C. R_f 0.50 (20% EtOAc in hexane). **^{1}H NMR (500 MHz, MeOD-*d*$_4$)**; δ 7.82-7.88 (m, 3H, C2”-H, C6”-H, C4”-H), 7.61 (td, *J* 7.5, 1.4 Hz, 1H, C3’”-H), 7.47-7.53 (m, 2H, C5”-H, C5’”-H), 7.42-7.48 (m, 2H, C4’”-H. C6’”-H), 7.38-7.41 (m, 2H, C2’-H), 7.35 (t, *J* 7.7 Hz, 2H, C3’-H), 7.22-7.30 (m, 1H, C4’-H), 5.19 (dd, *J* 9.0, 4.7 Hz, 1H, C1-H), 3.61 (s, 3H. ester CH_3), 3.46-3.55 (m, 1H, C2-H syn or anti), 3.42 (dd, *J* 14.3, 4.8 Hz, 1H, C2-H syn or anti), 1.36 (s, 9H, ^{t}Bu CH_3). **^{13}C NMR (125 MHz, MeOD-*d*$_4$)**; δ 169.0 (ester C=O), 168.2 (amide C=O), 157.9 (carbamate C=O), 141.7 (C3”/C1’”), 141.6 (C3”/C1’”), 140.2 (C1’), 134.1 (C1”), 131.4 (C6’”), 131.3 (C3’”), 130.7 (C2’”), 130.5 (C4’”), 129.6 (C4”), 128.2 (C3’), 127.9 (C5’”/C5”), 127.4 (C5’”/C5”), 127.1 (C4’), 127.0 (C6”), 126.4 (C2’), 125.9 (C2”), 79.1 ($C(CH_3)_3$), 55.6 (C1), 51.1 (ester CH_3), 45.0 (C2), 27.3 (^{t}Bu CH_3). **$\bar{\nu}_{max}$ /cm^{-1} (solid)**; 3360 (N-H), 2983 (C-H), 1694-1671 (ester C=O, amide C=O, carbamate C=O). **HR-MS**, *m/z* (ES) found M+H$^+$ 475.2232, $C_{28}H_{30}N_2O_5$ requires M+H$^+$ 475.2227. **LC-MS**; RT = 0.70-0.75 min, *m/z* (ES) found 497.15.

Tert-butyl (2-(2-methylbenzamido)-2-phenylethyl)carbamate

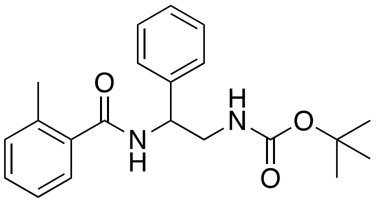

19 (84 mg, 0.36 mmol) was dissolved in DCM (10 mL). Triethylamine (100 µL, 0.72 mmol) was added to the mixture followed by *o*-toluoyl chloride (47 µL, 0.36 mmol). The mixture was stirred at RT for 16 hours. The solvent was stripped *in vacuo* and the crude separated between DCM (20 mL) and water (20 mL). The organic layer was washed with water (20 mL), $NaHCO_{3\,(aq)}$ (20 mL) and brine (20 mL) before being dried ($MgSO_4$). The crude material was purified by flash column

chromatography (1:3 ethyl acetate:hexane) to yield the title compound as a colourless solid (80 mg, 0.23 mmol, 63%).

R$_f$ 0.19 (25% ethyl acetate in hexane). **^{1}H NMR** (400 MHz, $CDCl_3$) δ 7.34 (d, *J* 7.59 Hz, C2''-H), 7.29 – 7.28 (m, 4H, C2'-H, C4''-H, C5''-H), 7.23 – 7.19 (m, 2H, C3'-H), 7.12 – 7.07 (m, 2H, C4'-H, C3''-H), 5.17 – 5.05 (m, 1H, C2-H), 3.52 (dt, *J* 15.4, 6.2 Hz, 1H, CH_2 syn or anti), 3.33 (dt, *J* 14.6, 4.8 Hz, 1H, CH_2 syn or anti), 2.31 (s, 3H, $PhCH_3$), 1.30 (s, 9H, ^{t}Bu CH_3). **^{13}C NMR** (101 MHz, $CDCl_3$) δ 170.1 (amide C=O), 157.3 (carbamate C=O), 139.9 (C1'), 136.5 (C6''), 136.0 (C1''), 131.0, (C4'') 130.0 (C5''), 128.8 (C3'), 127.7 (C2''), 127.0 (C3''), 126.6 (C2'), 125.7 (C4'), 80.0 ($C(CH_3)_3$), 70.0 (C2), 45.7 (C1), 28.4 (^{t}Bu CH_3), 20.0 ($PhCH_3$). **$\bar{\nu}_{max}$ /cm^{-1} (solid)**; 3358 (N-H), 3028 (C-H), 1685 (carbamate C=O), 1631 (amide C=O). **HR-MS**; *m/z* (ES) found M+Na$^+$ 377.1846, $C_{21}H_{26}N_2O_3$ requires M+Na$^+$ 377.1840. **HPLC**; 3.2 min, 92.6%.

Tert-butyl (2-(3-methylbenzamido)-2-phenylethyl)carbamate

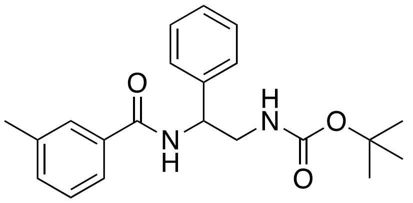

19 (150 mg, 0.64 mmol) was dissolved in DCM (10 mL). Triethylamine (178 µL, 1.28 mmol) was added to the mixture followed by *m*-toluoyl chloride (84 µL, 0.64 mmol). The mixture was stirred at RT for 16 hours. The solvent was stripped *in vacuo* and the crude separated between ethyl acetate (20 mL) and water (20 mL). The organic layer was washed with water (20 mL) and brine (20 mL) before being dried ($MgSO_4$). The crude material was purified by flash column chromatography (1:1 ethyl acetate:hexane) to yield the title compound as a colourless solid (40 mg, 0.11 mmol, 18%).

R$_f$ 0.94 (50% ethyl acetate in hexane). **^{1}H NMR** (400 MHz, $CDCl_3$) δ 7.88 – 7.84 (m, 2H, C2''-H, C6''-H), 7.70 (t, *J* 11.9 Hz, 2H, C3''-H, C4''-H), 7.37 – 7.25 (m, 5H, C2'-4'-H), 5.16 (s, 1H, C2-H), 3.64 (dd, *J* 15.5, 9.3 Hz, 1H, CH_2 syn or anti), 3.47 (d, *J* 8.5 Hz, 1H, CH_2 syn or anti), 2.39 (s, 3H, $PhCH_3$), 1.42 (s, 9H, ^{t}Bu CH_3). **^{13}C NMR** (101 MHz, MeOD-*d$_4$)* δ 166.4 (amide C=O), 158.8 (carbamate C=O), 141.6 (C1'), 139.5 (C5''), 135.4 (C1''), 133.4 (C4''), 129.6 (C3'), 129.5 (C6''), 128.9 (C3''), 128.5 (C4'), 127.7 (C2'), 125.6 (C2''), 80.5 ($C(CH_3)_3$), 56.8 (C2), 46.3 (C1), 28.7 (^{t}Bu CH_3), 21.4 ($PhCH_3$). **HR-MS**; *m/z* (ES) found M+H$^+$ 355.2016; $C_{21}H_{26}N_2O_3$ requires M+H$^+$ 355.2022. **HPLC**; 3.40 min, 95.0%.

Tert-butyl (R)-(2-(4-methylbenzamido)-2-phenylethyl)carbamate

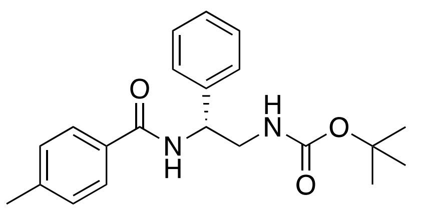

19 (100 mg, 0.42 mmol) was dissolved in DCM (10 mL). Triethylamine (117 µL, 0.84 mmol) was added to the mixture followed by *p*-toluoyl chloride (56 µL, 0.42 mmol). The mixture was stirred at RT for 12 hours. The solvent was stripped *in vacuo* and the crude separated between DCM (20 mL) and water (20 mL). The organic layer was washed with water (20 mL), $NaHCO_{3\,(aq)}$ (20 mL) and brine (20 mL) before being dried ($MgSO_4$). The crude material was purified by flash column chromatography (1:9 – 1:4 ethyl acetate:hexane) to yield the title compound as a colourless solid (85 mg, 0.24 mmol, 57%).

R_f 0.19 (25% ethyl acetate in hexane). **^{1}H NMR** (400 MHz, $CDCl_3$) δ 7.92 (d, *J* 6.5 Hz, 1H, amide N-H), 7.71 (d, *J* 7.8 Hz, 2H, C2''-H), 7.27 – 7.23 (m, 3H, C2'-H, C4'-H), 7.25 – 7.15 (m, 2H, C3'-H), 7.13 (d, *J* 7.8 Hz, 2H, C3''-H), 5.19 – 5.15 (m, 1H, carbamate N-H), 5.11 – 5.06 (m, 1H, C1-H), 3.54 (dt, *J* 16.4, 8.7 Hz, 1H, CH_2 syn or anti), 3.34 (dt, *J* 15.5, 4.6 Hz, 1H, CH_2 syn or anti), 2.31 (s, 3H, $PhCH_3$), 1.35 (s, 9H, ^{t}Bu CH_3). **^{13}C NMR** (101 MHz, $CDCl_3$) δ 167.1 (amide C=O), 158.1 (carbamate C=O), 141.9 (C4'), 140.1 (C1'), 131.2 (C1''), 129.1 (C3''), 128.7 (C3'), 127.6 (C4'), 127.3 (C2''), 126.5 (C2'), 80.2 ($C(CH_3)_3$), 56.7 (C2), 45.8 (C1), 28.4 (^{t}Bu CH_3), 21.5 ($PhCH_3$). **HRMS**; m/z (ES) found M+Na$^+$ 377.1836, $C_{21}H_{26}N_2O_3$ requires M+Na$^+$ 377.1840. **HPLC**; 3.4 min, 99.4%.

Tert-butyl N-[(2R)-2-({2'-methoxy-6-methyl-[1,1'-biphenyl]-3-yl}formamido)-2-phenylethyl]carbamate

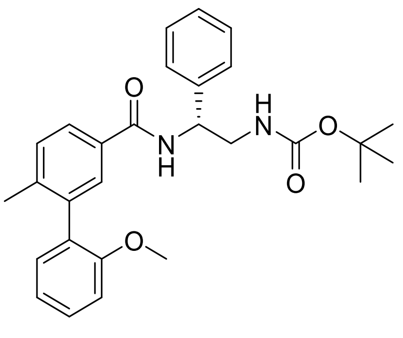

Synthesised *via* Method A. The crude product was purified using automated flash column chromatography (0:100 – 25:75 ethyl acetate:petrol ether 40-60 ˚C) to yield the title compound as an off-white solid (15 mg, 0.03 mmol, 27%).

M.p. 85.5-87 °C. ***R_f*** 0.63 (50% ethyl acetate in petrol ether 40-60 °C). **^{1}H NMR (500 MHz, MeOD-*d_4*)**; δ 7.74 (app. dd, *J* 8.0, 2.0 Hz, 1H, C4''-H), 7.64 (app. d, *J* 2.0 Hz, 1H, C2''-H), 7.35-7.39 (m, 3H, C2'-H, C4'''-H), 7.30-7.35 (m, 3H, C3'-H, C5''-H), 7.24 (app. t, *J* 7.5 Hz, 1H, C4'-H), 7.12 (app. d, *J* 7.1 Hz, 1H, C6'''-H), 7.05 (app. d, *J* 8.0 Hz, 1H, C3'''-H), 7.01 (app. t, *J* 7.5 Hz, 1H, C5'''-H), 5.15

(dd, *J* 9.1, 4.8 Hz, 1H, C2-H), 3.73 (s, 3H, methoxy-CH_3), 3.47 (dd, *J* 14.3, 9.1 Hz, 1H, CH_2 syn or anti), 3.38 (dd, *J* 14.3, 4.8 Hz, 1H, CH_2 syn or anti), 2.13 (s, 3H, C4”-CH_3), 1.35 (s, 9H, ^{t}Bu CH_3). **^{13}C NMR (125 MHz, MeOD-*d*$_4$)**; δ 168.5 (amide C=O), 158.0 (C2’’’), 156.6 (carbamate C=O), 141.1 (C1”), 140.3 (C1’), 139.2 (C6”), 131.2 (C3”), 130.4 (C6’’’), 129.9 (C1’’’), 129.3 (C5”), 128.9 (C4’’’), 128.5 (C2”), 128.2 (C3’), 127.0 (C4’), 126.3 (C2’), 125.9 (C4”), 120.3 (C5’’’), 110.6 (C3’’’), 79.1 ($C(CH_3)_3$), 55.6 (C2), 54.4 (methoxy-CH_3), 45.0 (C1), 27.3 (^{t}Bu CH_3), 18.7 (C4”-CH_3). $\bar{\nu}_{max}$ **/cm^{-1} (solid)**; 3306 (N-H), 2972 (C-H), 1684 (carbamate C=O), 1639 (amide C=O). **HR-MS**, *m/z* (ES) found M+Na^+ 483.2260; $C_{28}H_{32}N_2O_4$ requires M+Na^+, 483.2254. **LC-MS**; RT = 0.70-0.75 min, *m/z* (ES) found 461.13.

Tert-butyl N-[(2R)-2-({3'-ethoxy-6-methyl-[1,1'-biphenyl]-3-yl}formamido)-2-phenylethyl]carbamate

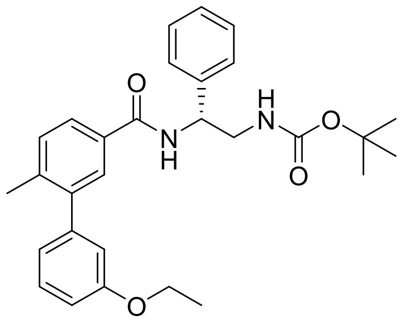

Synthesised *via* Method A. The crude product was purified using automated flash column chromatography (0:100 – 25:75 ethyl acetate:petrol ether 40-60 ˚C) to yield the title compound as an off-white solid (16 mg, 0.03 mmol, 28%).

M.p. 64-66 °C. ***R*$_f$** 0.71 (50% ethyl acetate in petrol ether 40-60 °C). **^{1}H NMR (500 MHz, MeOD-*d*$_4$)**; δ 7.74 (app. dd, *J* 8.2, 2.1 Hz, 1H, C4”-H), 7.73 (app. s, 1H, C2”-H), 7.34-7.38 (m, 3H, C2’-H, C5”-H), 7.30-7.34 (m, 3H, C3’-H, C5’’’-H), 7.24 (app.t , *J* 7.5 Hz, 1H, C4’-H), 6.90-6.93 (m, 1H, C6’’’-H), 6.87 (app. d, *J* 7.8 Hz, 1H, C4’’’-H), 6.86 (app. d, *J* 2.1 Hz, 1H, C2’’’-H), 5.16 (dd, *J* 9.3, 4.6 Hz, 1H, C2-H), 4.06 (q, *J* 7.0 Hz, 2H, ethoxy-CH_2), 3.48 (dd, *J* 14.2, 9.3 Hz, 1H, CH_2 syn or anti), 3.39 (dd, *J* 14.2, 4.6 Hz, 1H, CH_2 syn or anti), 2.29 (s, 3H, C4”-CH_3), 1.39 (t, *J* 7.0 Hz, 3H, ethoxy-CH_3), 1.35 (s, 9H, ^{t}Bu CH_3). **^{13}C NMR (125 MHz, MeOD-*d*$_4$)**; δ 168.3 (amide C=O), 159.0 (C3’’’), 158.0 (carbamate C=O), 142.4 (C1”), 142.1 (C1’’’), 140.3 (C1’), 139.4 (C6”), 131.5 (C3”), 130.2 (C5”), 129.0 (C5’’’), 128.2 (C3’), 128.1 (C2”), 127.1 (C4’), 126.3 (C2’), 126.0 (C4”), 121.1 (C4’’’), 115.1 (C2’’’), 113.0 (C6’’’), 79.1 ($C(CH_3)_3$), 63.1 (ethoxy-CH_2), 55.6 (C2), 45.0 (C1), 27.3 (^{t}Bu CH_3), 19.2 (C4”-CH_3), 13.8 (ethoxy-CH_3). $\bar{\nu}_{max}$ **/cm^{-1} (solid)**; 3318 (N-H), 2976 (C-H), 1685 (carbamate C=O), 1638 (amide C=O). **HR-MS**, *m/z* (ES) found M+Na^+ 497.2409; $C_{29}H_{34}N_2O_4$ requires M+Na^+, 497.2416. **LC-MS**; RT = 0.75-0.80 min, *m/z* (ES) found 475.17.

Tert-butyl N-[(2R)-2-({3'-ethoxy-5',6-dimethyl-[1,1'-biphenyl]-3-yl}formamido)-2-phenylethyl]carbamate

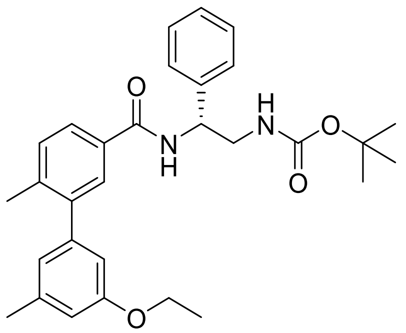

Synthesised *via* Method A. The crude product was purified using automated flash column chromatography (0:100 – 25:75 ethyl acetate:petrol ether 40-60 °C) to yield the title compound as a colourless solid (15 mg, 0.03 mmol, 26%).

M.p. 75-76 °C. $\boldsymbol{R_f}$ 0.77 (50% ethyl acetate in petrol ether 40-60 °C). **^{1}H NMR (500 MHz, MeOD-*d$_4$*)**; δ 7.73 (app. dd, *J* 8.0, 2.3 Hz, 1H, C6''-H), 7.71 (app. s, 1H, C2''-H), 7.38 (app. d, *J* 7.5 Hz, 2H, C2'-H), 7.30-7.35 (m, 3H, C3'-H, C5''-H), 7.24 (app. t, *J* 7.5 Hz, 1H, C4'-H), 6.74 (app. s, 1H, C4'''-H), 6.70 (app. s, 1H, C6'''-H), 6.65 (app. s, 1H, C2'''-H), 5.15 (dd, *J* 8.9, 4.6 Hz, 1H, C2-H), 4.03 (q, *J* 7.0 Hz, 2H, ethoxy-CH_2), 3.48 (dd, *J* 14.1, 8.9 Hz, 1H, CH_2 syn or anti), 3.39 (dd, *J* 14.1, 4.6 Hz, 1H, CH_2 syn or anti), 2.35 (s, 3H, C5'''-CH_3), 2.28 (s, 3H, C4''-CH_3), 1.38 (t, *J* 7.0 Hz, 3H, ethoxy-CH_3), 1.35 (s, 9H, ^{t}Bu CH_3). **^{13}C NMR (125 MHz, MeOD-*d$_4$*)**; δ 168.3 (amide C=O), 158.9 (C3'''), 158.0 (carbamate C=O), 142.3 (C1''), 142.2 (C1'''), 140.2 (C1'), 139.4 (C6''), 139.1 (C5'''), 131.6 (C3''), 130.1 (C5''), 128.2 (C3'), 128.0 (C2''), 127.1 (C4'), 126.3 (C2'), 125.9 (C4''), 121.9 (C6'''), 113.7 (C4'''), 112.2 (C2'''), 79.1 ($C(CH_3)_3$), 63.1 (ethoxy-CH_2), 55.7 (C2), 45.0 (C1), 27.3 (^{t}Bu CH_3), 20.3 (C5'''-CH_3), 19.2 (C4''-CH_3), 13.8 (ethoxy-CH_3). $\bar{\nu}_{max}$ **/cm^{-1} (solid)**; 3303 (N-H), 2976 (C-H), 1685 (carbamate C=O), 1638 (amide C=O). **HR-MS**, *m/z* (ES) found M+Na$^+$ 511.2569; $C_{30}H_{36}N_2O_4$ requires M+Na$^+$, 511.2572. **LC-MS**; RT = 0.75-0.80 min, *m/z* (ES) found 489.16.

Tert-butyl N-[(2S)-2-phenyl-2-[(3-phenylphenyl)formamido]ethyl]carbamate

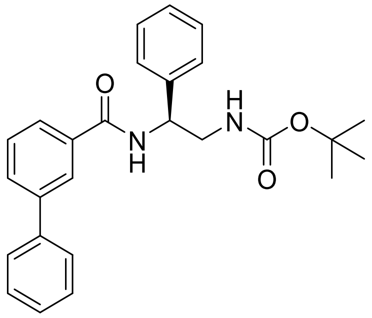

Synthesised *via* Method A. The crude product was purified using automatic flash column chromatography (0-25% ethyl acetate in petrol ether 40-60 °C) to yield the title compound as a colourless solid (25 mg, 0.06 mmol, 24%).

M.p. 156-158.5 °C. $\boldsymbol{R_f}$ 0.85 (50% ethyl acetate in petrol ether 40-60 °C). **^{1}H NMR (500 MHz, MeOD-*d$_4$*)**; δ 8.15 (s, 1H, C2''-H), 7.85 (app. d, *J* 7.7 Hz, 1H, C4''-H), 7.81 (app. d, *J* 7.7 Hz, 1H, C6''-H), 7.70 (app. d, *J* 7.4 Hz, 2H, C2'''-H), 7.55 (app. t, *J* 7.7 Hz, 1H, C5''-H), 7.47 (app. t, *J*

7.4 Hz, 2H, C3'''-H), 7.42 (app. d, *J* 7.5 Hz, 2H, C2'-H), 7.33-7.40 (m, 3H, C3'-H, C4'''-H), 7.27 (app. t, *J* 7.5 Hz, 1H, C4'-H), 5.21 (dd, *J* 9.1, 4.6 Hz, 1H, C2-H), 3.53 (dd, *J* 14.3, 9.1 Hz, 1H, CH_2 syn or anti), 3.44 (dd, *J* 14.3, 4.6 Hz, 1H, CH_2 syn or anti), 1.38 (s, 9H, ^{t}Bu CH_3). **^{13}C NMR (125 MHz, MeOD-*d*$_4$)**; δ 168.4 (amide C=O), 158.0 (carbamate C=O), 141.5 (C3''), 140.2 (C1'), 140.1 (C1'''), 134.6 (C1''), 129.9 (C6'') 128.7 (C5''), 128.6 (C3'''), 128.2 (C3'), 127.4 (C4'''), 127.1 (C4'), 126.8 (C2'''), 126.4 (C2'), 125.9 (C4''), 125.5 (C2''), 79.1 ($C(CH_3)_3$), 55.6 (C2), 45.0 (C1), 27.3 (^{t}Bu CH_3). **$\bar{\nu}_{max}$ /cm^{-1} (solid)**; 3031 (C-H), 1675 (carbamate C=O), 1626 (amide C=O). **HR-MS**, *m/z* (ES) found M+Na$^+$ 439.1993; $C_{26}H_{28}N_2O_3$ requires M+Na$^+$, 439.1997. **LC-MS**; RT = 0.70-0.75 min, *m/z* (ES) found 316.96.

Tert-butyl N-[(2S)-2-{[3-(2-methoxyphenyl)phenyl]formamido}-2-phenylethyl]carbamate

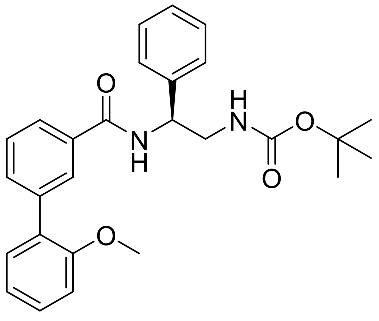

Synthesised *via* Method A. The crude product was purified using automatic flash column chromatography (0-25% ethyl acetate in petrol ether 40-60 ˚C) to yield the title compound as a colourless solid (25 mg, 0.06 mmol, 22%).

M.p. 91-93 °C. ***R*$_f$** 0.74 (50% ethyl acetate in petrol ether 40-60 ˚C). **^{1}H NMR (500 MHz, MeOD-*d*$_4$)**; δ 7.98 (s, 1H, C2''-H), 7.80 (app. d, *J* 7.8 Hz, 1H, C6''-H), 7.67 (app. d, *J* 7.8 Hz, 1H, C4''-H), 7.48 (app. t, *J* 7.8 Hz, 1H, C5''-H), 7.40 (app. d, *J* 7.6 Hz, 2H, C2'-H), 7.32-7.37 (m, 4H, C3'-H, C4'''-H, C6'''-H), 7.26 (app. t, *J* 7.6 Hz, 1H, C4'-H), 7.09 (app. d, *J* 7.7 Hz, 1H, C3'''-H), 7.03 (app. td, *J* 7.7, 1.0 Hz, 1H, C5'''-H), 5.19 (dd, *J* 9.2, 4.8 Hz, 1H, C2-H), 3.81 (s, 3H, methoxy-CH_3), 3.51 (dd, *J* 14.3, 9.2 Hz, 1H, CH_2 syn or anti), 3.42 (dd, *J* 14.3, 4.8 Hz, 1H, CH_2 syn or anti), 1.37 (s, 9H, ^{t}Bu CH_3). **^{13}C NMR (125 MHz, MeOD-*d*$_4$)**; δ 168.6 (amide C=O), 158.0 (carbamate C=O), 156.6 (C2'''), 140.3 (C1'), 139.2 (C1''), 133.8 (C3''), 132.6 (C4''), 130.3 (C4'''), 129.7 (C1'''), 128.9 (C6'''), 128.2 (C3'), 128.0 (C2''), 127.7 (C5''), 127.1 (C4'), 126.3 (C2'), 125.3 (C6''), 120.6 (C5'''), 111.2 (C3'''), 79.1 ($C(CH_3)_3$), 55.6 (C2), 54.6 (methoxy-CH_3), 45.0 (C1), 27.3 (^{t}Bu CH_3).). **$\bar{\nu}_{max}$ /cm^{-1} (solid)**; 3063 (C-H), 1675 (carbamate C=O), 1633 (amide C=O). **HR-MS**, *m/z* (ES) found M+Na$^+$ 469.2101; $C_{27}H_{30}N_2O_4$ requires M+Na$^+$, 469.2103. **LC-MS**; RT = 0.70-0.75 min, *m/z* (ES) found 447.06.

Tert-butyl N-[(2S)-2-{[3-(3-ethoxyphenyl)phenyl]formamido}-2-phenylethyl]carbamate

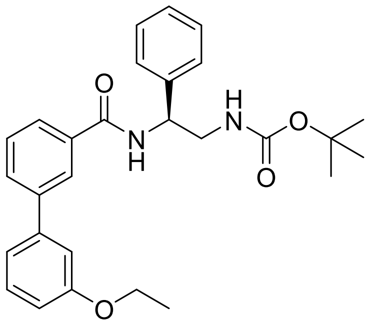

Synthesised *via* Method A. The crude product was purified using automatic flash column chromatography (0-25% ethyl acetate in petrol ether 40-60 ˚C) to yield the title compound as a colourless solid (35 mg, 0.08 mmol, 30%).

M.p. 113-115 °C. $\boldsymbol{R_f}$ 0.82 (50% ethyl acetate in petrol ether 40-60 ˚C). **^{1}H NMR (500 MHz, MeOD-*d*$_4$)**; δ 8.13 (s, 1H, C2''-H), 7.85 (app. d, *J* 7.8 Hz, 1H, C6''-H), 7.79 (app. d, *J* 7.8 Hz, 1H, C4''-H), 7.54 (app. t, *J* 7.8 Hz, 1H, C5''-H), 7.41 (app. d, *J* 7.6 Hz, 2H, C2'-H), 7.33-7.38 (m, 3H, C3'-H, C5'''-H), 7.21-7.29 (m, 3H, C4'-H, C2'''-H, C4'''-H), 6.93 (app. dd, *J* 8.5, 2.5 Hz, 1H, C6'''-H), 5.21 (dd, *J* 9.2, 4.6 Hz, 1H, C2-H), 4.11 (q, *J* 7.0 Hz, 2H, ethoxy-CH_2), 3.54 (dd, *J* 14.2, 9.2 Hz, 1H, CH_2 syn or anti), 3.44 (dd, *J* 14.2, 4.6 Hz, 1H, CH_2 syn or anti), 1.41 (t, *J* 7.0 Hz, 3H, ethoxy-CH_3), 1.38 (s, 9H, ^{t}Bu CH_3). **^{13}C NMR (125 MHz, MeOD-*d*$_4$)**; δ 168.4 (amide C=O), 159.6 (C3'''), 158.0 (carbamate C=O), 141.6 (C1'''), 141.5 (C3''), 140.2 (C1'), 134.6 (C1''), 129.9 (C4''), 129.6 (C5'''), 128.7 (C5''), 128.2 (C3'), 127.1 (C4'), 126.4 (C2'), 126.0 (C6''), 125.6 (C2''), 119.1 (C4'''), 113.4 (C6'''), 113.1 (C2'''), 79.1 ($C(CH_3)_3$), 63.2 (ethoxy-CH_2), 55.6 (C2), 45.0 (C1), 27.3 (^{t}Bu CH_3), 13.8 (ethoxy-CH_3). $\bar{\nu}_{max}$ **/cm^{-1} (solid)**; ~3400 (N-H), 2931 (C-H), 1685 (carbamate C=O), 1637 (amide C=O). **HR-MS**, *m/z* (ES) found M+Na$^+$ 483.2257; $C_{28}H_{32}N_2O_4$ requires M+Na$^+$, 483.2259. **LC-MS**; RT = 0.75-0.80 min, *m/z* (ES) found 461.10.

Tert-butyl N-[2-(3-hydroxyphenyl)-2-{[3-(2-methoxyphenyl)phenyl]formamido}ethyl]carbamate

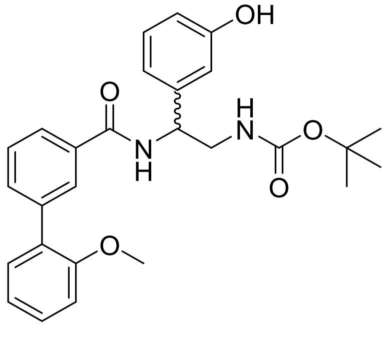

Synthesised *via* Method B. The crude residue was purified using automated flash column chromatography (0:100 – 10:90 MeOH:DCM) to yield the title compound as a colourless solid (46 mg, 0.10 mmol, 67%).

M.p. 81-83 °C. $\boldsymbol{R_f}$ 0.54 (5% MeOH in DCM). **^{1}H NMR (500 MHz, MeOD-*d*$_4$)**; δ 7.98 (app. s, 1H, C2''-H), 7.81 (app. d, *J* 7.8 Hz, 1H, C6''-H), 7.67 (app. dt, *J* 7.8, 1.2 Hz, 1H, C4''-H), 7.48 (app. t, *J* 7.8 Hz, 1H, C5''-H), 7.32-7.37 (m, 2H, C4'''-H, C6'''-H), 7.16 (app. t, *J* 7.8 Hz, 1H, C5'-H),

7.09 (app. d, *J* 8.0 Hz, 1H, C3'''-H), 7.03 (app. td, *J* 7.4, 1.0 Hz, 1H, C5'''-H), 6.87 (app. d, *J* 7.8 Hz, 1H, C6'-H), 6.84 (app. s, 1H, C2'-H), 6.69 (app. dd, *J* 7.8, 1.8 Hz, 1H, C4'-H), 5.11 (dd, *J* 7.6, 5.7 Hz, 1H, C2-H), 3.81 (s, 3H, methoxy-CH_3), 3.48 (dd, *J* 14.1, 9.4 Hz, 1H, CH_2 syn or anti), 3.40 (dd, *J* 14.1, 4.5 Hz, 1H, CH_2 syn or anti), 1.37 (s, 9H, ^{t}Bu CH_3). **^{13}C NMR (125 MHz, MeOD-*d*$_4$)**; δ 168.6 (amide C=O), 158.0 (carbamate C=O), 157.3 (C3'), 156.6 (C2'''), 141.8 (C1'), 139.2 (C1''), 133.8 (C3''), 132.6 (C4''), 130.3 (C4'''), 129.7 (C1'''), 129.2 (C5'), 128.9 (C6'''), 128.0 (C2''), 127.7 (C5''), 125.4 (C6''), 120.6 (C5'''), 117.3 (C6'), 114.0 (C4'), 113.2 (C2'), 111.2 (C3'''), 79.1 ($C(CH_3)_3$), 55.5 (C2), 54.6 (methoxy-CH_3), 44.9 (C1), 27.3 (^{t}Bu CH_3). **$\bar{\nu}_{max}$ /cm^{-1}**; 3345 (N-H), 2932 (C-H), 1677 (carbamate C=O), 1635 (amide C=O). **HR-MS**, *m/z* (ES) found M+Na$^+$ 485.2051; $C_{27}H_{30}N_2O_5$ requires M+Na$^+$, 485.2052. **LC-MS**; RT = 0.60-0.65 min, *m/z* (ES) found 463.44.

Tert-butyl N-[2-(4-hydroxyphenyl)-2-{[3-(2-methoxyphenyl)phenyl]formamido}ethyl]carbamate

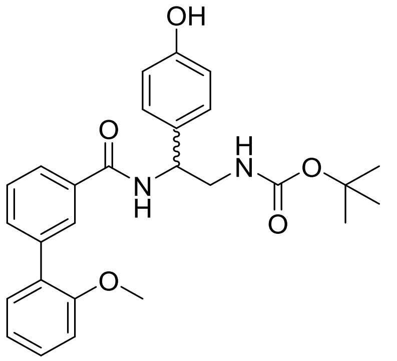

Synthesised *via* Method B. The crude residue was purified using automated flash column chromatography (0:100 – 10:90 MeOH:DCM) to yield the title compound as a colourless solid (34 mg, 0.07 mmol, 67%).

M.p. 98.5-100 °C. ***R*$_f$** 0.66 (5% MeOH in DCM). **^{1}H NMR (500 MHz, MeOD-*d*$_4$)**; δ 7.96 (app. s, 1H, C2''-H), 7.78 (app. d, *J* 7.8 Hz, 1H, C6''-H), 7.66 (app. dt, *J* 7.8, 1.2 Hz, 1H, C4''-H), 7.50 (app. t, *J* 7.8 Hz, 1H, C5''-H), 7.32-7.37 (m, 2H, C4'''-H, C6'''-H), 7.22 (app. d, *J* 8.5 Hz, 2H, C2'-H), 7.08 (app. d, *J* 7.8 Hz, 1H, C3'''-H), 7.03 (app. td, *J* 7.5, 1.0 Hz, 1H, C5'''-H), 6.76 (app. d, *J* 8.5 Hz, 2H, C3'-H), 5.11 (dd, *J* 9.2, 4.8 Hz, 1H, C2-H), 3.80 (s, 3H, methoxy-CH_3), 3.48 (dd, *J* 14.3, 9.4 Hz, 1H, CH_2 syn or anti), 3.36 (dd, *J* 14.3, 4.8 Hz, 1H, CH_2 syn or anti), 1.36 (s, 9H, ^{t}Bu CH_3). **^{13}C NMR (125 MHz, MeOD-*d*$_4$)**; δ 168.6 (amide C=O), 157.9 (carbamate C=O), 156.6 (C4'), 156.5 (C2'''), 139.2 (C1'), 133.9 (C1''), 132.5 (C4''), 131.0 (C3''), 130.3 (C4'''), 129.7 (C1'''), 128.8 (C6'''), 128.0 (C2''), 127.6 (C5''), 127.5 (C2'), 125.3 (C6''), 120.6 (C5'''), 114.9 (C3'), 111.2 (C3'''), 79.0 ($C(CH_3)_3$), 54.9 (C2), 54.6 (methoxy-CH_3), 45.0 (C1), 27.3 (^{t}Bu CH_3). **$\bar{\nu}_{max}$ /cm^{-1}**; 3276 (N-H), 2977 (C-H), 1675 (carbamate C=O), 1634 (amide C=O). **HR-MS**, *m/z* (ES) found M+Na$^+$ 485.2053; $C_{27}H_{30}N_2O_5$ requires M+Na$^+$, 485.2052. **LC-MS**; RT = 0.60-0.65 min, *m/z* (ES) found 463.44.

Tert-butyl N-(2-{[3-(3-ethoxyphenyl)phenyl]formamido}-2-(3-hydroxyphenyl)ethyl)carbamate

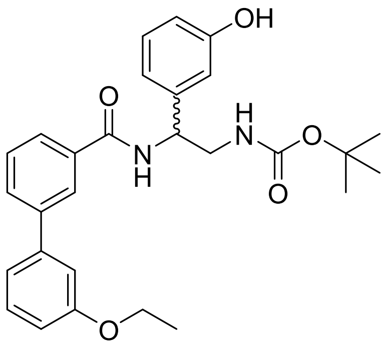

Synthesised *via* Method A. The crude product was purified using automated flash column chromatography (0:100 – 20:80 ethyl acetate:petrol ether 40-60 ˚C). 1 M TBAF in THF (680 µL, 0.68 mmol) was immediately added to a solution of the purified product in THF (10 mL). The mixture was stirred at RT for 20 hours. The solvent was stripped *in vacuo* and the residue purified using automated flash column chromatography (0:100 – 10:90 MeOH:DCM) to yield the title compound as a colourless foamy solid (40 mg, 0.08 mmol, 25%).

M.p. 62-63.5 °C. $\boldsymbol{R_f}$ 0.54 (5% MeOH in DCM). **^{1}H NMR (500 MHz, MeOD-*d*$_4$)**; δ 8.13 (app. s, 1H, C2”-H), 7.85 (app. d, *J* 7.8 Hz, 1H, C6”-H), 7.79 (app. d, *J* 7.8 Hz, 1H, C4”-H), 7.54 (app. t, *J* 7.8 Hz, 1H, C5”-H), 7.36 (app. t, *J* 7.8 Hz, 1H, C5’”-H), 7.25 (app. d, *J* 7.8 Hz, 1H, C6’”-H), 7.22 (app. s, 1H, C2’”-H), 7.17 (app. t, *J* 7.8 Hz, 1H, C5’-H), 6.93 (app. ddd, *J* 8.2, 3.2, 2.5 Hz, 1H, C4’”-H), 6.88 (app. d, *J* 7.8 Hz, 1H, C6’-H), 6.85 (app. s, 1H, C2’-H), 6.69 (app. dd, *J* 7.8, 1.9 Hz, 1H, C4’-H), 5.13 (dd, *J* 9.2, 4.5 Hz, 1H, C2-H), 4.14 (q, *J* 7.0 Hz, 2H, ethoxy-CH$_2$), 3.51 (dd, *J* 14.3, 9.2 Hz, 1H, CH$_2$ syn or anti), 3.42 (dd, *J* 14.3, 4.5 Hz, 1H, CH$_2$ syn or anti), 1.42 (t, *J* 7.0 Hz, 3H, ethoxy-CH$_3$), 1.38 (s, 9H, ^{t}Bu CH$_3$). **^{13}C NMR (125 MHz, MeOD-*d*$_4$)**; δ 168.4 (amide C=O), 159.6 (C3’), 158.0 (C3’”), 157.3 (carbamate C=O), 141.7 (C1’), 141.6 (C3”/C1’”), 141.5 (C3”/C1’”), 134.6 (C1”), 129.9 (C4”), 129.6 (C5’”), 129.2 (C5’), 128.7 (C5”), 126.0 (C6”), 125.6 (C2”), 119.1 (C6’”), 117.4 (C6’), 114.0 (C4’), 113.4 (C4’”), 113.2 (C2’), 113.0 (C2’”), 79.1 (C(CH$_3$)$_3$), 63.2 (ethoxy-CH$_2$), 55.6 (C2), 45.0 (C1), 27.3 (^{t}Bu CH$_3$), 13.0 (ethoxy-CH$_3$). $\bar{\nu}_{max}$ **/cm^{-1}**; 3284 (O-H), 2977 (C-H), 1682 (carbamate C=O), 1642 (amide C=O). **HR-MS**, *m/z* (ES) found M+Na$^+$ 499.2208; $C_{28}H_{32}N_2O_5$ requires M+Na$^+$, 499.2208. **LC-MS**; RT = 0.70-0.75 min, *m/z* (ES) found 377.34.

Tert-butyl N-(2-{[3-(3-ethoxy-2-fluorophenyl)phenyl]formamido}-2-(3-hydroxyphenyl)ethyl)carbamate

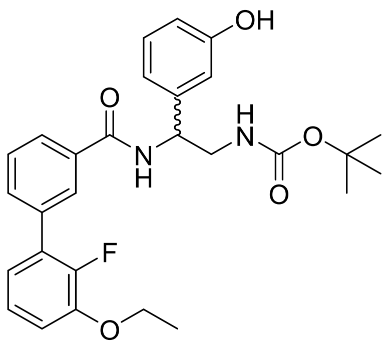

Synthesised *via* Method B. The crude residue was purified using automated flash column chromatography (0:100 – 10:90 MeOH:DCM) to yield the title compound as a colourless solid (19 mg, 0.04 mmol, 40%).

M.p. 63-65 °C. $\boldsymbol{R_f}$ 0.59 (5% MeOH in DCM). **^{1}H NMR (500 MHz, MeOD-*d*$_4$)**; δ 8.03 (app. s, 1H, C2''-H), 7.88 (app. d, *J* 7.8 Hz, 1H, C6''-H), 7.70-7.74 (m, 1H, C4''-H), 7.55 (app. t, *J* 7.8 Hz, 1H, C5''-H), 7.16 (app. t, *J* 7.8 Hz, 2H, C5'-H, C5'''-H), 7.04-7.12 (m, 2H, C4'''-H, C6'''-H), 6.87 (app. d, *J* 7.8 Hz, 1H, C6'-H), 6.84 (app. s, 1H, C2'-H), 6.69 (app. dd, *J* 7.8, 1.9 Hz, 1H, C4'-H), 5.12 (dd, *J* 9.0, 4.5 Hz, 1H, C2-H), 4.16 (q, *J* 7.0 Hz, 2H, ethoxy-CH_2), 3.49 (dd, *J* 14.1, 9.0 Hz, CH_2 syn or anti), 3.41 (dd, *J* 14.1, 4.5 Hz, 1H, CH_2 syn or anti), 1.44 (t, *J* 7.0 Hz, 3H, ethoxy-CH_3), 1.37 (s, 9H, ^{t}Bu CH_3). **^{13}C NMR (125 MHz, MeOD-*d*$_4$)**; δ 168.2 (amide C=O), 158.0 (C3'), 157.3 (carbamate C=O), 149.7 (d, ^1J_F 246 Hz, C2'''), 147.6 (d, ^2J_F 11.2 Hz, C3'''), 141.7 (C1'), 136.3 (C1''), 134.4 (C3''), 132.0 (d, ^4J_F 3.3 Hz, C4''), 129.2 (C5'), 128.9 (C1'''), 128.3 (C5''), 127.5 (C2''), 126.3 (C6''), 124.0 (d, ^4J_F 5.0 Hz, C5'''), 121.6 (d, ^3J_F 1.8 Hz, C6'''), 117.4 (C6'), 114.0 (C4''', C4'), 113.2 (C2'), 79.1 ($C(CH_3)_3$), 77.8 (ethoxy-CH_2) 64.7 (C2), 45.0 (C1), 27.3 (^{t}Bu CH_3). $\bar{\nu}_{max}$ **/cm^{-1} (solid)**; 3285 (N-H), 2929 (C-H), 1679 (carbamate C=O), 1643 (amide C=O). **HR-MS**, *m/z* (ES) found M+Na$^+$ 517.2111; $C_{28}H_{31}FN_2O_5$ requires M+Na$^+$, 517.2114. **LC-MS**; RT = 0.7-0.75 min, *m/z* (ES) found 395.33.

Tert-butyl N-(2-{[3-(3-ethoxy-2-fluorophenyl)phenyl]formamido}-2-(4-hydroxyphenyl)ethyl)carbamate

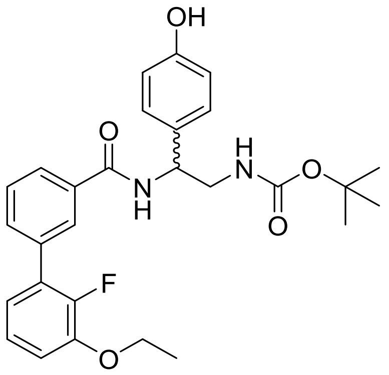

Synthesised *via* Method B. The crude residue was purified using automated flash column chromatography (0:100 – 10:90 MeOH:DCM) to yield the title compound as a colourless solid (6 mg, 0.01 mmol, 25%).

M.p. 92-94 °C. $\boldsymbol{R_f}$ 0.54 (5% MeOH in DCM). **^{1}H NMR (500 MHz, MeOD-*d*$_4$)**; δ 8.01 (app. s, 1H, C2''-H), 7.86 (app. d, *J* 7.8 Hz, 1H, C6''-H), 7.70 (app. d, *J* 7.8 Hz, 1H, C4''-H), 7.54 (app. t, *J* 7.8 Hz, 1H, C5''-H), 7.28 (app. d, *J* 8.2 Hz, 2H, C2'-H), 7.16 (app. td, *J* 8.2, 1.4 Hz, 1H, C5'''-H), 7.04-7.12 (m, 2H, C4'''-H, C6'''-H), 6.77 (app. d, *J* 8.2 Hz, 2H, C3'-H), 5.12 (dd, *J* 9.2, 4.6 Hz, 1H, C2-H), 4.15 (q, *J* 7.0 Hz, 2H, ethoxy-CH_2),

3.49 (dd, *J* 14.3, 9.2 Hz, 1H, C<u>H</u>$_2$ syn or anti), 3.36 (dd, *J* 14.3, 4.6 Hz, 1H, C<u>H</u>$_2$ syn or anti), 1.44 (t, *J* 7.0 Hz, 3H, ethoxy-C<u>H</u>$_3$), 1.37 (s, 9H, ^{t}Bu C<u>H</u>$_3$). **^{13}C NMR (125 MHz, MeOD-*d$_4$*)**; δ 168.2 (amide C=O), 157.9 (carbamate C=O), 156.6 (C4'), 149.6 (d, ^1J_F 246 Hz, C2'''), 147.5 (d, ^2J_F 11.2 Hz, C3'''), 136.2 (C1''), 134.5 (C3''), 132.0 (C4''), 130.9 (C1'), 128.9 (d, ^2J_F 11.2 Hz, C1'''), 128.3 (C5''), 127.6 (C2'', C2'), 126.3 (C6''), 124.0 (d, ^4J_F 5.0 Hz, C5'''), 121.6 (d, ^3J_F 1.8 Hz, C6'''), 114.9 (C3'), 114.0 (d, ^3J_F 1.8 Hz, C4'''), 79.0 (<u>C</u>(CH$_3$)$_3$), 64.7 (ethoxy-<u>C</u>H$_2$) 64.0 (C2), 45.0 (C1), 27.3 (^{t}Bu <u>C</u>H$_3$), 13.7 (ethoxy-<u>C</u>H$_3$). **$\bar{\nu}_{max}$ /cm^{-1} (solid)**; 3297 (N-H), 2926 (C-H), 1695 (carbamate C=O), 1645 (amide C=O). **HR-MS**, *m/z* (ES) found M+Na$^+$ 517.2109; $C_{28}H_{31}FN_2O_5$ requires M+Na$^+$, 517.2114. **LC-MS**; RT = 0.7-0.75 min, *m/z* (ES) found 395.33.

N-[(1R)-2-amino-1-phenylethyl]-[1,1'-biphenyl]-3-carboxamide hydrochloride (**2**)

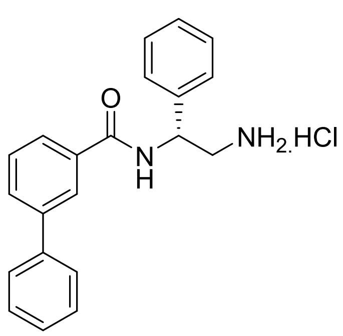

Synthesised initially *via* Method A. The resulting Boc-protected amino biphenyl was then carried forward crude *via* Method C. The crude residue was then purified using automated reverse phase flash column chromatography on C18 silica (0:100 – 100:0 MeCN:H$_2$O, 0.1% formic acid) to yield the title compound as a tan solid (9 mg, 0.03 mmol, 30% over two steps).

M.p. 146-148 °C. **^{1}H NMR (300 MHz, MeOD-*d$_4$*)**; δ 8.09 (t, *J* 1.7 Hz, 1H), 7.77 (dd, *J* 17.1, 7.7 Hz, 2H), 7.63 – 7.52 (m, 2H), 7.48 (t, *J* 7.7 Hz, 1H), 7.43 – 7.18 (m, 8H), 5.40 (s, 1H), 3.40 (m, 2H). **^{13}C NMR (101 MHz, MeOD-*d$_4$*)**; δ 169.0, 141.5, 140.0, 138.5, 134.3, 131.7, 130.1, 129.2 – 127.8 (m), 127.6 – 124.9 (m). **$\bar{\nu}_{max}$ /cm^{-1} (solid)**; 3296, 3031, 2923, 2789, 1630, 1573, 1524. **HR-MS**, *m/z* (ES) found M+H$^+$ 317.1634; $C_{26}H_{28}N_2O$ requires M+H$^+$ 317.1631.

N-[(1R)-2-amino-1-phenylethyl]-2'-methoxy-[1,1'-biphenyl]-3-carboxamide hydrochloride (**3**)

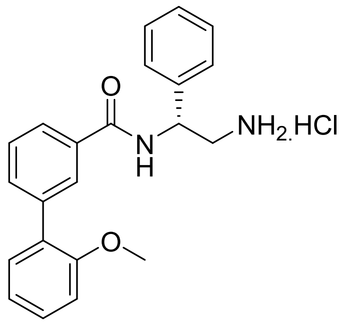

Synthesised initially *via* Method A. The resulting Boc-protected amino biphenyl was then carried forward crude *via* Method C. The crude residue was then purified using automated reverse phase flash column chromatography on C18 silica (0:100 – 100:0 MeCN:H_2O, 0.1% formic acid) to yield the title compound as a colourless solid (22 mg, 0.06 mmol, 24% over two steps).

^{1}H NMR (400 MHz, CDCl$_3$); δ 8.05 (br s, 3H, NH_3Cl), 7.65 – 7.60 (m, 2H), 7.45 – 7.40 (m, 2H), 7.30 – 7.10 (m, 8H), 6.92 – 6.85 (m, 2H), 5.50-5.40 (m, 1H, C1-H), 3.75 (s, 3H, OMe), 3.40-3.30 (m, 1H, CH_2 syn or anti), 3.20-3.10 (m, 1H, CH_2 syn or anti). No carbon NMR data was obtained. **HPLC** 2.56 min; 98%.

N-[(1R)-2-amino-1-phenylethyl]-3'-ethoxy-[1,1'-biphenyl]-3-carboxamide hydrochloride (**4**)

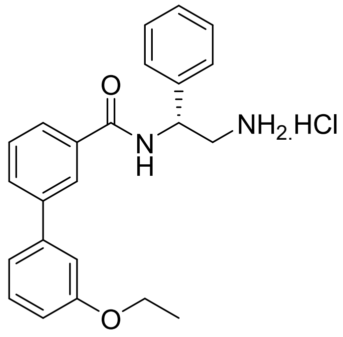

Synthesised initially *via* Method A. The resulting Boc-protected amino biphenyl was then carried forward crude *via* Method C. The crude residue was then purified using automated reverse phase flash column chromatography on C18 silica (0:100 – 100:0 MeCN:H_2O, 0.1% formic acid) to yield the title compound as a colourless solid (31 mg, 0.09 mmol, 39% over two steps).

^{1}H NMR (400 MHz, CDCl$_3$); δ 8.52 (br s, 1H, NH), 8.10 (br s, 1H, NH), 7.92 (d, 1H), 7.65 (br s, 1H, NH), 7.49 (d, 1H), 7.25 – 7.10 (m, 8H), 6.97 (2H), 6.78 (1H), 5.48 (br s, 1H, C1-H), 3.95 (q, 2H, OCH_2CH_3), 3.40 (br s, 1H, CH_2 syn or anti), 3.15 (br s, 1H, CH_2 syn or anti), 1.3 (t, 3H, OCH_2CH_3). No carbon NMR data was obtained. **HPLC** 2.37 min; 91%.

N-[(1R)-2-amino-1-phenylethyl]-2'-(trifluoromethyl)-[1,1'-biphenyl]-3-carboxamide hydrochloride (**5**)

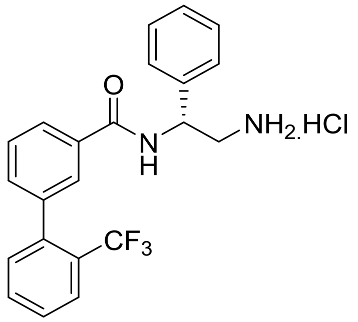

Synthesised *via* Method C. Off-white solid (5 mg, 0.01 mmol, 65%).

M.p. 126-128 °C. $\boldsymbol{R_f}$ 0.53 (10% MeOH in DCM). **^{1}H NMR (500 MHz, MeOD-*d*$_4$)**; δ 7.99 (app. d, *J* 7.2 Hz, 1H, C4''-H), 7.88 (app. s, 1H, C2''-H), 7.80 (app. d, *J* 7.8 Hz, 1H, C6'''-H), 7.67 (app. t, *J* 7.4 Hz, 1H, C4'''-H), 7.58 (app. t, *J* 7.8 Hz, 1H, C5'''-H), 7.50-7.55 (m, 2H, C6''-H, C5''-H), 7.48 (app. d, *J* 7.4 Hz, 2H, C2'-H), 7.39-7.44 (m, 3H, C3'-H, C3'''-H), 7.35 (app. t, *J* 7.0 Hz, 1H, C4'-H), 5.46-5.52 (m, 1H, C1-H), 3.45-3.54 (m, 1H, CH_2 syn or anti), 3.38-3.45 (m, 1H, CH_2 syn or anti). **^{13}C NMR (125 MHz, MeOD-*d*$_4$)**; δ 168.6 (C=O), 140.3 (C3''), 138.2 (C1'), 133.3 (C1'''), 132.3 (C5''), 131.9 (C3'''), 131.7 (C4'''), 128.8 (C3'), 128.2 (C4'), 128.0 (C2''), 127.9 (C4''), 127.8 (C5'''), 126.6 (C6''), 126.5 (C2'), 125.8 (q, ^4J_F 5.4 Hz, C5'''), 124.2 (q, ^1J_F 272 Hz, CF_3), 66.8 (C1), 43.3 (C2), C1'' unobserved. $\bar{\nu}_{max}$ **/cm^{-1} (solid)**; 2930 (C-H), 1633 (C=O). **HR-MS**, *m/z* (ES) found M+H$^+$ 385.1518; $C_{22}H_{19}F_3N_2O$ requires M+H$^+$, 385.1527. **HPLC** 2.54 min; 98%.

N-[(1R)-2-amino-1-phenylethyl]-3'-ethoxy-2'-fluoro-[1,1'-biphenyl]-3-carboxamide hydrochloride (**6**)

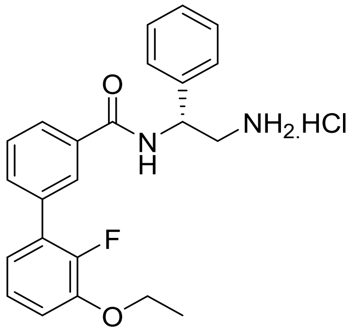

Synthesised *via* Method C. Colourless solid (7 mg, 0.02 mmol, 93%).

M.p. 195.5-197 °C. $\boldsymbol{R_f}$ 0.53 (10% MeOH in DCM). **^{1}H NMR (500 MHz, MeOD-*d*$_4$)**; δ 8.07 (app. s, 1H, C2''-H), 7.90-7.94 (m, 1H, C4''-H), 7.73 (app. dd, *J* 7.7, 1.0 Hz, 1H, C6''-H), 7.57 (app. t, *J* 7.7 Hz, 1H, C5''-H), 7.46-7.50 (m, 2H, C2'-H), 7.43 (app. t, *J* 7.6 Hz, 2H, C3'-H), 7.36 (app. t, *J* 7.6 Hz, 1H, C4'-H), 7.17 (app. td, *J* 8.2, 1.6 Hz, 1H, C5'''-H), 7.11 (app. td, *J* 8.2, 1.6 Hz, 1H, C4'''-H), 7.05 (app. td, *J* 8.2, 1.8 Hz, 1H, C6'''-H), 5.50 (dd, *J* 10.3, 4.8 Hz, 1H, C1-H), 4.15 (q, *J* 6.9 Hz, 2H, ethoxy-CH_2), 3.49 (app. dd, *J* 13.0, 10.3 Hz, 1H, CH_2 syn or anti), 3.42 (dd, *J* 13.0, 4.8 Hz, 1H, CH_2 syn or anti), 1.44 (t, *J* 6.9 Hz, 3H, ethoxy-CH_3). **^{13}C NMR (125 MHz, MeOD-*d*$_4$)**; δ 168.8 (C=O), 150.6 (C3'''), 149.6 (d, ^1J_F 247 Hz, C2'''), 147.5 (d, ^2J_F 11.2 Hz, C3'''), 138.2 (C1'), 136.3 (C1''), 133.9 (C3''), 132.3 (d, ^4J_F 3.4 Hz, C6''), 128.8 (C3'), 128.3 (C4'), 128.2 (C5''), 127.8 (d, ^4J_F 2.6 Hz, C2''), 127.3 (C1'''), 126.6 (C2'), 124.0 (d, ^4J_F 4.7 Hz, C5'''), 121.4 (d, ^3J_F 2.0 Hz, C6'''), 114.0 (C4'''), 64.7 (ethoxy-CH_2), 52.0 (C1), 43.3 (C2), 13.7 (ethoxy-CH_3). $\bar{\nu}_{max}$ **/cm^{-1} (solid)**; 2885 (C-H), 1630 (C=O). **HR-**

MS, *m/z* (ES) found M+H$^+$ 379.1818; $C_{23}H_{23}FN_2O_2$ requires M+H$^+$, 379.1821. **HPLC** 2.50 min; 92%.

N-[(1R)-2-amino-1-phenylethyl]-3'-ethoxy-4'-fluoro-[1,1'-biphenyl]-3-carboxamide hydrochloride (**7**)

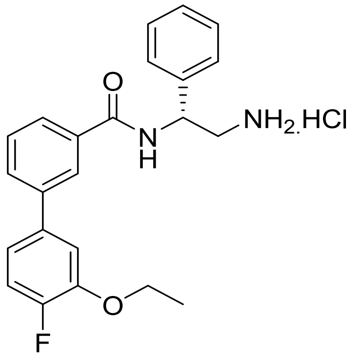

Synthesised *via* Method C. Colourless solid (12 mg, 0.03 mmol, 79%).

M.p. 219-221 °C. R_f 0.53 (10% MeOH in DCM). **^{1}H NMR (500 MHz, MeOD-d_4)**; δ 8.14 (app. t, *J* 1.6 Hz, C2''-H), 7.89 (app. d, *J* 8.0 Hz, 1H, C4''-H), 7.80 (app. d, *J* 8.0 Hz, 1H, C6''-H), 7.56 (app. t, *J* 8.0 Hz, 1H, C5''-H), 7.50 (app. d, *J* 7.3 Hz, 2H, C2'-H), 7.43 (app. t, *J* 7.3 Hz, 2H, C3'-H), 7.35-7.39 (m, 2H, C4'-H, C2'''-H), 7.20-7.24 (m, 1H, C6'''-H), 7.18 (app. dd, *J* 11.0, 8.5 Hz, 1H, C5'''-H), 5.51 (dd, *J* 10.2, 5.3 Hz, 1H, C1-H), 4.20 (q, *J* 7.0 Hz, 2H, ethoxy-CH_2), 3.52 (app. dd, *J* 13.2, 10.2 Hz, 1H, CH_2 syn or anti), 3.44 (dd, *J* 13.2, 5.3 Hz, 1H, CH_2 syn or anti), 1.44 (t, *J* 7.0 Hz, 3H, ethoxy-CH_3). **^{13}C NMR (125 MHz, MeOD-d_4)**; δ 169.0 (C=O), 152.6 (d, ^1J_F 245 Hz, C4'''), 147.2 (d, ^2J_F 11.1 Hz, C3'''), 140.8 (C1''), 138.2 (C1'), 136.9 (d, ^4J_F 3.9 Hz, C1'''), 134.2 (C3''), 130.2 (C6''), 128.8 (C3'), 128.7 (C5''), 128.2 (C4'), 126.6 (C2'), 126.1 (C4''), 125.8 (C2''), 119.4 (d, ^3J_F 7.7 Hz, C6'''), 115.9 (d, ^2J_F 18.6 Hz, C5'''), 113.7 (d, ^3J_F 2.0 Hz, C2'''), 64.8 (ethoxy-CH_2), 52.0 (C1), 43.3 (C2), 13.7 (ethoxy-CH_3). $\bar{\nu}_{max}$ **/cm^{-1} (solid)**; 3332 (N-H), 2975 (C-H), 1633 (C=O). **HR-MS**, *m/z* (ES) found M+H$^+$ 379.1817; $C_{23}H_{23}FN_2O_2$ requires M+H$^+$, 379.1821. **HPLC** 2.55 min; 100%.

N-[(1R)-2-amino-1-phenylethyl]-3'-hydroxy-[1,1'-biphenyl]-3-carboxamide hydrochloride (**8**)

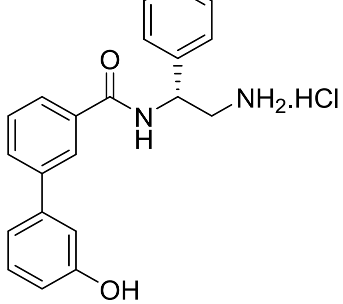

Synthesised *via* Method C. Colourless solid (25 mg, 0.07 mmol, 80%).

M.p. 188.5-190.0˚C. ***R*~f~** 0.04 (10% methanol in DCM). **^{1}H NMR (MeOD-*d~4~*);** δ 8.14 (app. t, *J* 1.69 Hz, 1H, C2''-H), 7.86-7.88 (m, 1H, C6''-H), 7.78-7.80 (m, 1H, C4''-H), 7.55 (app. t, *J* 7.8 Hz, 1H, C5''-H), 7.42 (app. d, *J* 7.8 Hz, 2H, C2'-H), 7.43 (app. t, *J* 7.8 Hz, 2H, C3'-H), 7.36-7.38 (m, 1H, C4'-H), 7.27 (app. t, *J* 7.8 Hz, 1H, C5'''-H), 7.13-7.15 (m, 1H, C6'''-H), 7.09 (app. t, 1H, C2'''-H), 6.79-6.82 (m, 1H, C4'''-H), 5.50 (dd, *J* 10.1, 4.9 Hz, 1H, C1-H), 3.50 (dd, *J* 10.1, 13.1 Hz, 1H, CH_2 syn or anti), 3.43 (dd, *J* 13.1, 4.9 Hz, 1H, CH_2 syn or anti). **^{13}C NMR (125 MHz, MeOD-*d~4~*);** δ 169.1 (amide C=O), 157.7 (C3'''), 141.6 (C1'), 141.5 (C3''), 138.2 (C1''), 134.2 (C1'''), 130.0 (C4''), 129.6 (C5'''), 128.8 (C5''), 128.7 (C3'), 128.2 (C4'), 126.5 (C2'), 126.1 (C6''), 125.8 (C2''), 117.9 (C6'''), 114.4 (C4'''), 113.5 (C2'''), 52.0 (C1), 43.2 (C2). **$\bar{\nu}_{max}$ /cm^{-1} (solid)**; 3368 (OH), 3334 (N-H), 2975 (C-H), 1648 (amide C=O). **HR-MS;** *m/z* (ES) found M+Na$^+$ 333.1595; $C_{21}H_{20}N_2O_2$ requires M+Na$^+$ 332.1524. **LC-MS;** RT = 0.20-0.40 min, *m/z* (ES) found 333.30. **HPLC** 2.00 min; 100%.

N-[(1R)-2-amino-1-phenylethyl]-3-(2-chlorophenyl)benzamide hydrochloride (**9**)

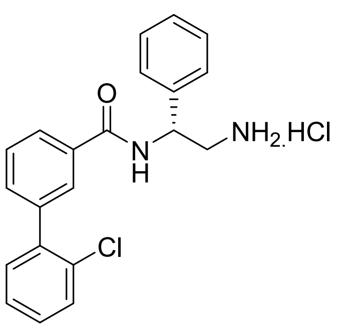

Synthesised *via* Method C. Colourless solid (15 mg, 0.03 mmol, 77%).

M.p. 160.5-163 °C. ***R*~f~** 0.45 (10% MeOH in DCM). **^{1}H NMR (500 MHz, MeOD-*d~4~*)**; δ 7.95 (s, 1H, C2''-H), 7.62-7.67 (m, 2H, C4''-H, C6''-H), 7.56 (app. t, *J* 7.5 Hz, 1H, C5''-H), 7.51 (app. dd, *J* 7.0, 1.6 Hz, 1H, C6'''-H), 7.48 (app. d, *J* 7.6 Hz, 2H, C2'-H), 7.33-7.44 (m, 5H, C3'-H, C3'''-H, C4'''-H, C5'''-H), 7.19-7.24 (m, 1H, C4'-H), 5.49 (dd, *J* 9.8, 4.5 Hz, 1H, C2-H), 3.46-3.52 (m, 1H, CH_2 syn or anti), 3.42 (dd, *J* 12.8, 4.2 Hz, 1H, CH_2 syn or anti). **^{13}C NMR (125 MHz, MeOD-*d~4~*)**; δ 168.7 (C=O), 139.9 (C1'), 139.6 (C3''), 138.1 (C1'''), 133.7 (C1''), 132.8 (C2'''), 132.4 (C4''), 131.1 (C3'''), 129.6 (C4'''), 129.0 (C6'''), 128.8 (C3'), 128.3 (C2''), 128.2 (C5''), 128.0 (C4'), 127.0 (C5'''), 126.6 (C2'-H), 126.5 (C6''), 52.0 (C1), 43.3 (C2). **$\bar{\nu}_{max}$ /cm^{-1} (solid)**; 3278 (N-H), 3032 (C-H), 1629 (C=O). **HR-MS**, *m/z* (ES) found M+H$^+$ 351.1256; $C_{21}H_{19}N_2OCl$ requires M+H$^+$, 351.1264. **HPLC** 2.43 min; 93%.

N-[(1R)-2-amino-1-phenylethyl]-3'-chloro-[1,1'-biphenyl]-3-carboxamide hydrochloride (**10**)

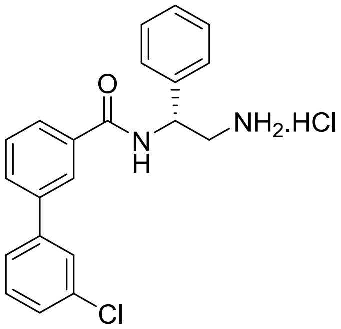

Synthesised *via* Method C. Colourless solid (15 mg, 0.04 mmol, 97%).

M.p. 208.0-211.0 ˚C. ***R*f** 0.25 (10% methanol in DCM). **^{1}H NMR (MeOD-*d4*);** δ 8.19 (app. t, *J* 1.67 Hz, 1H, C2''-H), 7.92-7.94 (m, 1H, C6''-H), 7.83-7.85 (m, 1H, C4''-H), 7.72 (app. t, *J* 1.67 Hz, 1H, C2'''-H), 7.62-7.64 (m, 1H, C4'''-H), 7.59 (app. t, *J* 7.8 Hz, 1H, C5''-H), 7.47-7.50 (m, 2H, C2'-H), 7.42-7.46 (m, 3H, C3'-H, C5'''-H), 7.38-7.40 (m, 1H, C6'''-H), 7.35-7.36 (m, 1H, C4'-H), 5.50 (dd, *J* 10.1, 4.8 Hz, 1H, C1-H), 3.53 (dd, *J* 13.1, 10.1 Hz, 1H, CH_2 syn or anti), 3.43 (dd, *J* 13.1, 4.8, 1H, CH_2 syn or anti). **^{13}C NMR (125 MHz, MeOD-*d4*);** δ 168.8 (amide C=O), 142.1 (C1'), 140.0 (C1'''), 138.1 (C3''), 134.5 (C1''), 134.3 (C3'''), 130.2 (C4''), 130.1 (C5'''), 129.0 (C5''), 128.8 (C3'), 128.2 (C4'), 127.4 (C6'''), 126.9 (C6''), 126.7 (C2'''), 126.6 (C2'), 125.8 (C2''), 125.2 (C4'''), 52.1 (C1), 43.3 (C2). **$\bar{\nu}_{max}$ /cm^{-1} (solid)**; 3291 (N-H), 2972 (C-H), 1641 (amide C=O). **HR-MS;** *m/z* (ES) accurate data could not be obtained; $C_{21}H_{19}ClN_2O$ requires M+Na$^+$ 350.1186 **LC-MS;** RT = 0.60-0.80 min, *m/z* (ES) found 351.28. **HPLC** 2.53 min; 100%.

N-[(1R)-2-amino-1-phenylethyl]-3'-nitro-[1,1'-biphenyl]-3-carboxamide hydrochloride (**11**)

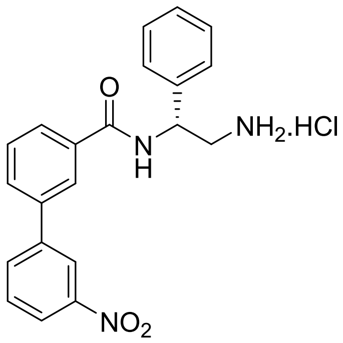

Synthesised *via* Method C. Colourless solid (12.0 mg, 0.03 mmol, 42%).

M.p. 148.5-150.0 ˚C. ***R*f** 0.03 (10% methanol in DCM). **^{1}H NMR (MeOD-*d4*);** δ 8.57 (app. t, *J* 1.8 Hz, 1H, C2'''-H), 8.25-8.28 (m, 2H, C4'''-H, C2''-H), 8.11-8.13 (m, 1H, C6'''-H), 7.98-7.99 (m, 1H, C4''-H), 7.89-7.94 (m, 1H, C6''-H), 7.75 (app. t, *J* 7.8 Hz, 1H, C5'''-H), 7.65 (app. t, *J* 7.8 Hz, 1H, C5''-H), 7.49-7.51 (m, 2H, C2'-H), 7.42-7.45 (m, 2H, C3'-H), 7.36 (app. t, *J* 7.7 Hz, 1H, C4'-H), 5.52 (dd, *J* 10.2, 4.8 Hz, 1H, C1-H), 3.54 (dd, *J* 10.2, 14.1 Hz, 1H, CH_2 syn or anti), 3.45 (dd, *J* 14.1, 4.8, 1H, CH_2 syn or anti). **^{13}C NMR (125 MHz, MeOD-*d4*);** δ 168.6 (amide C=O), 148.9 (C3'''), 141.8 (C1'), 139.1 (C3''), 138.1 (C1'''), 34.5 (C1''), 132.9 (C6'''), 130.4 (C6''), 130.0 (C5'''), 129.2 (C5''), 128.8 (C3'), 128.2 (C4'), 127.4 (C4''), 126.6 (C2'), 126.0 (C2''), 122.1 (C4'''), 121.4 (C2'''), 52.1 (C2), 43.3 (C1). **$\bar{\nu}_{max}$ /cm^{-1} (solid)**; 3323 (N-H), 2871 (C-H), 1643 (amide C=O), 1518 (nitro NO_2). **HR-MS;** *m/z* (ES) found M+H$^+$ 362.1489; $C_{21}H_{19}N_3O_3$ requires M+H$^+$ 362.1504. **LC-MS;** RT = 0.40-0.60 min, *m/z* (ES) found 362.34. **HPLC** 2.00 min; 100%.

3'-acetyl-N-[(1R)-2-amino-1-phenylethyl]-[1,1'-biphenyl]-3-carboxamide hydrochloride (**12**)

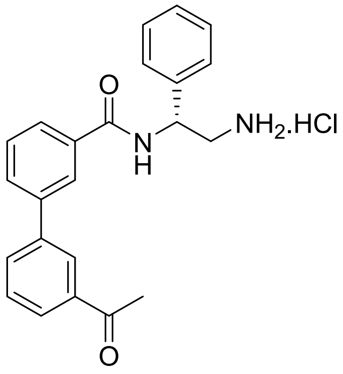

Synthesised *via* Method C. Colourless solid (21 mg, 0.05 mmol, 54%).

M.p. ~238 ˚C (decomposed from hexane). $\boldsymbol{R}_f$ 0.18 (10% methanol in DCM). **^{1}H NMR (MeOD-*d₄*);** δ 8.29 (app. t, *J* 1.6 Hz, 1H, C2’’’-H), 8.23 (app. t, *J* 1.6 Hz, 1H, C2”-H), 8.03-8.04 (m, 1H, C4’’’-H), 7.92-7.96 (m, 2H, C5’’’-H, C6’’’-H), 7.88-7.90 (m, 1H, C4”-H), 7.62 (app. quartet, *J* 7.6 Hz, 2H, C5”-H, C6”-H), 7.50 (app. d, *J* 7.6 Hz, 2H, C2’-H), 7.44 (app. t, *J* 7.6 Hz, 2H, C3’-H), 7.37 (app. t, *J* 7.5 Hz, 1H, C4’-H), 5.50 (dd, *J* 10.1, 5.1 Hz, 1H, C1-H), 3.50 (dd, *J* 13.4, 10.1, 1H, CH_2 syn or anti), 3.43 (dd, *J* 13.4, 5.1 Hz, 1H, CH_2 syn or anti), 2.68 (s, 3H, ketone-CH_3). **^{13}C NMR (125 MHz, MeOD-*d₄*);** δ 198.9 (ketone C=O), 168.8 (amide C=O), 140.7 (C1’), 140.5 (C3”), 138.1 (C1’’’), 137.7 (C1”), 134.4 (C3’’’), 131.6 (C6’’’), 130.3 (C4”), 129.1 (C6”), 129.0 (C5”), 128.8 (C3’), 128.2 (C4’), 127.6 (C4’’’), 126.7 (C5’’’), 126.6 (C2’), 126.3 (C2’’’), 125.9 (C2”), 55.5 (C1), 43.3 (C2), 25.5 (ketone-CH_3). $\bar{\nu}_{max}$ **/cm^{-1} (solid)**; 3319 (N-H), 2969 (C-H), 1683 (amide C=O), 1627 (ketone C=O). **HR-MS**; *m/z* (ES) found M+H$^+$ 359.1751; $C_{23}H_{11}N_2O_2$ requires M+H$^+$ 358.1681. **LC-MS;** RT = 0.60-0.80 min, m/z found 359.33. **HPLC** 4.91 min; 100%.

Methyl (R)-3'-{(2-amino-1-phenylethyl)carbamoyl}-[1,1'-biphenyl]-3-carboxylate hydrochloride (**13**)

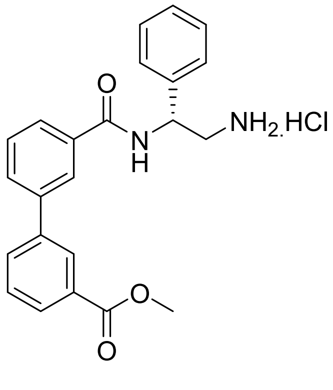

Synthesised *via* Method C. Title compound obtained as a colourless solid (15 mg, 0.04 mmol, 76%).

M.p. 212-215 °C. $\boldsymbol{R}_f$ 0.45 (10% MeOH in DCM). **^{1}H NMR (500 MHz, MeOD-*d₄*)**; δ 8.32 (t, *J* 1.7 Hz, 1H, C2’’’-H), 8.21 (t, *J* 1.7 Hz, 1H, C2”-H), 8.03 (dt, *J* 7.8, 1.4 Hz, 1H, C4’’’-H), 7.90-7.97 (m, 2H, C6”-H, C6’’’-H), 7.86 (ddd, *J* 7.7, 1.9, 1.0 Hz,

1H, C4”-H), 7.60 (td, *J* 7.8, 3.4 Hz, 2H, C5”-H, C5’”-H), 7.47-7.52 (m, 2H, C2’-H), 7.39-7.45 (t, *J* 7.7 Hz, 2H, C3’-H), 7.32-7.39 (m, 1H, C4’-H), 5.51 (dd, *J* 10.0, 4.7 Hz, 1H, C1-H), 3.94 (s, 3H, CH_3), 3.51 (dd, *J* 13.1, 10.1 Hz, 1H, C2-H syn or anti), 3.43 (dd, *J* 13.1, 4.8 Hz, 1H, C2-H syn or anti). **^{13}C NMR (125 MHz, MeOD-*d*$_4$)**; δ 168.8 (amide C=O), 166.9 (ester C=O), 140.6 (C3”/C1’”), 140.4 (C3”/C1’”), 138.2 (C1’), 134.4 (C1”), 131.5 (C6”), 130.8 (C3’”), 130.3 (C4”), 129.0 (C5”, C5’”), 128.8 (C3’), 128.4 (C4’”), 128.2 (C4’), 127.6 (C2’”), 126.7 (C6’”), 126.6 (C2’), 125.9 (C2”), 52.1 (C2), 51.4 (ester CH_3), 43.2 (C1). **$\bar{\nu}_{max}$ /cm^{-1} (solid)**; 2976 (C-H), 1710 (ester C=O), 1629 (amide C=O). **HR-MS**, *m/z* (ES) found M+H$^+$ 375.1715, $C_{23}H_{22}N_2O_3$ requires M+H$^+$ 375.1703. **HPLC** 2.49 min; 99%.

Methyl (R)-3'-{(2-amino-1-phenylethyl)carbamoyl}-[1,1'-biphenyl]-2-carboxylate hydrochloride (**14**)

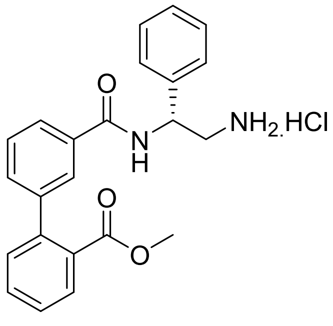

Synthesised *via* Method C. Title compound obtained as a colourless solid (5 mg, 0.01 mmol, 64%).

M.p. 145-147 °C. ***R*$_f$** 0.45 (10% MeOH in DCM). **^{1}H NMR (500 MHz, MeOD-*d*$_4$)**; δ 7.90 (d, *J* 7.5 Hz, 1H, C6”-H), 7.86 (s, 1H, C2”-H), 7.83 (dd, *J* 7.8, 1.2 Hz, 1H, C4”-H), 7.60 (td, *J* 7.6, 1.2 Hz, 1H, C3’”-H), 7.39-7.54 (m, 8H, C2’-H, C3’-H, C5”-H, C4’”-H, C5’”-H, C6’”-H), 7.35 (t, *J* 7.2 Hz, 1H, C4’-H), 5.48 (dd, *J* 9.9, 4.3 Hz, 1H, C1-H), 3.60 (s, 3H, CH_3), 3.37-3.51 (m, 2H, C2-H). **^{13}C NMR (125 MHz, MeOD-*d*$_4$)**; δ 168.9 (ester C=O), 168.8 (amide C=O), 141.7 (C3”/C1’”), 141.5 (C3”/C1’”), 138.2 (C1’), 133.5 (C1”), 131.8 (C6’”), 131.4 (C3’”), 130.6 (C2’”), 130.5 (C4’”), 129.6 (C4”), 128.8 (C3’), 128.2 (C4’), 128.0 (C5’”/C5”), 127.5 (C5’”/C5”), 127.3 (C6”), 126.6 (C2’), 126.1 (C2”), 52.0 (C2), 51.2 (ester CH_3), 43.3 (C1), **$\bar{\nu}_{max}$ /cm^{-1} (solid)**; 3333 (N-H), 2918 (C-H) V-shaped HCl salt-type signature, 1715 (ester C=O), 1636 (amide C=O). **HR-MS**, *m/z* (ES) found M+H$^+$ 375.1710, $C_{23}H_{22}N_2O_3$ requires M+H$^+$ 375.1703. **HPLC** 2.39 min; 98%.

N-[(1R)-2-amino-1-phenylethyl]-3-bromobenzamide hydrochloride (**15**)

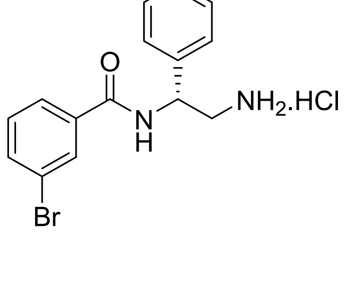

Synthesised *via* Method C. Colourless solid (34 mg, 0.11 mmol, 81% yield).

M.p. 238-241 ˚C. **^{1}H NMR (400 MHz, DMSO-*d_6*)**; δ 9.14 (d, *J* 8.3 Hz, 1H), 8.18 (d, *J* 1.9 Hz, 1H), 8.11 (s, 3H), 7.93 (d, *J* 7.8 Hz, 1H), 7.80 – 7.73 (m, 1H), 7.51 – 7.36 (m, 4H), 7.31 (t, *J* 7.1 Hz, 1H), 5.39 – 5.28 (m, 1H), 3.36 (m, 1H), 3.20 (dd, *J* 12.9, 3.9 Hz, 1H). **^{13}C NMR (101 MHz, DMSO-*d_6*)**; δ 165.4, 140.2, 136.7, 134.6, 130.8, 128.8 – 125.3 (m), 122.0, 52.0, 43.3. $\bar{\nu}_{max}$ **/cm^{-1} (solid)**; 3478, 3307, 3027, 2834, 2738, 2633, 1642, 1521. **HR-MS;** *m/z* (ES) found M+H$^+$ 319.0364, $C_{15}H_{17}BrN_2O$ requires M+H$^+$ 319.0359.

N-(2-Amino-1-phenylethyl)-2-methylbenzamide hydrochloride (**21**)

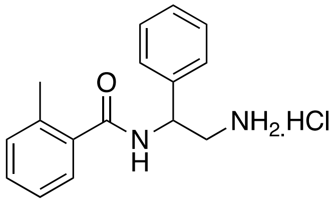

Synthesised by Method C. Colourless solid (40 mg, 0.14 mmol, 98%).

^{1}H NMR (400 MHz, MeOD-*d_4*) δ 7.50 (dt, *J* 8.6, 3.1 Hz, 3H, C2''-4''-H), 7.44 (t, *J* 7.4 Hz, 2H, C4'-H, C5''-H), 7.41 – 7.30 (m, 2H, C3'-H), 7.25 (t, *J* 7.2 Hz, 2H, C2'-H), 5.43 (dd, *J* 9.4, 5.2 Hz, 1H, C1-H), 3.46 (dd, *J* 13.0, 9.4 Hz, 1H, CH_2 syn or anti), 3.37 (dd, *J* 12.8, 5.3 Hz, 1H, CH_2 syn or anti), 2.31 (s, 3H, PhCH_3). **^{13}C NMR** (101 MHz, MeOD-*d_4*) δ 172.44 (C=O), 138.87 (C1'), 136.54 (C6''), 136.29 (C1''), 131.20 (C5''), 130.61 (C4''), 129.58 (C3''), 129.02 (C3'), 127.65 (C4'), 127.45 (C2'), 126.14 (C2''), 69.76 (C1), 43.88 (C2), 19.13 (PhCH_3). **HPLC**; 1.7 min, 96.9%.

N-(2-Amino-1-phenylethyl)-3-methylbenzamide hydrochloride (**22**)

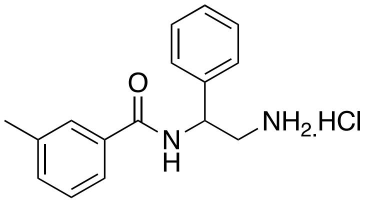

Synthesised by Method C. Colourless solid (20 mg, 0.07 mmol, 70%).

^{1}H NMR (400 MHz, MeOD-*d_4*) δ 7.78 – 7.68 (m, 2H, C2''-H, C6''-H), 7.51 – 7.30 (m, 7H, C2'-4'H, C3''-H, C4''-H), 5.47 (d, *J* 6.1 Hz, 1H, C1-H), 3.58 – 3.36 (m, 2H, CH_2), 2.40 (s, 3H, PhCH_3). **^{13}C NMR** (101 MHz, MeOD-*d_4*) δ 170.6 (C=O), 140.3 (C1'/C1''/C5''), 140.0 (C1'/C1''/C5''), 140.0 (C1'/C1''/C5''), 134.9 (C4''), 133.7 (C6''), 130.1 (C3'), 129.5 (C2'), 129.2 (C3''), 127.9 (C4'), 125.8 (C2''), 53.4 (C1), 44.7 (C2), 21.4 (PhCH_3). **HPLC**; 1.9 min, 95.2%.

N-(2-Amino-1-phenylethyl)-4-methylbenzamide hydrochloride (**23**)

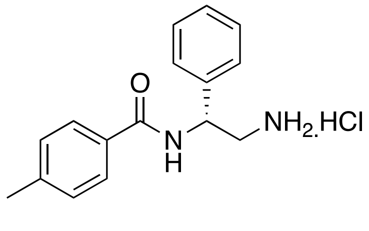

Synthesised by Method C. Colourless solid (44 mg, 0.15 mmol, 95%).

(25% ethyl acetate in hexane). **^{1}H NMR** (400 MHz, MeOD-*d_4*) δ 7.88 – 7.81 (m, 2H, C2''-H), 7.52 – 7.45 (m,

2H, C3'-H), 7.44 – 7.25 (m, 5H, C2'-H, C4'-H, C3''-H), 5.55 – 5.45 (m, 1H, C1-H), 3.54 (dd, *J* 13.1, 10.3 Hz, 1H, CH_2 syn or anti), 3.40 (dd, *J* 13.1, 4.6 Hz, 1H, CH_2 syn or anti), 2.38 (s, 3H, $PhCH_3$). **^{13}C NMR** (101 MHz, MeOD-*d_4*) δ 167.12 (C=O), 141.81 (C4''), 140.09 (C1'), 131.12 (C1''), 129.06 (C3''), 128.68 (C3'), 127.51 (C2''), 127.26 (C4'), 126.45 (C2'), 69.93 (C1), 45.71 (C2), 21.44 ($PhCH_3$). **HRMS**; *m/z* (ES) found $M+Na^+$ 277.1303, $C_{16}H_{18}N_2O$ requires $M+Na^+$ 277.1316. **HPLC**; 1.9 min, 98.9%.

N-[(1R)-2-amino-1-phenylethyl]-2'-methoxy-6-methyl-[1,1'-biphenyl]-3-carboxamide hydrochloride (**24**)

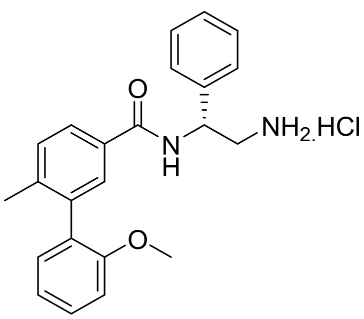

Synthesised *via* Method C. Off-white solid (6 mg, 0.02 mmol, 56%).

M.p. 156-158 °C. ***R*$_f$** 0.51 (10% MeOH in DCM). **^{1}H NMR (500 MHz, MeOD-*d_4*)**; δ 7.80 (app. d, *J* 7.0 Hz, 1H, C4''-H), 7.68 (app. s, 1H, C2''-H), 7.46 (app. d, *J* 7.4 Hz, C2'-H), 7.40 (app. t, *J* 7.4 Hz, C3'-H), 7.31-7.37 (m, 3H, C4'-H, C5''-H, C4'''-H), 7.12 (app. d, *J* 7.0 Hz, C6'''-H), 7.06 (app. d, *J* 8.4 Hz, 1H, C3'''-H), 7.01 (app. t, *J* 7.4 Hz, 1H, C5'''-H), 5.47 (app. d, *J* 7.1 Hz, 1H, C1-H), 3.73 (s, 3H, methoxy-CH_3), 3.43-3.51 (m, 1H, CH_2 syn or anti), 2.13 (s, 3H, C4''-CH_3). CH_2 syn or anti masked by MeOD-*d_4*. **^{13}C NMR (125 MHz, MeOD-*d_4*)**; δ 169.0 (C=O), 156.6 (C2'''), 141.5 (C1''), 139.3 (C1'), 138.4 (C6''), 130.7 (C3''), 130.3 (C6'''), 129.8 (C1'''), 129.3 (C5''), 128.9 (C2''), 128.8 (C4'''), 128.7 (C3'), 128.1 (C4'), 126.5 (C2'), 126.2 (C4''), 120.3 (C5'''), 110.6 (C3'''), 54.5 (methoxy-CH_3), 51.9 (C1), 43.3 (C2), 18.7 (C4''-CH_3). $\bar{\nu}_{max}$ **/cm^{-1} (solid)**; 2929 (C-H), 1631 (C=O). **HR-MS**, *m/z* (ES) found $M+H^+$ 361.1918; $C_{23}H_{24}N_2O_2$ requires $M+H^+$, 361.1916. **HPLC** 2.44 min; 93%.

N-[(1R)-2-amino-1-phenylethyl]-3'-ethoxy-6-methyl-[1,1'-biphenyl]-3-carboxamide hydrochloride (**25**)

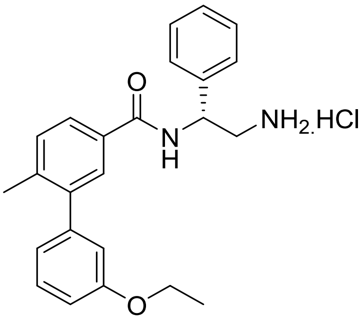

Synthesised *via* Method C. Off-white solid (6 mg, 0.02 mmol, 53%).

M.p. 118-120 °C. ***R*$_f$** 0.60 (10% MeOH in DCM). **^{1}H NMR (500 MHz, MeOD-*d_4*)**; δ 7.81 (app. d, *J* 7.8 Hz, 1H, C4''-H), 7.77 (app. s, 1H, C2''-H), 7.46 (app. d, *J* 7.7 Hz, 2H, C2'-H), 7.37-7.43 (m, 3H, C3'-H, C5''-H), 7.31-7.36 (m,

2H, C4’-H, C5’’’-H), 6.92 (app. dd, *J* 8.3, 2.1 Hz, 1H, C6’’’-H), 6.88 (app. d, *J* 7.7 Hz, 1H, C4’’’-H), 6.86 (app. s, 1H, C2’’’-H), 5.45-5.50 (m, 1H, C1-H), 4.06 (q, *J* 7.0 Hz, 2H, ethoxy-CH$_2$), 3.44-3.52 (m, 1H, CH$_2$ syn or anti), 3.39 (app. dd, *J* 12.0, 3.0 Hz, 1H, CH$_2$ syn or anti), 2.30 (s, 3H, C4”-CH$_3$), 1.39 (t, *J* 7.0 Hz, 3H, ethoxy-CH$_3$). **^{13}C NMR (125 MHz, MeOD-*d*$_4$)**; δ 168.9 (C=O), 159.0 (C3’’’), 142.3 (C1”), 142.1 (C1’’’), 139.8 (C1’), 138.3 (C6”), 131.1 (C3”), 130.2 (C5”), 129.0 (C5’’’), 128.8 (C3’), 128.4 (C2”), 128.1 (C4’), 126.6 (C2’), 126.2 (C4”), 121.0 (C4’’’), 115.1 (C2’’’), 112.9 (C6’’’), 63.2 (ethoxy-CH$_2$), 52.0 (C1), 43.0 (C2), 19.3 (C4”-CH$_3$), 13.8 (ethoxy-CH$_3$). **$\bar{\nu}_{max}$ /cm^{-1} (solid)**; 2977 (C-H), 1632 (C=O). **HR-MS**, *m/z* (ES) found M+H$^+$ 375.2065; $C_{24}H_{26}N_2O_2$ requires M+H$^+$, 375.2072. **HPLC** 2.65 min; 95%.

N-[(1R)-2-amino-1-phenylethyl]-3'-ethoxy-5',6-dimethyl-[1,1'-biphenyl]-3-carboxamide hydrochloride (**26**)

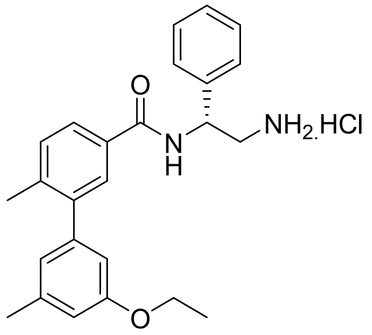

Synthesised *via* Method C. Colourless solid (6 mg, 0.02 mmol, 77%).

M.p. 127-129.5 °C. ***R*$_f$** 0.63 (10% MeOH in DCM). **^{1}H NMR (500 MHz, MeOD-*d*$_4$)**; δ 7.79 (app. dd, *J* 8.0, 1.7 Hz, 1H, C4”-H), 7.75 (app. d, *J* 1.7 Hz, 1H, C2”-H), 7.46 (app. d, *J* 7.6 Hz, 2H, C2’-H), 7.41 (app. t, *J* 7.6 Hz, 2H, C3’-H), 7.37 (app. d, *J* 8.0 Hz, 1H, C5”-H), 7.34 (app. t, *J* 7.6 Hz, 1H, C4’-H), 6.75 (app. s, 1H, C4’’’-H), 6.70 (app. s, 1H, C6’’’-H), 6.64 (app. s, 1H, C2’’’-H), 5.47 (dd, *J* 9.9, 4.5 Hz, 1H, C1-H), 4.03 (q, *J* 7.0 Hz, 2H, ethoxy-CH$_2$), 3.44-3.51 (m, 1H, CH$_2$ syn or anti), 3.39 (dd, *J* 13.1, 4.5 Hz, 1H, CH$_2$ syn or anti), 2.35 (s, 3H, C5’’’-CH$_3$), 2.29 (s, 3H, C4”-CH$_3$), 1.37 (t, *J* 7.0 Hz, 3H, ethoxy-CH$_3$). **^{13}C NMR (125 MHz, MeOD-*d*$_4$)**; δ 169.0 (C=O), 158.9 (C3’’’), 142.3 (C1”), 142.1 (C1’’’), 139.8 (C1’), 139.2 (C6”), 138.3 (C5’’’), 131.0 (C3”), 130.1 (C5”), 128.8 (C3’), 128.3 (C2”), 128.1 (C4’), 126.5 (C2’), 126.1 (C4”), 121.8 (C6’’’), 113.6 (C4’’’), 112.2 (C2’’’), 63.1 (ethoxy-CH$_2$), 52.0 (C1), 43.3 (C2), 20.2 (C5’’’-CH$_3$), 19.3 (C4”-CH$_3$), 13.8 (ethoxy-CH$_3$). **$\bar{\nu}_{max}$ /cm^{-1} (solid)**; 2975 (C-H), 1631 (C=O). **HR-MS**, *m/z* (ES) found M+H$^+$ 389.2224; $C_{25}H_{28}N_2O_2$ requires M+H$^+$, 389.2229. **HPLC** 2.80 min; 96%.

(R)-N-(2-Amino-1-phenylethyl)-5-(2-chlorophenyl)nicotinamide hydrochloride (**27**)

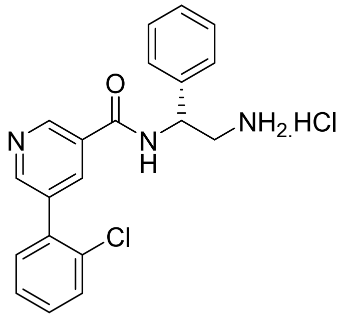

Synthesised *via* method C. Pink solid (10 mg, 0.026 mmol, 78%).

^{1}H NMR (500 MHz, MeOD-*d$_4$*); δ 9.49 (s, 1H, C4”-H), 9.22 - 9.21 (m, 2H, C2”- 6”-H), 7.70 – 7.65 (m, 2H, C4”’- 6”’-H), 7.58 – 7.55 (m, 4H, C3’- C3”’- C5”’-H), 7.43 (t, *J* 7.5 Hz, 2H, C2’-H), 5.55 (dd, *J* 10.5 4.2 Hz, 1H, C1-H), 3.65 (app. t, *J* 11.5 Hz, 1H, CH_2 syn or anti), 3.44 (dd, *J* 13.1 3.9 Hz, 1H, CH_2 syn or anti). **^{13}C NMR (125 MHz, MeOD-*d$_4$*)**; δ 164.2 (C=O), 146.6 (C4”), 145.9 (C6”), 142.5 (C1’), 139.1 (C2”), 134.3 (C1”’), 133.5 (C3”), 133.1 (C3”’), 133.0 (C6”’), 131.7 (C5”’), 130.4 (C3’), 130.3 (C2”’), 129.9 (C4”’), 129.4 (C4’), 128.4 (C2’), 128.3 (C1”). **HR-MS**, *m/z* (ES) found M+H$^+$ 352.1225; $C_{20}H_{18}ClN_3O$ requires M+H$^+$, 352.1217. **LC-MS**; RT = 0.5-0.6 min, *m/z* (ES) found 351.95. HPLC 2.14 min; 80%.

N-[(1S)-2-amino-1-phenylethyl]-3-phenylbenzamide hydrochloride (**28**)

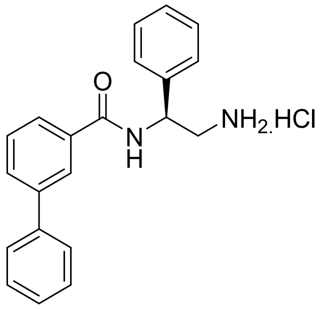

Synthesised *via* Method C. Colourless solid (8 mg, 0.03 mmol, 42%).

M.p. 205-207 °C. ***R*$_f$** 0.63 (10% MeOH in DCM). **^{1}H NMR (500 MHz, MeOD-*d$_4$*)**; δ 8.18 (app. t, *J* 1.8 Hz, 1H, C2”-H), 7.87-7.90 (m, 1H, C4”-H), 7.82-7.85 (m, 1H, C6”-H), 7.68 (app. dd, *J* 8.5, 1.4 Hz, 1H, C2”’-H), 7.57 (t, *J* 7.8 Hz, 1H, C5”-H), 7.42-7.51 (m, 6H, C2’-H, C3’-H, C3”’-H), 7.35-7.40 (m, 2H, C4’-H, C4”’-H), 5.51 (dd, *J* 10.0, 5.1 Hz, 1H, C1-H), 3.50 (dd, *J* 13.1, 10.0 Hz, 1H, CH_2 syn or anti), 3.44 (dd, *J* 13.1, 5.1 Hz, 1H, CH_2 syn or anti). **^{13}C NMR (125 MHz, MeOD-*d$_4$*)**; δ 169.1 (C=O), 141.6 (C3”), 140.1 (C1”’), 138.1 (C1’), 134.2 (C1”), 130.2 (C6”), 128.8 (C5”), 128.8 (C3”’), 128.6 (C3’), 128.2 (C4”’), 127.5 (C4’), 126.7 (C2”’), 126.6 (C2’), 126.1 (C4”), 125.8 (C2”), 52.0 (C1), 43.3 (C2). **$\bar{\nu}_{max}$ /cm^{-1} (solid)**; 2912 (C-H), 1632 (amide C=O). **HR-MS**, *m/z* (ES) found M+H$^+$ 317.1648; $C_{21}H_{21}N_2O$ requires M+H$^+$, 317.1653. **HPLC** 2.33 min; 98%.

N-[(1S)-2-amino-1-phenylethyl]-3-(2-methoxyphenyl)benzamide hydrochloride (**29**)

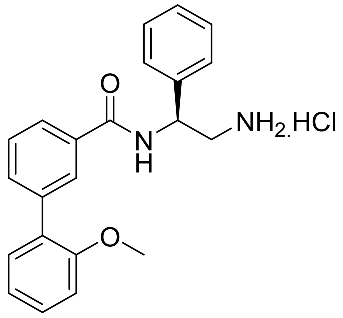

Synthesised *via* Method C. Colourless solid (12 mg, 0.03 mmol, 58%).

M.p. 127-129°C. ***R*$_f$** 0.63 (10% MeOH in DCM). **^{1}H NMR (500 MHz, MeOD-*d$_4$*)**; δ 8.02 (app. s, 1H, C2”-H), 7.86 (app.

d, *J* 7.8 Hz, 1H, C6”-H), 7.70 (app. d, *J* 7.8 Hz, 1H, C4”-H), 7.46-7.52 (m, 3H, C5”-H, C2’-H), 7.43 (app. t, *J* 7.3 Hz, 2H, C3’-H), 7.32-7.38 (m, 3H, C4’”-H, C6’”-H, C4’-H), 7.08 (app. d, *J* 7.8 Hz, 1H, C3’”-H), 7.03 (app. t, *J* 7.8 Hz, 1H, C5’”-H), 5.50 (dd, *J* 10.0, 4.8 Hz, 1H, C1-H), 3.79 (s, 3H, methoxy-CH_3), 3.49 (dd, *J* 13.1, 10.0 Hz, 1H, CH_2 syn or anti), 3.42 (dd, *J* 13.1, 4.8 Hz, 1H, CH_2 syn or anti). **^{13}C NMR (125 MHz, MeOD-*d*$_4$)**; δ 169.2 (C=O), 156.5 (C2’”), 139.3 (C1’), 138.2 (C1”), 133.4 (C3”), 133.0 (C4”), 130.3 (C4’”), 129.6 (C1’”), 129.0 (C6’”), 128.8 (C3’), 128.3 (C2”), 128.2 (C5”), 127.7 (C4’), 126.5 (C2’), 125.6 (C6”), 120.6 (C5’”), 111.2 (C3’”), 54.6 (methoxy-CH_3), 52.0 (C1), 43.3 (C2). $\bar{\nu}_{max}$ **/cm^{-1} (solid)**; 2922 (C-H), 1633 (C=O). **HR-MS**, *m/z* (ES) found M+H$^+$ 347.1752; $C_{22}H_{22}N_2O_2$ requires M+H$^+$, 347.1759. **HPLC** 2.33 min; 100%.

N-[(1S)-2-amino-1-phenylethyl]-3-(3-ethoxyphenyl)benzamide hydrochloride (**30**)

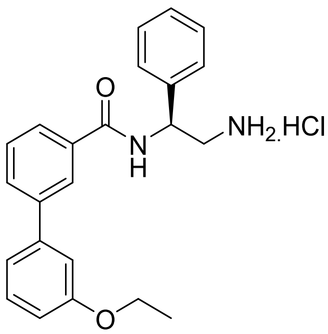

Synthesised *via* Method C. Colourless solid (18 mg, 0.05 mmol, 83%).

M.p. 213-215 °C. ***R*$_f$** 0.63 (10% MeOH in DCM). **^{1}H NMR (500 MHz, MeOD-*d*$_4$)**; δ 8.15 (app. t, *J* 1.8 Hz, 1H, C2”-H), 7.86-7.89 (m, 1H, C6”-H), 7.81-7.84 (m, 1H, C4”-H), 7.56 (app. t, *J* 7.6 Hz, 1H, C5”-H), 7.49 (app. d, *J* 7.4 Hz, 2H, C2’-H), 7.44 (app. t, *J* 7.4 Hz, 2H, C3’-H), 7.35-7.39 (m, 2H, C5’”-H, C4’-H), 7.22-7.25 (m, 1H, C6’”-H), 7.20 (app. t, *J* 2.3 Hz, 1H, C2’”-H), 6.93-6.96 (m, 1H, C4’”-H), 5.51 (dd, *J* 10.1, 5.1 Hz, 1H, C1-H), 4.11 (q, *J* 7.0 Hz, 2H, ethoxy-CH_2), 3.49 (dd, *J* 13.2, 10.1 Hz, 1H, CH_2 syn or anti), 3.44 (dd, *J* 13.2, 5.1 Hz, 1H, CH_2 syn or anti), 1.42 (t, *J* 7.0 Hz, 3H, ethoxy-CH_3). **^{13}C NMR (125 MHz, MeOD-*d*$_4$)**; δ 169.1 (C=O), 159.6 (C3’”), 141.5 (C1’”), 141.5 (C3”), 138.1 (C1’), 134.1 (C1”), 130.3 (C4”), 129.7 (C5’”), 128.8 (C3’), 128.7 (C5”), 128.2 (C4’), 126.6 (C2’), 126.1 (C6”), 125.8 (C2”), 119.0 (C4’”), 113.3 (C6’”), 113.2 (C2’”), 63.2 (ethoxy-CH_2), 52.0 (C1), 43.3 (C2), 13.8 (ethoxy-CH_3). $\bar{\nu}_{max}$ **/cm^{-1} (solid)**; 2923 (C-H), 1632 (C=O). **HR-MS**, *m/z* (ES) found M+H$^+$ 361.1907; $C_{23}H_{24}N_2O_2$ requires M+H$^+$, 361.1916. **HPLC** 2.52 min; 97%.

N-[2-amino-1-(3-hydroxyphenyl)ethyl]-3-(2-methoxyphenyl)benzamide hydrochloride (**31**)

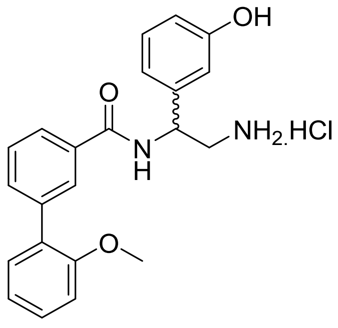

Synthesised *via* Method C. Off-white solid (31 mg, 0.08 mmol, 78%).

M.p. 205-208 °C. R_f 0.46 (10% MeOH in DCM). **^{1}H NMR (500 MHz, MeOD-*d*$_4$)**; δ 8.04 (app. s, 1H, C2''-H), 7.86 (app. d, *J* 7.8 Hz, 1H, C6''-H), 7.69 (app. d, *J* 7.8 Hz, 1H, C4''-H), 7.50 (app. t, *J* 7.8 Hz, 1H, C5''-H), 7.32-7.37 (m, 2H, C4'''-H, C6'''-H), 7.24 (app. t, *J* 7.8 Hz, 1H, C5'-H), 7.08 (app. d, *J* 8.0 Hz, 1H, C3'''-H), 7.03 (app. t, *J* 7.4 Hz, 1H, C5'''-H), 6.93 (app. d, *J* 7.8 Hz, 1H, C6'-H), 6.91 (app. s, 1H, C2'-H), 6.78 (app. dd, *J* 7.8, 1.8 Hz, 1H, C4'-H), 5.42 (dd, *J* 10.0, 4.6 Hz, 1H, C1-H), 3.79 (s, 3H, methoxy-CH_3), 3.45 (dd, *J* 13.0, 10.0 Hz, 1H, CH_2 syn or anti), 3.40 (dd, *J* 13.0, 4.6 Hz, 1H, CH_2 syn or anti).**^{13}C NMR (125 MHz, MeOD-*d*$_4$)**; δ 169.2 (amide C=O), 157.9 (C3'), 156.5 (C2'''), 139.6 (C1'), 139.2 (C1''), 133.4 (C3''), 133.0 (C4''), 130.3 (C4'''), 129.9 (C5'), 129.7 (C1'''), 128.9 (C6'''), 128.3 (C2''), 127.7 (C5''), 125.6 (C6''), 120.6 (C5'''), 117.3 (C6'), 115.0 (C4'), 113.4 (C2'), 111.2 (C3'''), 66.7 (C1), 54.7 (methoxy-CH_3), 43.3 (C1). **$\bar{\nu}_{max}$ /cm^{-1}**; ~3300 (N-H), ~2900 (C-H), 1630 (amide C=O). **HR-MS**, *m/z* (ES) found M+H$^+$ 363.1703; $C_{22}H_{22}N_2O_3$ requires M+H$^+$, 363.1708. **HPLC** 2.09 min; 97%.

N-[2-amino-1-(4-hydroxyphenyl)ethyl]-3-(2-methoxyphenyl)benzamide hydrochloride (**32**)

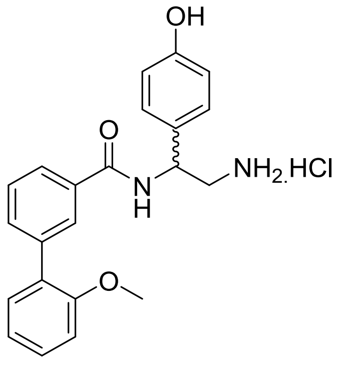

Synthesised *via* Method C. Off-white solid (23 mg, 0.06 mmol, 86%).

M.p. 156.158 °C. R_f 0.46 (10% MeOH in DCM). **^{1}H NMR (500 MHz, MeOD-*d*$_4$)**; δ 7.99 (app. s, 1H, C2''-H), 7.83 (app. d, *J* 7.8 Hz, 1H, C6''-H), 7.68 (app. d, *J* 7.8, 1H, C4''-H), 7.48 (app. t, *J* 7.8 Hz, 1H, C5''-H), 7.29-7.37 (m, 4H, C4'''-H, C6'''-H, C2'-H), 7.08 (app. d, *J* 7.8 Hz, 1H, C3'''-H), 7.02 (app. td, *J* 7.5, 1.0 Hz, 1H, C5'''-H), 6.83 (app. d, *J* 8.5 Hz, 2H, C3'-H), 5.39 (dd, *J* 9.5, 4.5 Hz, 1H, C1-H), 3.78 (s, 3H, methoxy-CH_3), 3.42-3.48 (m, 1H, CH_2 syn or anti), 3.36 (dd, *J* 12.5, 4.5 Hz, 1H, CH_2 syn or anti). **^{13}C NMR (125 MHz, MeOD-*d*$_4$)**; δ 169.2 (amide C=O), 157.5 (C4'), 156.5 (C2'''), 139.2 (C1'), 133.5 (C1''), 132.9 (C4''), 130.3 (C4'''), 129.6 (C1'''), 129.0 (C6'''), 128.7 (C3''), 128.3 (C2''), 127.9 (C2'), 127.7 (C5''), 125.5 (C6''), 120.6 (C5'''), 115.4 (C3'), 111.2 (C3'''), 66.7 (C1), 54.6 (methoxy-CH_3), 43.4 (C2). **$\bar{\nu}_{max}$ /cm^{-1}**; 3222 (N-H), 2937 (C-H), 1634 (amide C=O). **HR-MS**, *m/z* (ES) found M+H$^+$ 363.1707; $C_{22}H_{22}N_2O_3$ requires M+H$^+$, 363.1708. **HPLC** 2.04 min; 100%.

N-[2-amino-1-(3-hydroxyphenyl)ethyl]-3-(3-ethoxyphenyl)benzamide hydrochloride (**33**)

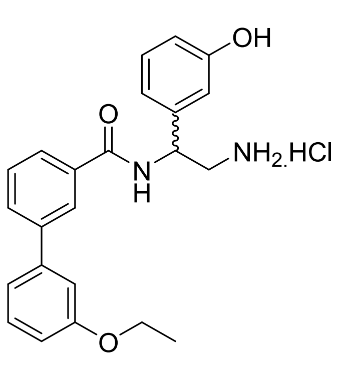

Synthesised *via* Method C. Off-white solid (20 mg, 0.05 mmol, 89%).

M.p. 213-215 °C. $\boldsymbol{R_f}$ 0.40 (10% MeOH in DCM). **^{1}H NMR (500 MHz, MeOD-*d*$_4$)**; δ 8.17 (app. t, *J* 1.7 Hz, 1H, C2”-H), 7.89 (app. d, *J* 7.8 Hz, 1H, C6”-H), 7.82 (app. d, *J* 7.8 Hz, 1H, C4”-H), 7.56 (app. t, *J* 7.8 Hz, 1H, C5”-H), 7.36 (app. t, *J* 7.8 Hz, 1H, C5’”-H), 7.22-7.27 (m, 2H, C6’”-H, C5’-H), 7.21 (app. t, *J* 2.1 Hz, 1H, C2’”-H), 6.90-6.96 (m, 3H, C4’”-H, C6’-H, C2’-H), 6.78 (app. dd, *J* 7.8, 2.0 Hz, 1H, C4’-H), 5.44 (dd, *J* 10.0, 4.9 Hz, 1H, C1-H), 4.11 (q, *J* 7.0 Hz, 2H, ethoxy-CH_2), 3.47 (dd, *J* 13.2, 10.0 Hz, 1H, CH_2 syn or anti), 3.42 (dd, *J* 13.2, 4.9 Hz, 1H, CH_2 syn or anti), 1.42 (t, *J* 7.0 Hz, 3H, ethoxy-CH_3). **^{13}C NMR (125 MHz, MeOD-*d*$_4$)**; δ 169.1 (C=O), 159.6 (C3’), 157.9 (C3’”), 141.5 (C1’, C3”), 139.6 (C1’”), 134.2 (C1”), 130.2 (C4”), 129.9 (C5’), 129.6 (C5’”), 128.7 (C5”), 126.2 (C6”), 125.9 (C2”), 119.0 (C6’”), 117.3 (C6’), 115.0 (C4’), 113.5 (C4’”), 113.4 (C2’), 113.1 (C2’”), 66.7 (C1), 63.2 (ethoxy-CH_2), 43.3 (C2), 13.8 (ethoxy-CH_3). $\bar{\nu}_{max}$ **/cm^{-1}**; ~3300 (N-H), ~3000 (C-H), 1639 (C=O). **HR-MS**, *m/z* (ES) found M+H$^+$ 377.1868; $C_{23}H_{24}N_2O_3$ requires M+H$^+$, 377.1865. **HPLC** 2.27 min; 98%.

N-[2-amino-1-(3-hydroxyphenyl)ethyl]-3-(3-ethoxy-2-fluorophenyl)benzamide hydrochloride (**34**)

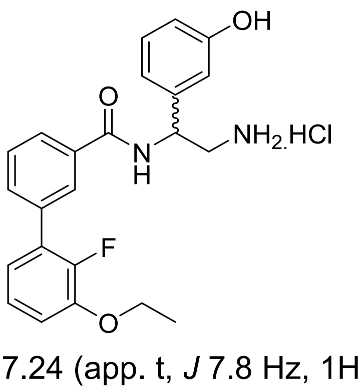

Synthesised *via* Method C. Off-white solid (10 mg, 0.02 mmol, 61%).

M.p. ~210 °C decomposed (from MeOH). $\boldsymbol{R_f}$ 0.46 (10% MeOH in DCM). **^{1}H NMR (500 MHz, MeOD-*d*$_4$)**; δ 8.08 (app. s, 1H, C2”-H), 7.93 (app. d, *J* 7.8 Hz, 1H, C6”-H), 7.73 (app. d, *J* 7.8 Hz, 1H, C4”-H), 7.57 (app. t, *J* 7.8 Hz, 1H, C5”-H), 7.24 (app. t, *J* 7.8 Hz, 1H, C5’-H), 7.17 (app. t, *J* 8.0 Hz, 1H, C5’”-H), 7.10 (app. td, *J* 8.0, 1.5 Hz, 1H, C4’”-H), 7.04-7.08 (m, 1H, C6’”-H), 6.94 (app. d, *J* 7.8 Hz, 1H, C6’-H), 6.91 (app. s, 1H, C2’-H), 6.78 (app. dd, *J* 7.8, 1.9 Hz, 1H, C4’-H), 5.42 (dd, *J* 10.0, 4.8 Hz, 1H, C1-H), 4.15 (q, *J* 7.0 Hz, 2H, ethoxy-CH_2), 3.46 (dd, *J* 13.0, 10.0 Hz, CH_2 syn

or anti), 3.40 (dd, *J* 13.0, 4.8 Hz, 1H, C<u>H</u>$_2$ syn or anti), 1.44 (t, *J* 7.0 Hz, 3H, ethoxy-C<u>H</u>$_3$). **^{13}C NMR (125 MHz, MeOD-*d$_4$*)**; δ 168.8 (amide C=O), 157.9 (C3'), 149.7 (d, ^1J_F 246 Hz, C2'''), 147.6 (d, ^2J_F 11.2 Hz, C3'''), 139.5 (C1'), 136.3 (C1''), 134.0 (C3''), 132.3 (d, ^4J_F 3.3 Hz, C4''), 129.9 (C5'), 128.8 (C1'''), 128.3 (C5''), 127.5 (d, ^4J_F 2.3 Hz, C2''), 126.5 (C6''), 124.0 (d, ^4J_F 5.0 Hz, C5'''), 121.5 (d, ^3J_F 1.8 Hz, C6'''), 117.3 (C6'), 115.0 (C4'), 114.0 (C4'''), 113.5 (C2'), 64.7 (C1), 43.3 (C2), ethoxy-<u>C</u>H$_2$ unobserved. **$\bar{\nu}_{max}$ /cm^{-1} (solid)**; ~3300 (N-H), ~2900 (C-H), 1634 (amide C=O). **HR-MS**, *m/z* (ES) found M+H$^+$ 395.1762; $C_{23}H_{23}FN_2O_3$ requires M+H$^+$, 395.1770. **HPLC** 2.26 min; 100%.

N-[2-amino-1-(4-hydroxyphenyl)ethyl]-3-(3-ethoxy-2-fluorophenyl)benzamide hydrochloride (**35**)

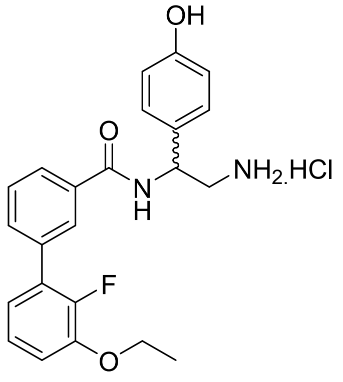

Synthesised *via* Method C. Off-white solid (4 mg, 8 x 10^{-3} mmol, 80%).

M.p. 148-150 °C. ***R*$_f$** 0.46 (10% MeOH in DCM). **^{1}H NMR (500 MHz, MeOD-*d$_4$*)**; δ 8.06 (app. s, 1H, C2''-<u>H</u>), 7.90 (app. d, *J* 7.8 Hz, 1H, C6''-<u>H</u>), 7.72 (app. d, *J* 7.8 Hz, 1H, C4''-<u>H</u>), 7.56 (app. t, *J* 7.8 Hz, 1H, C5''-<u>H</u>), 7.30 (app. d, *J* 8.2 Hz, 2H, C2'-<u>H</u>), 7.17 (app. t, *J* 8.2 Hz 1H, C5'''-<u>H</u>), 7.10 (app. t, *J* 8.2 Hz, 1H, C4'''-<u>H</u>), 7.05 (app. t, *J* 8.2 Hz, 1H, C6'''-<u>H</u>), 6.83 (app. d, *J* 8.2 Hz, 2H, C3'-<u>H</u>), 5.40 (dd, *J* 9.5, 4.8 Hz, 1H, C2-<u>H</u>), 4.15 (q, *J* 7.0 Hz, 2H, ethoxy-C<u>H</u>$_2$), 3.43-3.49 (m, 1H, C<u>H</u>$_2$ syn or anti), 3.37 (dd, *J* 13.0, 4.8 Hz, 1H, C<u>H</u>$_2$ syn or anti), 1.43 (t, *J* 7.0 Hz, 3H, ethoxy-C<u>H</u>$_3$). **^{13}C NMR (125 MHz, MeOD-*d$_4$*)**; δ 168.8 (amide C=O), 157.5 (C4'), 149.6 (d, ^1J_F 246 Hz, C2'''), 147.5 (d, ^2J_F 11.2 Hz, C3'''), 136.3 (C1''), 134.0 (C3''), 132.2 (C4''), 128.8 (d, ^2J_F 11.2 Hz, C1'''), 128.6 (C1'), 128.3 (C5''), 127.9 (C2'', C2'), 126.5 (C6''), 124.0 (d, ^4J_F 5.0 Hz, C5'''), 121.5 (d, ^3J_F 1.8 Hz, C6'''), 115.5 (C3'), 114.0 (d, ^3J_F 1.8 Hz, C4'''), 66.7 (C1), 64.7 (ethoxy-<u>C</u>H$_2$), 43.4 (C2), 13.7 (ethoxy-<u>C</u>H$_3$). **$\bar{\nu}_{max}$ /cm^{-1} (solid)**; 3199 (N-H), 2927 (C-H), 1634 (amide C=O). **HR-MS**, *m/z* (ES) found M+H$^+$ 395.1773; $C_{23}H_{23}FN_2O_3$ requires M+H$^+$, 395.1770. **HPLC** 2.21 min; 100%.

References

1. I. Wiegand, K. Hilpert, R. Hancock, *Nat. Protoc.*, 2008, **3**, 163-175.

2. J.M. Andrews, *Journal of Antimicrobial Chemotherapy*, 2001, **48**, 5-16.

3. Maestro, Schrödinger, LLC, New York, NY, 2021.

4. V. Gillet, A.P. Johnson, P. Mata, S. Sike, P. Williams, *Journal of Computer-Aided Molecular Design*, 1993**,** **7**, 127-153.

5. P.F. Chan, T. Germe, B.D. Bax, J. Huang, R.K. Thalji, E. Bacqué, A. Checchia, D. Chen, H. Cui, X. Ding, K. Ingraham, L. McCloskey, K. Raha, V. Srikannathasan, A. Maxwell, R.A. Stavenger, *Proceedings of the National Academy of Sciences*, 2017**,** **114**, E4492.
